# Assessing Physicians’ Knowledge, Attitudes, Intentions, Abilities, and Behaviour Toward Physical Activity and Exercise in Non-Communicable Diseases: Questionnaire Development Using an e-Delphi and Cross-Sectional Design

**DOI:** 10.3390/healthcare14091148

**Published:** 2026-04-24

**Authors:** Niki Syrou, Ioannis G. Fatouros, George S. Metsios, Athanasios Z. Jamurtas, Dimitrios Draganidis, Konstantinos G. Perivoliotis, Athanasios Poulios, Panagiotis Tsimeas, Konstantinos Papanikolaou, Theodore J. Angelopoulos, Ioannis Adamopoulos, George Mastorakos

**Affiliations:** 1 Department of Physical Education and Sport Science, University of Thessaly, Karies, 42100 Trikala, Greece; ifatouros@uth.gr (I.G.F.); ajamurt@pe.uth.gr (A.Z.J.); ddraganidis@pe.uth.gr (D.D.); apoulios@uth.gr (A.P.); ptsimeas@pe.uth.gr (P.T.); kpapanikolaou@uth.gr (K.P.); 2 School of Medicine, National and Kapodistrian University of Athens, 75 Mikras Asias, 11527 Athens, Greece; 3 Department of Nutrition and Dietetics, University of Thessaly, 1C Argonafton, 42132 Trikala, Greece; g.metsios@uth.gr; 4 School of Sport, Performing Arts and Leisure, University of Wolverhampton, Wulfruna Street, Wolverhampton WV1 1LY, UK; 5 Department of Surgery, General Hospital of Volos, 134 Polymeri, 38222 Volos, Greece; kperi19@gmail.com; 6 Department of Rehabilitation and Movement Science, University of Vermont, Burlington, VT 05405, USA; theodore.angelopoulos@med.uvm.edu; 7 Department of Public Health Policy, Sector of Occupational & Environmental Health, School of Public Health, University of West Attica, 196 Alexandra Avenue, 11521 Athens, Greece; adamopoul@gmail.com; 8 Department of Public Health & Policies, School of Social Science, Hellenic Open University, 18 Aristotelous St., 26335 Patras, Greece

**Keywords:** questionnaire construction and validation, e-Delphi study, physicians, perceptions, practices, physical activity and exercise, chronic diseases

## Abstract

**Background/Objectives**: The multiple benefits of physical activity and exercise (PAE) for non-communicable diseases (NCDs) and, thus, for public health underscore the importance of their multidisciplinary implementation in clinical practice. However, there is a lack of validated instruments that comprehensively assess physicians’ knowledge, attitudes, intentions, abilities, and behaviour (KAIAB) regarding PAE promotion in NCD management. **Methods**: This study aimed to develop and validate a new questionnaire to assess physicians’ KAIAB towards PAE and to evaluate their KAIAB levels. A two-stage design, including an e-Delphi method and a cross-sectional study, was conducted in Greece from January 2022 to May 2022. **Results**: In the first stage, after achieving consensus and stability within a purposive sample of 16 physician–experts (response rate 100%), the questionnaire was effectively developed and validated (Content Validity Ratio: 0.5–1) using a two-round e-Delphi method. In the second stage, a cross-sectional study was conducted in two physician populations from 12 medical specialities (response rate: 18.2%) and demonstrated that the new questionnaire had sufficient face validity and high reliability (Cronbach’s alpha: 0.805– 0.931). The three original Bloom levels’ cut-off points were also used to classify physicians’ KAIAB levels in the second stage. KAIAB levels were assessed using median and interquartile range (Mdn/IQR) and were found to be low (13/6), moderate (128/79), high (35/9), moderate (21/8), and moderate (33/8), respectively. **Conclusions**: The new questionnaire is reliable and valid. It is recommended that the questionnaire be applied in larger studies to further verify its validity and applicability. Additionally, it was found that although physicians reported high intentions and moderately positive attitudes toward PAE promotion, their knowledge in these domains and their exercise prescription practices remained limited. This underscores the need to enhance policies and initiatives in medical education and the healthcare system.

## 1. Introduction

Several studies demonstrate the benefits of Physical Activity and Exercise (PAE) in the prevention and management of chronic Non-Communicable Diseases (NCDs). These benefits include reduced mortality, comorbidity, and depression. PAE is also associated with improved quality of life and well-being. In addition, it contributes to lower healthcare costs and reduced social burden [1,2,3,4,5,6,7,8]. PAE is considered among the best available interventions and potentially the “best buy” and most cost-effective for reducing the prevalence of NCD risk factors [8,9]. Evidence supports the association between higher levels of Physical Activity (PA) and reduced risks of all-cause mortality, cardiovascular disease, and cancer mortality [10] and emphasises the health benefits of incidental PA, especially for individuals who face challenges engaging in structured exercise [11]. PAE is recommended as an adjunct to existing clinical approaches and as an important component of multidisciplinary care to better manage NCD risk and symptoms [12]. Evidence indicates that health professionals are aware of the benefits of PAE and consider it essential for managing NCDs and improving quality of life [13]. An increase in effective interventions designed to promote PA has also been established [14]. A recent systematic review and meta-analysis [15] investigated the effectiveness of PA interventions provided or recommended by primary healthcare professionals. The interventions aimed to increase moderate-to-vigorous-intensity PA in adult patients. The review concluded that these interventions increase self-reported physical activity [15].

The World Health Organization (WHO) states that one of the main roles and responsibilities of healthcare providers is to promote healthy lifestyles. This includes physical activity counselling, assessment, and referral to specialists. The WHO identifies these practices as the most cost-effective approach to management. It also recommends including physical activity as part of routine care [16]. As healthcare professionals are on the front lines of care, assessing and treating patients with NCDs, increasing their PA levels and reducing their sedentary behaviour through PAE counselling is important [17]. Thus, despite the positive impact of PA promotion on patients, implementing these interventions in routine clinical practice remains unrealised potential [18]. Integrating PA into standard medical care settings remains a significant gap [19,20,21]. The WHO reports that fewer than half of countries have national protocols for implementing PA in primary health care; however, 1/3 of countries use them in fewer than half of healthcare facilities [16]. According to the WHO PA fact sheet for Europe on the promotion of PA counselling and prescribing by health professionals, fewer than 70% of countries reported having national guidance or provision for this purpose [22].

PA promotion is hindered by specific barriers [23,24], the most significant of which is the lack of knowledge and training on PAE issues, as healthcare professionals’ curricula do not include PAE-specific education. The most common way health professionals promote PAE is through counselling [13,25,26]. PA on prescription as a treatment is also questionable because physicians are not trained in or familiar with non-pharmaceutical approaches [27]. Another barrier is limited counselling time [13,25,26]. Brief counselling is a time-efficient and effective way to promote PAE [19]. Any qualified healthcare provider, including nurses and primary care physicians, can provide brief interventions that increase patients’ PA levels [18]. A recent systematic review found that brief PA promotion interventions delivered in healthcare settings led to a medium-term increase in PA [28].

From time to time, studies have investigated the perceptions, needs, barriers, and practices of physicians and other health professionals regarding PAE and have considered it an economical, non-pharmaceutical approach to preventing and managing NCDs in healthcare settings [13,19,29]. Most studies investigate PAE knowledge, perceptions, and practices among general practitioners or physicians managing all patients, but none among physicians in 12 specialities in primary and secondary healthcare. Furthermore, no studies have been found that simultaneously explore, using a single research tool, the knowledge, attitudes, intentions, abilities, and behaviour (KAIAB) towards PAE among physicians from 12 specialities managing patients with NCDs. It is also worth noting that most existing studies investigating physicians’ knowledge, attitudes, and practices toward PAE have largely relied on non-standardised, ad hoc questionnaire instruments. Μany PA-related instruments lack an explicit conceptual framework. This can be a problem because it raises questions about the instrument’s validity and intelligibility. Highlighting the importance of conceptual frameworks will increase awareness that instruments can benefit from them, leading to higher-quality instruments and more relevant measures. For example, Gimeno-Santos et al. conducted a systematic review and reported that many of the physical activity questionnaires reviewed lacked a conceptual framework, hindering their ability to adequately represent the multifaceted nature of PA constructs [30]. Without a theoretical base, there could be confusion about which constructs are being assessed and to what extent measurements are comparable across studies. One further argument is that, even though there are many multi-validated measures, they assess only one determinant of behaviour (e.g., PA level or acceptance) rather than several. An example of this is the Physical Activity Assessment Tool (PAAT), which was developed as a clinical assessment tool but was designed to measure activity rather than behavioural dimensions [31]. Single domains, such as attitudes, have also been identified [32].

This study aims to develop and validate a new questionnaire to assess physicians’ KAIAB towards PAE across 12 specialities, and to evaluate and correlate their KAIAB levels. Healthcare systems vary across countries, and physicians worldwide face challenges in promoting PAE to their patients, including time constraints, limited knowledge, and training and skill gaps. As the first step in solving a problem is to identify it, this new questionnaire is the first in the literature to provide a comprehensive assessment of the perceptions and practices of PAE promotion among 12 primary and secondary healthcare physicians managing patients with NCDs and to enhance the interaction between research and future policies and initiatives on physicians’ PAE education and training.

## 2. Materials and Methods

### 2.1. Study Design and Study Population

**Figure 1:**
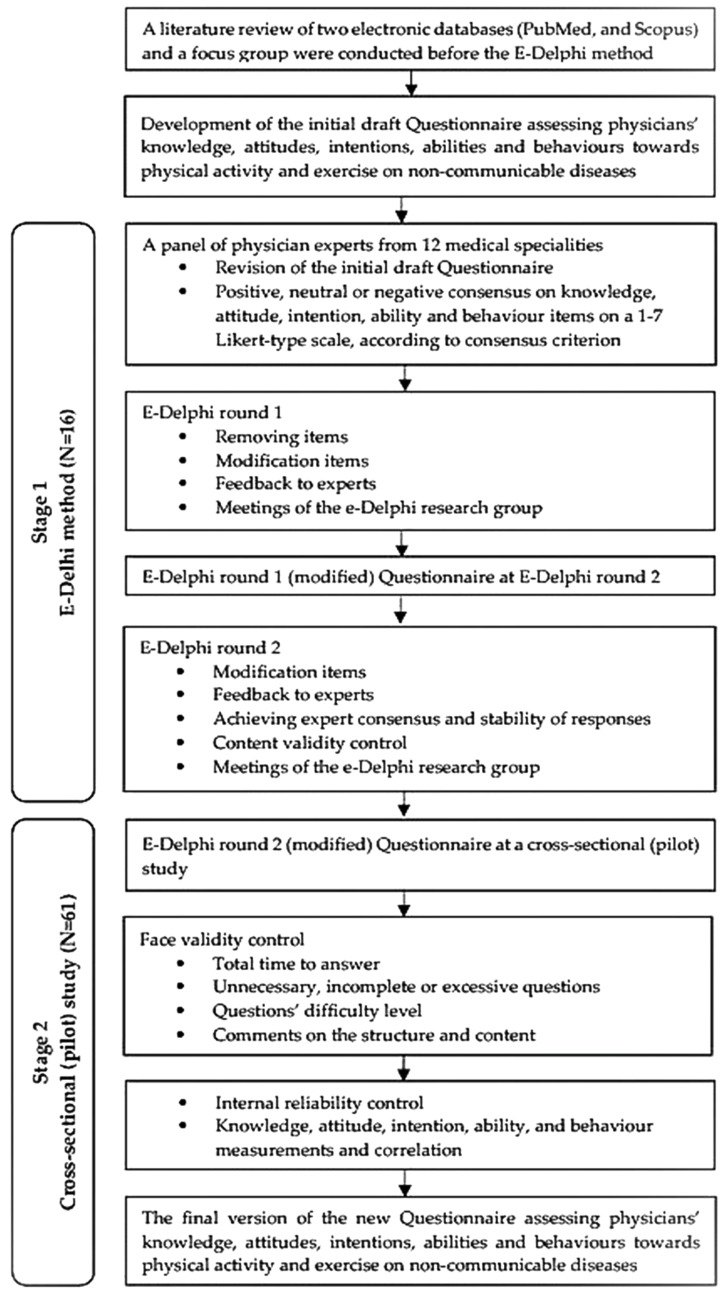
The study’s flow diagram.

A two-stage, observational design study, including an e-Delphi method and a cross-sectional (pilot) study, was implemented in two different physician populations in Greece from January 2022 to May 2022. The flowchart of the study methods is shown in Figure 1. Physicians from 12 medical specialities (pathologists, general medicine, cardiologists, endocrinologists, oncologists, orthopaedics, rheumatologists, vascular surgeons, pulmonologists, nephrologists, psychiatrists, and neurologists) who manage patients with NCDs participated in both stages. Data collection was conducted online due to the COVID-19 pandemic, and the questionnaires used in this study were converted to Google Forms, an online form-building tool. Informed consent was obtained from all study participants involved in the study.

### 2.2. Instrument and Procedure

#### 2.2.1. Stage 1: The e-Delphi Method-Questionnaire’s Development and Content Validation

In the first stage of the study, an online modified Delphi method (e-Delphi) was conducted with a purposive sample of 16 physician-experts from 12 medical specialities [33,34,35] from January to February 2022. The Delphi method is a systematic, structured, and conciliatory method for determining agreement or consensus among experts and has been widely applied in the health sciences, particularly among physicians [36,37,38]. The e-Delphi method used in the present study aimed to reach consensus among an expert panel on the importance of a series of questions regarding physicians’ KAIAB on PAE, to establish the stability of results, and to assess content validity [39,40,41]. This stage of the study followed the Guidance on Conducting and Reporting Delphi Studies (CREDES) [42] and the Delphi Methodology in Healthcare Research [43].

Before starting the e-Delphi method, the first author conducted a semi-systematic review [44,45] of two electronic databases (PubMed and Scopus) to develop a semi-structured draft questionnaire for round 1, covering the period from 2000 to 2021. To define the sections or scales of the draft questionnaire, a focus group was used [46]. The thematic analysis of the focus group’s qualitative data was conducted to identify, record, describe, categorise, and thematise the data. According to the thematic analysis, the questions generated via the literature review were grouped into the following six scales: i. Demographic, professional, and educational characteristics (35 items), ii. Knowledge (29 items), iii. Attitudes (18 items), iv. Intentions (16 items), v. Abilities (6 items), and vi. Behaviour (22 items) (Questionnaire S1). The focus group consisted of six university professors and researchers with expertise in exercise for health and NCDs. The initial draft questionnaire included 91 closed-ended questions assessing physicians’ KAIAB responses on a 7-point Likert-type scale. Under each closed question, there was also an optional open-ended question for adding comments. Furthermore, after each section, an additional open-ended question was included for experts to submit any questions or suggestions. The questions/items assessing experts’ demographic, professional, and educational characteristics did not require revision by the experts (Figure 1).

At the start of the e-Delphi method, a research team was established to initiate and monitor the e-Delphi process. The first author was the coordinator of the e-Delphi method. Then, the research question of the e-Delphi method was formulated as follows: “What will be the final questions that, with the appropriate algorithm and after achieving consensus and stability, will be included in a final questionnaire assessing physicians’ KAIAB on PAE for NCDs?” The eligibility criteria for the experts’ participation in the e-Delphi method were also defined as follows: (a) have one of the 12 specialities of the study; (b) have more than 10 years of experience in their field; (c) be members of the National Medical Association; (d) have knowledge of issues related to PAE, health and NCDs; (e) be able to devote time to participating in at least two and up to three rounds of the Delphi method of at least 3 months duration; (f) be familiar with the use of a computer and have access to the Internet [43].

The main phase of the e-Delphi method was followed by an invitation to the 16 experts who agreed to participate to complete the draft questionnaire for the quantitative round 1. Before round 1, the research team established the consensus criterion for including each question in the round 2 questionnaire to minimise bias and error [36,42]. The consensus criterion was defined as follows: “Ιn round 2, the included questions on physicians’ KAIAB should have a median value ≥ 5 and achieve a consensus ≥75% [47] for the responses 5, 6, 7 (important questions) on the Likert-type scale (positive consensus). A question should be excluded if its median value was <5 or had a consensus ≥25% for responses 1, 2, 3 (insignificant questions) (negative consensus). Finally, the research team should review questions with a consensus of ≥25% for response 4 (neutral) on the Likert-type scale. The closing criteria for the e-Delphi rounds were also identified a priori by the research team [46] based on consensus and stability scores. A maximum of three rounds was also required [42,47].

At the beginning of round 1, the first author emailed the draft questionnaire to the experts (each identified by a unique code) to obtain their consensus on which questions to include in the semi-structured questionnaire. The experts were asked to indicate the degree of importance of each question in the draft questionnaire on a 7-point Likert-type scale from one to seven (1: Totally unimportant question, 2: Very unimportant question, 3: Quite unimportant question, 4: Neutral question, 5: Quite important question, 6: Very important question, 7: Totally important question) [48]. The experts were also asked to comment on each question, propose revisions, and add any additional questions regarding physicians’ KAIAB on PAE. The experts’ data remained anonymous, confidential, and protected by specialised encryption. The list of these codes was known only to the first author (“quasi-anonymity”) [46].

Following the e-Delphi round 1, a statistical analysis of the quantitative data, a quality assessment of the experts’ comments, and controlled feedback to the experts were implemented. The development of the quantitative round 2 questionnaire (Questionnaire S2) followed. The round 2 questionnaire included closed-ended questions on a 7-point Likert-type scale, open-ended questions at the end of each item, and a comments section. It was emailed to the experts again to obtain consensus, as in round 1. At the end of rounds 1 and 2, the initial draft questionnaire was modified based on consensus criteria, the experts’ comments, and the e-Delphi research group’s meeting outcomes (Questionnaire S3). Following round 2, once consensus and stability were achieved, the content validity ratio (CVR) was used to assess the questionnaire’s content validity. The new questionnaire’s face validity and internal reliability were evaluated in the subsequent stage 2.

*The e-Delphi round 2 (modified) Questionnaire* 

The e-Delphi round 2 (modified) questionnaire was developed at the end of the e-Delphi method, then tested for its content validity, and included 146 question items, grouped into the following six main sections (Questionnaire S3):

1. Demographic, educational, and professional characteristics: Consisted of 44 multiple-choice, open-ended, dichotomous, and Likert-type questions.
2. Knowledge of PAE: Consisted of 29 multiple-choice questions.
3. Attitudes towards PAE: Consisted of 33 Likert scale questions.
4. Intentions towards PAE: Consisted of 8 Likert-type scale questions.
5. Abilities towards PAE: Consisted of 6 Likert-type questions.
6. Behaviour towards PAE. Consisted of a basic Likert-type scale with 9 questions investigating the physicians’ behaviour in PAE counselling, assessment, referral to specialists, and prescription, and of 4 secondary subscales:
  - A subscale with 4 multiple-choice questions to explore the PAE counselling practices (time, mode, content).
  - A subscale with 4 multiple-choice questions to explore the barriers and facilitators of PAE counselling and prescription.
  - A Likert-type subscale with 4 questions to explore the facilitators and barriers to PAE counselling and prescription, respectively.
  - A Likert-type subscale with 5 questions to explore the frequency of referral for exercise to specialists.

At the end of the sixth section, a seventh section with six questions was added to test the questionnaire’s face validity in the subsequent cross-sectional study at stage 2. These questions asked physicians about the time required to complete the questionnaire; the difficulty in understanding the questionnaire’s words, concepts, and phrases; the degree to which questions were clear and served the aim of the questionnaire’s development; the degree the questionnaire included all the necessary and important aspects of the construct being measured; and weather any questions were unnecessary, excessive or incomplete (Questionnaire S3).

#### 2.2.2. Stage 2: The Cross-Sectional (Pilot) Study—Face Validity, Reliability, and Measurements of the Questionnaire

At stage 2 of the study, the new questionnaire’s internal reliability and face validity were evaluated via a subsequent cross-sectional (pilot) study. Cronbach’s alpha was used for each questionnaire scale to assess internal consistency [49]. Physicians’ KAIAB levels and frequencies were additionally measured at stage 2. Stage 2 included a cross-sectional online study (via Google Forms) conducted in the total population of physicians from 12 medical specialities managing patients with NCDs across two Medical Associations (N = 335), from April 2022 to May 2022. A power analysis indicated that a sample size of ≥55 participants would be appropriate (with a power of 0.85, an effect size of 0.50, a two-tailed Type I error rate of α = 0.05, and a Cronbach’s alpha of ≥0.70) to achieve statistical significance [50,51]. To avoid bias, physicians who participated in the e-Delphi method in stage 1 were excluded from stage 2. Participants were included in stage 2 if: (a) they had one of the 12 specialities required by the study, (b) they were members of the National Medical Association, and (c) they worked in the private or public sector. The informed consent form and the online, structured, anonymous, self-administered questionnaire (via Google Forms) were distributed electronically via email to the entire physician population in the two randomly selected prefectures in the country through their respective Medical Associations. This approach was necessary because the Protection of Personal Data Act, reinforced by the Greek Law 4624/2019, prevented access to physicians’ email addresses. Distribution occurred after the main researcher communicated with the two Medical Associations and received their approval. The Google Forms platform was configured to prevent multiple submissions from the same individual, and no personal identifiers were collected. After data collection, responses were exported to Excel and then imported into the statistical software IBM SPSS Statistics version 22.0 (IBM Corp., Armonk, NY, USA) for analysis. All data were stored on a password-protected device accessible only to the research team. Physicians’ KAIAB total scale score (TSS) was computed as the sum of responses to these variables, and KAIAB variables were calculated at the median [52,53]. The three levels of original Bloom’s cut-off points were modified and used to classify physicians’ KAIAB levels in PAE as follows [53,54]: the percentages ≤ 59%, 60–79%, and 80–100% of the KAIAB scale range correspond approximately to low, moderate, and high KAIAB levels, respectively.

### 2.3. Statistical Analysis

IBM SPSS Statistics version 22.0 was used to analyse the data. Since a normality test (Shapiro-Wilk test) showed that the variables were not normally distributed, non-parametric statistical tests were used. Continuous variables were presented as medians and interquartile ranges [55,56], and categorical variables as absolute frequencies (n) and relative frequencies (%). In stage 1, the Wilcoxon signed-rank test was used to assess statistically significant differences in median values between variables from e-Delphi rounds 1 and 2. In stage 2, categorical variables were also expressed as percentages and were compared via the chi-square test. The continuous variables were compared using the Mann-Whitney U test or the Kruskal-Wallis H test. Spearman’s rank correlation coefficient was used to assess the strength and direction of association between paired data. Cronbach’s alpha was used to assess the internal consistency of the KAIAB scales’ items. All tests were two-sided, and *p*-values < 0.05 were considered statistically significant.

## 3. Results

### 3.1. Stage 1: The e-Delphi Method—Questionnaire’s Development and Validation

A total of 20 physician experts were initially invited to the e-Delphi panel, but 16 participated. The response rate in both e-Delphi rounds was 100%. The draft questionnaire’s demographic, professional, and educational characteristics (Table S1) were not required to be revised by the experts. After round 1, based on the experts’ agreement levels (Table S2), eight questions (intention items) were eliminated from the draft questionnaire due to negative consensus. No new questions were added, and some were modified (reworded and/or separated) in response to participants’ comments, without altering their content or meaning (Table S2). The modified questions preceded the letter M (Modified) (Table S2). Table S2 shows experts’ agreement levels at the end of e-Delphi round 1. The questions in Table S2 are presented numerically and appear fully in Questionnaire S1.

After round 2, no KAIAB items were eliminated from the questionnaire; all items were confirmed to meet the consensus criterion (Table S3) and to pass the subsequent stability test (Wilcoxon signed-rank test) (Table S4). The research team also reviewed experts’ comments, but they did not lead to modifications to the questions’ content. The additional questions (attitude and behaviour items) presented in the e-Delphi 2 (modified) questionnaire were not new but already existing ones that were reworded and/or separated based on experts’ comments.Moreover, after round 2, the questionnaire’s content validity was also assessed using the CVR [57] (Table S3). The CVR was calculated by the formula: ne-N/2/N/2, where “ne” was the number of experts who considered the question necessary, and “N” was the total number of experts. The minimum CVR for each question to be included in the final e-Delphi questionnaire, based on the number of experts (N = 16), was 0.49 [58]. In this study, the CVR ranged from 0.5 to 1. There were also seven questions (Table S3) with a low validity ratio (<0.40) that remained in the final e-Delphi questionnaire, based on the initial consensus criterion [consensus ≥25% for response 4 (neutral) on the Likert-type scale]. The experts΄ agreement levels at the end of the e-Delphi round 2 (n = 16) and the CVR are shown in Table S3. The questions in Table S3 are numbered and appear in full in Questionnaires S1 and S2. A Wilcoxon test was also performed on the correlated data [56], and the results for each question item showed no differences between the two e-Delphi rounds, indicating that the responses were stable (Table S4). As the desired consensus and stability in the responses were achieved, a third round was unnecessary [59].

### 3.2. Stage 2. Cross-Sectional (Pilot) Study—Face Validity, and Reliability of the New Questionnaire and Measurements on Physicians’ KAIAB Levels

**Figure 2:**
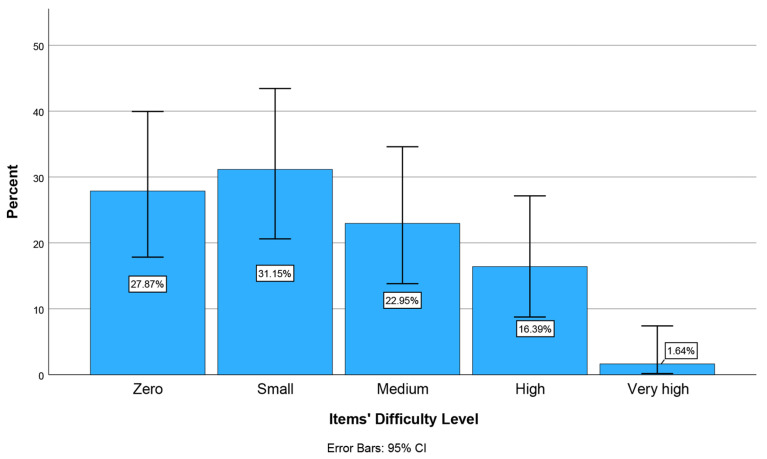
Percentage of physicians across the Items’ Difficulty Level: Degree of difficulty in understanding the questionnaire’s words, concepts, and phrases.

**Figure 3:**
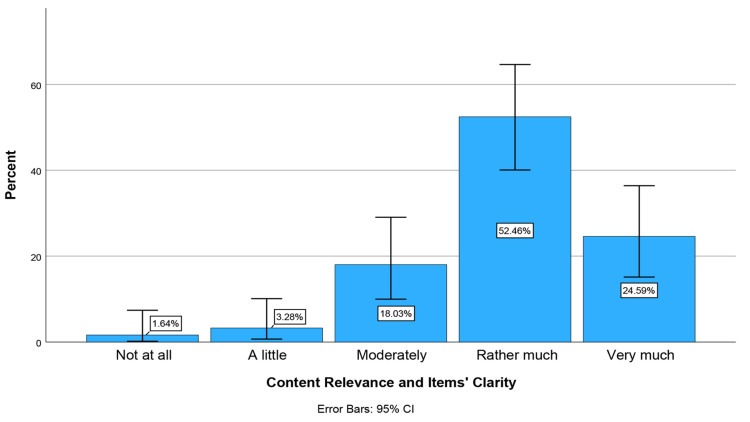
Percentage of physicians across Content Relevance and Items’ Clarity: The degree to which the questions were clear and served the aim of the questionnaire’s development.

**Figure 4:**
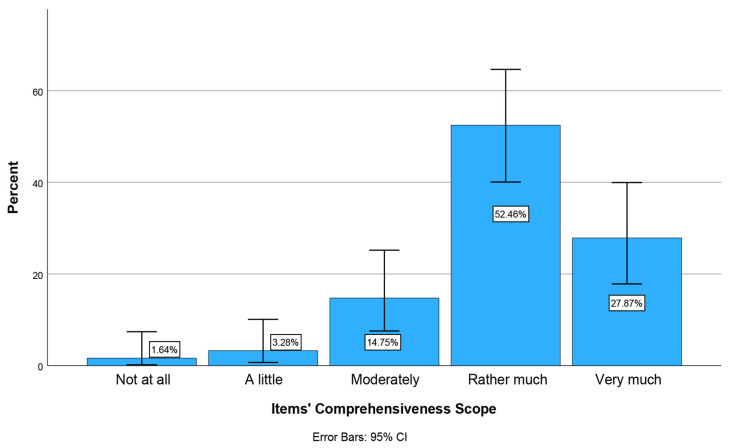
Percentage of physicians across the Items’ Comprehensiveness Scope: The degree to which the questionnaire includes all the necessary, important aspects of the construct being measured (KAIAB on PAE).

At stage 2, all physicians from 12 medical specialities (N = 335) of two Medical Associations were initially invited to participate in the pilot study, but only 61 ultimately participated (response rate 18.2%). The face validity test of the e-Delphi 2 (modified) questionnaire (Questionnaire S3) was conducted using six questions [59]. According to physicians’ responses to these six questions, the majority (>50%) reported that completing each questionnaire took up to 20 min. 82% reported no unnecessary, incomplete, or excessive questions. No comments regarding the questionnaire content were reported. There were only minor comments regarding the reduction in the length of some questions, which were noted. The majority considered the degree of difficulty in understanding the questionnaire’s words, concepts, and phrases to be low (31.1%) and zero (27.9%), respectively (Items’ Difficulty Level) (Figure 2). Physicians also considered the questions to be rather much and very much clear (77.1%) and to serve the aim of the questionnaire’s development (Content Relevance and Items’ Clarity) (Figure 3). Finally, most physicians (80.4%) reported that the questionnaire includes all the necessary and important aspects of the construct being measured (KAIAB on PAE) (Items’ Comprehensiveness Scope) (Figure 4). These results indicate sufficient face validity of the new questionnaire.

Reliability analysis in Table 1 indicates that the items in the questionnaire’s five KAIAB scales had high internal consistency, with Cronbach’s alpha values ranging from 0.805 to 0.931 [49,60]. Three knowledge questions (25, 36, and 44) (Questionnaire S3) were removed at the end of stage 2, as this increased Cronbach’s alpha from 0.805 to 0.833. The Cronbach’s alpha for the subscales of the secondary behaviour variables (perceived barriers and facilitators of exercise counselling, prescription, and referral to specialists) ranged from fair to good (Cronbach’s alpha > 0.7) [60].

**Figure 5:**
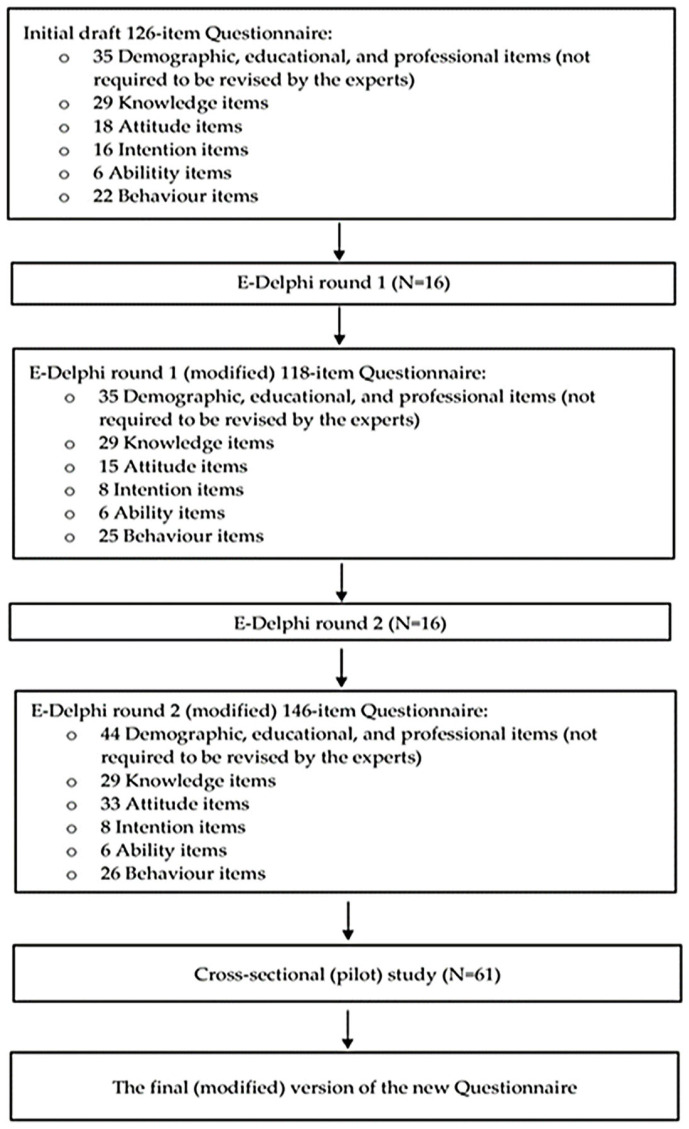
The number of the study’s questionnaire items.

Furthermore, the number of questionnaire items before and after e-Delphi rounds 1 and 2 is presented in Figure 5. 

Preliminary pilot findings regarding physicians’ KAIAB levels in PAE counselling and prescribing are presented in Table 2. The behaviour level was estimated using a basic Likert-type scale (9 items) that assessed physicians’ PAE counselling, assessment, referral to specialists, and prescribing. There was no missing data in the study, as all questions in the KAIAB had to be answered.

At stage 2, correlations between the TSS of physicians’ KAIAB on PAE, their demographic, professional, and educational characteristics, and the absolute and relative frequency distributions of KAIAB variables were also measured (Tables S5, S6, S7, S8, S9 and S10, respectively).

Table 3 presents Spearman’s rank correlation coefficients for the total scale scores of the KAIAB variables at stage 2. According to Spearman’s rank correlation test (ρ), statistically significant moderate-to-strong correlations were found between the variables: (i) attitudes with intentions, abilities, and behaviour; (ii) intentions with abilities and behaviour; and (iii) abilities with behaviour on PAE.

A secondary outcome of stage 2 concerns the barriers to PAE counselling and exercise prescription encountered by physicians. The most common barriers to PAE counselling were lack of time (59%), lack of training in PAE counselling (55.7%), and lack of PAE counselling guidelines and protocols (52.5%). The most common barriers to exercise prescribing were lack of training (77%), lack of continuing education on these topics (62.3%), lack of appropriate exercise prescribing applications (57.4%), and lack of time (50.8%).

## 4. Discussion

Given the lack of data on the needs, perceptions, and practices of physicians in 12 specialities who manage patients with NCDs in promoting PAE, the present study aimed to develop and validate a new questionnaire to assess these physicians’ KAIAB regarding PAE counselling and prescribing. A secondary objective at stage 2 was to assess physicians’ KAIAB levels and frequencies. This two-stage, multimethod observational study is the first in the literature to develop and validate a new questionnaire. The questionnaire was developed and validated using an e-Delphi method and a cross-sectional study. The new instrument was designed to simultaneously assess KAIAB towards PAE counselling and prescription among physicians from 12 specialities who manage patients with NCDs. One objective method for collecting data on people’s knowledge, attitudes, and behaviours is to use questionnaires. Utilising an existing questionnaire can save time and money, but one that assesses the relevant construct may not be readily accessible [61]. Furthermore, questionnaires or surveys are frequently used in medical research to collect quantitative data from patients and healthcare professionals [62].

The main findings of this study were as follows: (i) the development, via the e-Delphi method, of a new questionnaire to effectively assess physicians’ KAIAB across 12 specialities towards PAE on NCDs, following consensus and stability in experts’ responses; (ii) sufficient content and face validity of the new questionnaire; (iii) high internal consistency of the questionnaire’s KAIAB scales; (iv) low, moderate, high, and moderate physicians’ KAIAB levels on PAE; and (v) a statistically significant, moderate-to-strong correlation among the KAIAB variables.

The results at stage 1 of this study demonstrated the effectiveness of the e-Delphi method in developing and content-validating the new research tool [47]. Evidence supports the use of the Delphi method not only to obtain expert consensus for developing a new research tool but also to measure the content validity of the resulting consensus-based instrument [39,40,41]. The questionnaire items for the initial e-Delphi draft were developed and grouped into sections based on an extensive literature review [44,45,63,64] and a subsequent focus group [46], respectively, as is common in Delphi studies [47]. The e-Delphi method of this study resembled the classical Delphi method, characterised by anonymity, repetition, controlled feedback, and statistical analysis of the experts’ group responses [36,38,43,65], but was conducted online (e-Delphi). The advantages of the e-Delphi method [33,35] are well documented. Participants can respond anonymously and without being influenced by others’ opinions. They can also participate at their convenience and from their own space, using an asynchronous process [33,66]. In addition, responses can be collected at different time intervals and from diverse geographical locations. The method is also associated with the lowest possible cost [33,34,35].

The criteria for stopping the e-Delphi rounds of this study, based on consensus, stability, and consensus definition, were defined a priori [43,47,67]. A maximum of three rounds was also required [42,47] to avoid potential expert fatigue and to prevent exit from the method after multiple Delphi rounds, which would reduce the study’s validity by introducing significant errors [64]. Furthermore, after e-Delphi round 2, the content validity of the new questionnaire was assessed by calculating the minimum CVR [45], which ranged from 0.5 to 1; values > 0.49 were considered acceptable [58]. Other researchers have reported that the Delphi method, in addition to achieving consensus, is also used to measure the content validity of their consensus-based research instrument [39,40,41,68]. It is worth noting that throughout stages 1 and 2 of the study, the experts’ personal data remained anonymous and protected using unique codes stored on a computer. Studies report that, through anonymity, experts express themselves freely, without their opinions being influenced by familiarity with other participants [43,65] or by the predominance of the opinions of participants in positions of authority [36,38], thereby reducing group bias [63].

The new questionnaire developed in stage 1 was further tested for face validity and internal consistency (reliability) in stage 2 with a different physician population. Specifically, the face validity control indicated that the questionnaire items were conceptually relevant to the construct they were intended to measure, meaning that the questionnaire sufficiently explored physicians’ KAIAB [57]. Content validity at stage 1 entails a formal assessment by subject-matter experts to determine the appropriateness of the content and to identify any misunderstandings or omissions. Face validity at stage 2 is an informal review of a questionnaire by non-experts who assess its clarity, comprehensibility, and appropriateness for the target group [62,69]. Other studies on physicians’ perceptions of PAE have also been conducted using emailed questionnaires [70], developed through a literature review or an expert panel [38,71,72], and then validated for face and content validity [73]. A study on primary care staff’s knowledge, attitudes, and practices regarding PAE counselling also used a questionnaire, developed following a literature review, that was delivered via email [74]. An additional study on primary care physicians’ attitudes towards PA promotion in primary healthcare settings was conducted via an emailed questionnaire [73]. It was first evaluated via an expert panel and then validated in a regional sample of primary care physicians to control its face validity [73].

Cronbach’s alpha was also used at stage 2 to assess the reliability of the new questionnaire’s five multi-item KAIAB scales, yielding high values (0.805–0.931), indicating high internal consistency. The acceptable range for Cronbach’s alpha is typically 0.70 to 0.95 [62]. It is also worth noting that, since Cronbach’s alpha should be calculated for each concept rather than for the entire questionnaire [75], it was calculated separately for each KAIAB scale. Regarding the behavioural variable, Cronbach’s alpha was calculated for a 5-point Likert-type basic scale covering PAE counselling, assessment, referral to specialists, and prescription. Consistency is a measure of reliability, and Cronbach’s alpha is widely used in education, public health, and the social and behavioural sciences to assess it [62,75,76,77]. The primary use of Cronbach’s coefficient alpha is to estimate the reliability of multi-item scales based on item correlations [78].

KAIAB levels among 61 physicians across 12 specialities toward PAE were also measured as preliminary pilot data in stage 2. The three original Bloom levels’ cut-off points were modified and used to classify physicians’ KAIAB levels in PAE. Bloom’s cut-off points are a common approach in Knowledge, Attitudes, and Practices (KAP) studies in healthcare research for transforming respondents’ scores into three categories (low, moderate, high) [79,80,81,82,83,84]. Nonetheless, such cut-off points are a modified application of Bloom’s taxonomy rather than a universally standardised scoring method. The literature has also proposed alternative thresholds, ranging from 60% to 80% or higher [85], depending on study design and context, with others using thresholds such as 70% as an adequate variable score [83]. Despite this variability, the modified Bloom’s classification remains a useful and widely used method in cross-sectional KAP studies because it enables comparison with other studies and provides a clear way to group multidimensional constructs. In the current study, this methodology was deemed appropriate due to its extensive use and effectiveness in classifying multidimensional constructs such as knowledge, attitudes, and behaviour, while maintaining consistency with previous KAP studies. Specifically, the total scale score of physicians’ knowledge indicates poor knowledge of PAE issues. Although most physicians reported being aware of counselling patients with NCDs in PAE, more than half were not aware of the WHO guidelines on PA and health (Table S6). Moreover, most physicians reported a lack of education on PAE counselling and prescription, and poor perceived knowledge towards exercise prescribing for their patients (Table S5). Similar results have been reported in other studies [86,87]. A study of physicians’ PA knowledge, attitudes, and practices found that confidence and enthusiasm for PA counselling in patients were generally high. In contrast, knowledge of WHO guidelines on PA was low [74]. Another study reported that the majority of physicians were not familiar with the PA national guidelines [88]. Evidence also supports that most physicians stated that they were aware of the benefits of PA, but felt that more information on PA was needed to counsel their patients [73,89].

The total scale scores for attitudes, intentions, and abilities indicate moderate levels of physicians’ sensitisation and self-reported abilities, and high intentions to counsel and promote PAE among their patients (Tables S7–S9). Regarding physicians’ attitudes towards PAE in this study, the majority reported that PAE counselling and health promotion are part of their role and agreed that physically active physicians serve as role models for their patients (Table S7). Another study reported that physically active physicians are PA role models compared with physically inactive [90]. Furthermore, other studies reported that physicians considered PAE counselling for patients with chronic diseases to be the most important health promotion factor [91] and part of their professional role [92]. Similarly, another study [93] found that physicians had good self-confidence in promoting PA, as they perceived it as part of their role. In addition, a study on general practitioners [94] found that physicians reported positive attitudes and perceptions about promoting PA for their patients living with cancer. The moderate level of physicians’ behaviour, as assessed by the basic behaviour scale, indicates moderate PAE promotion practices that require further improvement. Most physicians reported asking, motivating, and counselling their patients in PAE, primarily verbally, but never prescribing exercise (Table S10). Other studies showed similar findings [95,96,97,98,99,100,101,102]. A study among medical oncologists reported that they always, or most of the time, asked patients about their PA and counselled them to increase their PA levels [99]. Fowles et al. reported a low percentage of physicians prescribing exercise [103]. Pojednic et al., in their study of a large sample of sports physicians, also reported that only a small percentage of physicians prescribed exercise, even though the majority recommended PA and discussed exercise with their patients [97]. In exercise prescription, a low proportion was also reported in other studies [104,105,106]. Regarding the most common barriers to PEA counselling and prescribing, physicians in this study cited a lack of time, insufficient training, counselling guidelines and protocols, and limited PEA counselling skills. Previous studies on physicians have found similar barriers [13,26,86,87,95,96,97,100,107].

Another outcome of stage 2 was a statistically significant positive correlation between the variables: (i) attitudes with abilities, intentions, and behaviour on PAE; (ii) intentions with abilities and behaviour on PAE; and iii) abilities with behaviour on PAE. These correlations are consistent with the Theory of Planned Behaviour, which has been used to explain multiple health-related behaviours [108,109]. The model argues that health behaviour is highly dependent on the intention to perform the behaviour, and that attitudes, subjective norms, and perceived behavioural control determine that intention. Attitudes arise from beliefs about the potential consequences of actions and anticipated outcomes [109]. A descriptive study also reported a positive association between physicians’ self-confidence and intentions to promote PA behaviours [104]. Although the Theory of Planned Behaviour was not used to inform the instrument’s conceptual structure, it was applied post hoc in stage 2 to interpret the results.

Almost all studies in the literature assess perceptions of PAE among primary care physicians, family physicians, general practitioners, health care professionals, primary care providers, or physicians in specific specialities. However, no other questionnaire was found in the literature that effectively and simultaneously assesses, via a two-stage, multimethod study, the KAIAB towards PAE among physicians from 12 specialities who manage patients with NCDs. The study provides novel information through a new questionnaire that effectively collects reliable data on physicians’ KAIAB regarding PAE counselling and prescription for NCDs. The e-Delphi method was a useful and effective approach for developing and validating the new questionnaire. Important and determining steps of the e-Delphi method in the study were: the recruitment of the expert group; the a priori definition of consensus; strict adherence to the timeline and all procedures between the two rounds; the anonymity and feedback of the experts; and the definition of specific closing criteria based on ensuring consensus at the end of each round and stability between the two rounds.

The results also highlight the new questionnaire’s sufficient content, face validity, and high reliability. Given the relatively small sample size, neither exploratory nor confirmatory factor analysis was conducted at stage 2. The present study should therefore be considered a preliminary psychometric evaluation of the instrument. Moreover, this study at stage 2 underscores the need to significantly enhance physicians’ PAE counselling, prescribing knowledge, and practices. Since the first step in solving a problem is identifying it, this new questionnaire could be used effectively in large-scale, nationwide studies. It could provide valuable insights and support decision-making regarding physicians’ medical education and training in PAE counselling and prescribing. In addition, it could help guide the development of appropriate PAE counselling guidelines, protocols, and applications for exercise prescription. The ultimate objectives will be to develop intervention programmes and policies for health promotion among patients with chronic diseases, and to integrate PAE into healthcare services as an alternative, non-pharmacological, cost-effective, and co-adjuvant approach to prevent, treat, and manage NCDs and promote public health.

The generalisation of the results obtained in KAIAB in the present study needs to be considered as preliminary pilot data and in relation to the specific structural and organisational characteristics of the Greek healthcare system and the current state of Greek medical practice and education. Although the KAIAB questionnaire demonstrated high reliability and sufficient validity, scale-level scores ranged widely, suggesting that the observed variability may reflect contextual influences rather than measurement limitations. Greece follows a centrally planned mixed healthcare system that is still largely hospital-centred, with low public health expenditure, high out-of-pocket payments, and limited investment in preventive care compared with most European Union countries [110]. Within this context, the low knowledge scores and the limited levels of exercise prescription observed are consistent with evidence of important gaps in formal training in PAE counselling and prescription. Results from Greek medical schools show that a small number of students have sufficient knowledge to prescribe effectively, while the majority feel unprepared and lack education on PAE [111]. These educational and systemic limitations are assumed to influence the low and moderate KAIAB values across all knowledge, behavioural, and implementation domains and support the proposal to strengthen pre- and postgraduate PAE education in Greece. However, caution is warranted when generalising absolute KAIAB domain-level scores to other healthcare systems or cultural settings. Further cross-national and cross-cultural validation studies are required to determine if the observed differences reflect the health system settings or individual physicians.

This study had several limitations. First, although content validity was established through the e-Delphi process involving experts and internal consistency was assessed using Cronbach’s alpha, other forms of validity, such as construct validity, were not examined. It was not feasible to conduct an exploratory factor analysis to reduce the variables to a smaller set of factors, given the small number of participating physicians in the cross-sectional stage 2 study relative to the large number of questionnaire items [112]. Most experts recommend having at least 5 to 10 people per questionnaire item, or at least 100 to 200 participants overall, to obtain reliable findings [113]. When the sample size is small, as in this study, and there are more questionnaire items than participants, studies usually focus on face validity, content validity, and internal consistency using Cronbach’s alpha. A larger sample size will be required to perform advanced statistical analyses, including confirmatory factor analysis. In this study, question grouping into scales was conducted using the focus group method during the design of the initial draft questionnaire, before the e-Delphi method. Although the new questionnaire has sufficient content and face validity and high reliability, it needs to be administered to a larger sample of physicians to further confirm its validity and applicability. It was also not feasible to assess concurrent validity because no validated KAIAB questionnaire for PAE was available among local physicians. Thus, the present study should be considered a preliminary psychometric evaluation of the instrument.

Another limitation is the low response rate among physicians at stage 2 (18.2%). Response rates in epidemiological surveys have declined internationally in recent decades [114,115,116], increasing the likelihood of response bias [117]. Moreover, as this study was conducted during the COVID-19 pandemic, burnout syndrome and increased workload among healthcare workers [118,119] might also have contributed to the low response rate among physicians at stage 2. However, a potential source of non-response bias exists. Because all data were collected anonymously, in accordance with Greek data protection laws (Law 4624/2019, GDPR), it was impossible to systematically compare respondents and non-respondents on demographic or professional characteristics (e.g., medical speciality, years of experience, practice setting). Therefore, the extent of non-response bias and its impact on the study results cannot be empirically estimated. Physicians more interested in PAE and preventive care might have been more likely to respond, leading to an overestimation of KAIAB and suggesting a best-case scenario rather than generalisable, population-level estimates [120,121]. Nevertheless, to minimise the influence of external rewards and respondents’ economic motivations, no financial or other material incentives were offered to physicians for participation [121,122]. Furthermore, Stage 2 was developed as an initial cross-sectional pilot study to assess the face validity and internal consistency of a new questionnaire and findings related to KAIAB levels should be considered preliminary pilot data. Methodological literature indicates that lower response rates may be acceptable for psychometric testing, provided that adequate response variability is achieved [123,124,125]. Future, more extensive validation studies should consider stronger recruitment efforts and, when available, access to auxiliary data to statistically adjust for non-response bias.

One additional limitation is that test-retest reliability was not assessed in this study to determine whether the instrument yields consistent measurements over time. The primary objective was to develop and preliminarily validate the instrument, focusing on content validity (via the Delphi method) and internal consistency (Cronbach’s alpha). Practical constraints related to study design and limited sample availability precluded repeated measurements for evaluating an instrument’s temporal stability. Additionally, data were collected anonymously, without identifying information or self-generated codes. Consequently, matching participants across time points or re-contacting the same individuals for repeated measurement was not possible. This approach was deliberately chosen to protect participant confidentiality and reduce response bias, particularly in self-reported measures. A further limitation concerns the substantial heterogeneity observed in the total scale score of attitudes. While the attitudes score appears moderate, the high interquartile range indicates notable variation in physicians’ responses. This discrepancy could be explained by differing opinions about exercise based on specialities, clinical experience, or levels of training, and therefore must be viewed with caution.

This study is also limited by the potential for social desirability bias, as physicians may have overstated their clinical engagement and knowledge of exercise prescription. Social desirability bias is a well-established limitation in self-reported studies among healthcare professionals, as participants may overreport clinical engagement to conform to professional expectations. Τo address this bias, several established methodological strategies were implemented in this study. First, an anonymous, self-administered online questionnaire was used, eliminating the presence of an interviewer and reducing the normative pressure associated with in-person data collection. Second, questionnaire items were developed using neutral and non-leading language to reduce the risk of acquiescence bias. Third, content validity was established through a rigorous Delphi process with a panel of 16 experts, yielding a Content Validity Ratio (CVR) ranging from 0.5 to 1—an approach specifically recommended to enhance the psychometric rigour of instruments measuring provider behaviour.

A last limitation of this study is that it was conducted in Greece, so the questionnaires were developed and administered in Greek. To accurately present the findings in English, a translation procedure was undertaken, involving a forward translation by one translator and a subsequent back translation by a second. The back-translated version was then compared with the original Greek version to identify any inconsistencies [114,126]. Any minor discrepancies that did not affect the intended meaning were disregarded. Discrepancies that affected meaning or revealed errors were used to refine the final English version. The generalizability of these findings may be limited, as the initial validation was conducted within a specific geographical context, which may restrict their applicability to different healthcare systems unless further cross-cultural adaptation occurs.

## 5. Conclusions

This two-stage observational study addresses the development and validation of a questionnaire to assess physicians’ knowledge, perceptions, and clinical practices regarding the promotion of physical activity and exercise among patients with chronic diseases, a topic of substantial importance to the scientific community. The preliminary pilot data generated in this study may offer initial insights for stakeholders seeking to narrow the gap between scientific evidence and clinical practice, thereby informing future research and implementation in preventive and lifestyle medicine. Such an instrument enables comparability across studies, supports reproducibility of findings, and facilitates both cross-sectional and longitudinal research. Furthermore, the availability of a validated tool supports interdisciplinary research. It provides evidence to inform targeted educational interventions, clinical guidelines, and health policy initiatives to integrate physical activity promotion into healthcare settings. The new questionnaire is recommended for implementation with a larger sample of physicians to further evaluate its reliability and applicability across diverse clinical settings. The results also show that the Greek physicians had high intentions and good practices in PAE counselling, but not in exercise prescription. Moreover, they lacked adequate knowledge of PAE counselling and prescribing. Future nationwide research on the needs, practices, and training related to PAE among physicians managing patients with NCDs is necessary to inform improvements in medical education policies and healthcare system initiatives globally.

## Figures and Tables

**Table 1:** Results of Cronbach’s alpha reliability analysis of the new questionnaire’s KAIAB scales (N = 61).

KAIAB Scales	Type of Questions (Rating)	Number of Questions/Items	Cronbach’s Alpha
Knowledge	Μultiple-choice (Correct answer:1, Wrong answer: 0, Don’t know: 2 or 0)	29	0.805
Attitudes	5-point Likert scale (1–5: Strongly Disagree, Disagree, Neutral, Agree, Strongly Agree)	33	0.931
Intentions	5-point Likert-type scale (1–5: Not at all, A little, Moderately, Rather much, Very much)	8	0.883
Abilities	5-point Likert-type scale (1–5: Not at all, A little, Moderately, Rather much, Very much)	6	0.918
Behaviour	5-point basic Likert-type scale (1–5: Never, Rarely, Sometimes, Often, Always)	9	0.809

**Table 2:** Physicians’ knowledge, attitude, intentions, abilities, and behaviour level on physical activity and exercise (N = 61).

KAIAB Variable Scales	Scale Range/ Level Category	Median Total Scale Score/Interquartile Range	Minimum–Maximum Score Values	KAIAB Level on Physical Activity and Exercise
Knowledge	0–29/ Low (0–17) Moderate (18–23) High (24–29)	13/6	4–23	Low
Attitudes	33–165/ Low (33–111) Moderate (112–138) High (139–165)	128/79	86–165	Moderate
Intentions	8–40/ Low (8–26) Moderate (27–33) High (34–40)	35/9	22–40	High
Abilities	6–30/ Low (6–20) Moderate (21–25) High (26–30)	21/8	6–30	Moderate
Behaviour	9–45/ Low (9–30) Moderate (31–37) High (38–45)	33/8	19–43	Moderate

**Table 3:** Spearman’s rank correlation of total scale score KAIAB variables at stage 2 (Ν = 61).

Total Scale Score Variables	Knowledge	Attitudes	Intentions	Abilities	Behaviour
Knowledge	1				
Attitudes	0.054	1			
Intentions	0.203	0.560 *** (0.000)	1		
Abilities	0.134	0.323 ** (0.011)	0.511 *** (0.000)	1	
Behaviour	0.043	0.450 *** (0.000)	0.663 ** (0.000)	0.612 *** (0.000)	1

** *p* < 0.01, *** *p* < 0.001.

## Data Availability

The data that support the findings of this study are available from the corresponding authors upon reasonable request. The data are not publicly available due to ethical restrictions.

## Supplementary Materials

The following supporting information can be downloaded at https://www.mdpi.com/article/10.3390/healthcare14091148/s1, Questionnaire S1: Initial, draft questionnaire at stage 1 (English translation of the Greek questionnaire); Questionnaire S2: e-Delphi round 1 (modified) questionnaire at stage 1 (English translation of the Greek questionnaire); Questionnaire S3: e-Delphi round 2 (modified) questionnaire at stage 2 (English translation of the Greek questionnaire); Table S1: Demographic professional, and educational characteristics of experts of the e-Delphi method (N = 16) at stage 1; Table S2: Experts’ agreement levels at the end of the e-Delphi round 1 (N = 16) at stage 1; Table S3: Experts’ agreement levels at the end of the e-Delphi round 2 (N = 16) and the content validity ratio (CVR) (N = 16) at stage 1; Table S4: Wilcoxon *p*-value stability test between e-Delphi rounds 1 and 2 (N = 16) at stage 1; Table S5: Demographic professional, and educational characteristics of physicians at stage 2 (N = 61); Table S6: Physicians’ knowledge of physical activity and exercise towards non-communicable diseases at stage 2 (N = 61); Table S7: Physicians’ attitudes on physical activity and exercise towards non-communicable diseases at stage 2 (N = 61); Table S8: Physicians’ intentions on physical activity and exercise towards non-communicable diseases at stage 2 (N = 61); Table S9: Physicians’ abilities on physical activity and exercise towards non-communicable diseases at stage 2 (N = 61); Table S10: Physicians’ behaviour (basic Likert-type scale) on physical activity and exercise towards non-communicable diseases at stage 2 (N = 61).
