# Supplementary material for: Assessing Physicians’ Knowledge, Attitudes, Intentions, Abilities, and Behaviour Toward Physical Activity and Exercise in Non-Communicable Diseases: Questionnaire Development Using an e-Delphi and Cross-Sectional Design
Source: Healthcare (Basel). 2026 Apr 24;14(9):1148. doi: 10.3390/healthcare14091148 (PMC13163534; doi:10.3390/healthcare14091148)
Supplement: Supplementary file 1 [file healthcare-14-01148-s001.zip › healthcare-4236872-supplementary.pdf]

Supplementary Material

Questionnaire S1. Initial draft questionnaire at stage 1 (English translation of the Greek questionnaire).

I. Demographic, professional, and educational characteristics

1. Gender	
Female	☐
Male	☐

2. Age	
20-29	☐
30-39	☐
40-49	☐
50-59	☐
Over 60	☐

3. Family status	
Unmarried	☐
Married	☐
Divorced	☐
Widowed	☐
Cohabitation	☐

4. Do you have children?	
Yes	☐
No	☐

5. In what area have you been living permanently for the last few years?	
City	☐
Town	☐

Village	☐

6. What is your medical speciality? ______________________
7. What is the year you obtained your speciality? ____________
8. How many years have you been working as a physician? ___________

9. At which university did you do your undergraduate studies?	
Greek	☐
International	☐
Other	☐

9a. If you studied at an international or other university, which one?
Please specify __________

10. Do you have any other studies or specialisations (other than your major speciality)?	
Yes	☐
No	☐

10a. If yes, what are they? (Please fill in anything relevant.)	
Bachelor's degree	☐
Master's degree	☐
Doctorate (PhD)	☐
Please specify ______________________	

11. What is your employment status?	
Private medical office	☐
Private Clinic	☐
Public sector (health centre - hospital - fund)	☐

University	☐
Other	☐

12. What region do you work in?	
City	☐
Town	☐
Village	☐

13. Compared to other people your age, would you say that your health is:	
Excellent	☐
Very good	☐
Average	☐
Poor	☐
Very poor	☐

14. Compared to other people your age, would you say that your physical condition/ability is:	
Excellent	☐
Very good	☐
Average	☐
Poor	☐
Very poor	☐

15. Do you smoke?	
Yes	☐
No, never	☐
No, I quit recently	☐
No, I quit at least 1 year ago.	☐

15a. If yes, how many cigarettes per day do you smoke?	
<5	☐
5-10	☐
10-15	☐
15-20	☐
<20	☐

16. Have you ever been taught physical activity and exercise subject(s) at the medical school you attended?	
Yes	☐
No	☐
Don’t remember	☐
16a. If yes, which physical activity and exercise subject(s) have you been taught?<br>Please specify ___________________________	☐

17. Are you informed/educated about physical activity and exercise issues?	
Yes	☐
No	☐

17a.If yes, please indicate the sources from which you are informed/educated about physical activity and exercise (one or more responses)	
- Scientific journals and books	☐
- Scientific bodies/exercise bodies	☐
- Authoritative medical internet resources (internet)	☐
- Media	☐
- Seminars/conferences	☐
-Lifelong learning programs	☐
- Other	☐

17b. If you have already participated in a seminar or lifelong learning program in physical activity and exercise, how many hours of training have you participated in?	
<1	☐
1-3	☐

4-10	☐
11-40	☐
>40	☐
Don’t remember	☐

18. Do you wish to participate in a seminar or training program on physical activity and exercise in the future?	
Yes	☐
No	☐

18a. If yes, what kind of training would you like to participate in?	
- Synchronous distance	☐
- Asynchronous distance	☐
-In-person	☐
- Hybrid (distance and in-person)	☐
- Other	☐

18b. If yes, how many hours would you like to be trained in a physical activity and exercise training seminar or program?	
<1	☐
1-3	☐
4-10	☐
11-40	☐
>40	☐

19. Do you have knowledge of physical activity and exercise issues?	
Yes	☐
No	☐

19a.If yes, what is your knowledge level?	Very low	Low	Medium	Good	Very good
	☐	☐	☐	☐	☐

20. Do you have knowledge of counselling patients with chronic non-communicable diseases on physical activity and exercise?	
Yes	☐
No	☐

20a. If yes, what is your knowledge level?	Very low	Low	Medium	Good	Very good
	☐	☐	☐	☐	☐

21. Do you have knowledge of prescribing physical activity and exercise for patients with chronic non-communicable diseases?	
Yes	☐
No	☐

21a.If yes, what is your knowledge level?	Very low	Low	Medium	Good	Very good
	☐	☐	☐	☐	☐

22. Do you know about assessing physical activity and exercise levels in patients with chronic non-communicable diseases?	
Yes	☐
No	☐

22a. If yes, what is your knowledge level?	Very low	Low	Medium	Good	Very good
	☐	☐	☐	☐	☐

23 . Do you know the World Health Organisation (WHO) guidelines on physical activity for health?	
Yes	☐
No	☐

II. Knowledge of physical activity and exercise

Question	Degree of importance						
	Totally unimportant	Very unimportant	Quite unimportant	Neutral	Quite important	Very important	Totally important
24. Physical activity is:<br>-The involvement in sports<br>-Walking<br>-Exercise<br>-Any physical movement<br>- Don't know	1 ☐	2 ☐	3 ☐	4 ☐	5 ☐	6 ☐	7 ☐
Comment/rational							
25. Exercise is:<br>- Engaging in sports<br>- Walking<br>- It is synonymous with physical activity<br>- Any physical movement<br>- A structured and planned program of physical activity aimed at improving physical fitness<br>- Don't know	1 ☐	2 ☐	3 ☐	4 ☐	5 ☐	6 ☐	7 ☐
Comment/rationale:							
26. When we say "means" of exercise, we mean:<br>- The intensity of the exercise<br>- The frequency of the exercise<br>- The instrument of the exercise<br>- The duration of the exercise<br>- The intensity of the exercise,<br>-The type of exercise (aerobic, resistance, etc.)<br>- Don't know	1 ☐	2 ☐	3 ☐	4 ☐	5 ☐	6 ☐	7 ☐
Comment/rationale:							
27. The intensity of the exercise is:<br>- How many minutes do we exercise<br>- How many times a week do we exercise<br>- The difficulty of the exercise<br>- The exercise equipment<br>- Don't know	1 ☐	2 ☐	3 ☐	4 ☐	5 ☐	6 ☐	7 ☐
Comment/rationale:							

	1	2	3	4	5	6	7
28. The duration of the exercise is:<br>- How many minutes do we exercise?<br>- How many times a week do we exercise<br>- The difficulty of the exercise<br>- The exercise equipment<br>- Don't know	☐	☐	☐	☐	☐	☐	☐
Comment/rationale:							
29. The frequency of exercise is:<br>- How many minutes do we exercise<br>- How many times a week do we exercise?<br>- The difficulty of the exercise<br>- The instrument of the exercise<br>- How many times a month do we exercise<br>- Don't know	1 ☐	2 ☐	3 ☐	4 ☐	5 ☐	6 ☐	7 ☐
Comment/rationale:							
30. Which of the following is an indicator of exercise intensity?<br>- Heart rate<br>- The training duration<br>- The subjective feeling of fatigue<br>- The kilometres per hour (speed)<br>- How many times a month do we exercise<br>- How many steps do we take<br>- Don't know<br>(One or more responses)	1 ☐	2 ☐	3 ☐	4 ☐	5 ☐	6 ☐	7 ☐
Comment/rationale:							
31. Which of the following is a resistance training exercise?<br>- Running on the treadmill<br>- Aerobic programme<br>- Weight training<br>- Abdominal exercises<br>- Push-ups<br>- Throws with medicine balls<br>- Stretches<br>- Don't know<br>(One or more responses)	1 ☐	2 ☐	3 ☐	4 ☐	5 ☐	6 ☐	7 ☐
Comment/rationale:							

32. Which of the following is aerobic exercise?<br>- Walking<br>- Gardening<br>- Cycling<br>- Weight training in the gym<br>- Single rope pulls<br>- 100 m sprint<br>- Swimming<br>- Don't know<br>(One or more responses)	1 ☐	2 ☐	3 ☐	4 ☐	5 ☐	6 ☐	7 ☐
Comment/rationale:							
33. Which of the following is a mobility exercise?<br>- Running on the treadmill<br>- Stretching<br>- Balance exercises<br>- Pilates<br>- Yoga<br>- Exercises with weights<br>- Don't know<br>(One or more responses)	1 ☐	2 ☐	3 ☐	4 ☐	5 ☐	6 ☐	7 ☐
Comment/rationale:							
34. Which of the following is a high-intensity interval exercise?<br>- Pilates<br>- Yoga<br>- 5 sets of jumps with a break in between<br>- 10 sprints with a break in between<br>- 5 sets of one weight training exercise with a break in between<br>- 2 runs per day (morning-evening) with an 8-hour break in between<br>- 3 training sessions per week with a day's rest in between<br>- Don't know<br>(One or more responses)	1 ☐	2 ☐	3 ☐	4 ☐	5 ☐	6 ☐	7 ☐
Comment/rationale:							
35. “PAR-Q and YOU” is:<br>- Type of treadmill<br>- Type of bicycle<br>- Type of aerobic training<br>- Type of resistance training	1 ☐	2 ☐	3 ☐	4 ☐	5 ☐	6 ☐	7 ☐

- Training suitability questionnaire<br>- Questionnaire on predisposing factors for cardiovascular disease<br>- Don't know							
Comment/rationale:							
36. "MET" is:<br>- Type of dumbbell<br>- Type of stretches<br>- Unit of measurement of exercise intensity<br>- Type of resistance<br>- Aerobic programme<br>- Oxygen consumption<br>- Don't know<br>(One or more responses)	1<br>☐	2<br>☐	3<br>☐	4<br>☐	5<br>☐	6<br>☐	7<br>☐
Comment/rationale:							
37. The risk of a non-fatal heart attack during vigorous exercise in asymptomatic individuals is:<br>- 1 episode per approximately 10 million hours of training<br>- 1 event per approximately 5 million hours of training<br>- 1 episode per approximately 1 million hours of training<br>- 1 episode per approximately 500.000 hours of training<br>- 1 episode per approximately 100. 000 hours of training<br>- 1 episode per approximately 50.000 hours of training<br>- 1 episode per approximately 10. 000 hours of training<br>- Don't know	1<br>☐	2<br>☐	3<br>☐	4<br>☐	5<br>☐	6<br>☐	7<br>☐
Comment/rationale:							
38. The risk of a fatal heart attack during vigorous exercise for non-symptomatic individuals is:<br>- 1 episode per approximately 9,5 million hours of training<br>- 1 episode per approximately 4,3 million hours of training<br>- 1 episode per approximately 1, 6 million hours of training<br>- 1 episode per approximately 900.000 hours of training	1<br>☐	2<br>☐	3<br>☐	4<br>☐	5<br>☐	6<br>☐	7<br>☐

- 1 episode per approximately 385. 000 hours of training<br>- 1 episode per approximately 75.000 hours of training<br>- 1 episode per approximately 12. 300 hours of training<br>- Don't know							
Comment/rationale:							
39. The risk of a heart attack during intense supervised training for cardiac patients is:<br>- 1 episode per approximately 7,2 million hours of training<br>- 1 episode per approximately 3,6 million hours of training<br>- 1 episode per approximately 1,1 million hours of training<br>- 1 episode per approximately 650.000 hours of training<br>- 1 episode per approximately 195.000 hours of training<br>- 1 episode per approximately 58.000 hours of training<br>- 1 episode per approximately 9.600 hours of training<br>- Don't know	1<br>☐	2<br>☐	3<br>☐	4<br>☐	5<br>☐	6<br>☐	7<br>☐
Comment/rationale:							
40. The "Maxium Oxygen Uptake"(VO2max) refers to:<br>- Oxygen consumption circulated under exhaustion conditions during aerobic exercise<br>- Oxygen consumption circulated under exhaustion conditions during muscle strengthening exercise<br>- Oxygen consumption circulated at rest<br>- Oxygen consumption when we start to get tired<br>- Don't know	1<br>☐	2<br>☐	3<br>☐	4<br>☐	5<br>☐	6<br>☐	7<br>☐
Comment/rationale:							
41. "One-Repetition Maximum" (1RM) is:<br>- The maximum number of push-ups I can perform with correct technique	1<br>☐	2<br>☐	3<br>☐	4<br>☐	5<br>☐	6<br>☐	7<br>☐

- The maximum number of abdominals I can perform with correct technique<br>- The maximum number of weights I can lift with correct technique in a weight training exercise<br>- One repetition when performing weight training exercises that has a maximum duration<br>- Don't know							
Comment/rationale:							
42. Stretching exercises are:<br>- Those that increase the range of motion of a joint<br>- Those that increase the length of a muscle<br>- The resistance exercises<br>- The balance exercises<br>- Don't know<br>(One or more answers)	1 ☐	2 ☐	3 ☐	4 ☐	5 ☐	6 ☐	7 ☐
Comment/rationale:							
43. According to the World Health Organisation's Global Action Plan on Physical Activity 2018-2030, the goal is to reduce global physical inactivity by:<br>5%<br>10%<br>15%<br>20%<br>25%<br>- Don't know	1 ☐	2 ☐	3 ☐	4 ☐	5 ☐	6 ☐	7 ☐
Comment/rationale:							
44. According to the World Health Organisation, what is the minimum number of days per week of moderate-intensity physical activity that adults should do to achieve health benefits?<br>1<br>2<br>3<br>4<br>5	1 ☐	2 ☐	3 ☐	4 ☐	5 ☐	6 ☐	7 ☐

6<br>- Don't know							
Comment/rationale:							
45. According to the World Health Organisation, what is the minimum number of minutes of moderate-intensity aerobic physical activity recommended per week for adults and people with chronic NCDs (hypertension, type 2 diabetes and cancer) to achieve health benefits?<br>50<br>100<br>150<br>200<br>250<br>300<br>- Don't know	1<br>☐	2<br>☐	3<br>☐	4<br>☐	5<br>☐	6<br>☐	7<br>☐
Comment/rationale:							
46. According to the World Health Organisation, what is the minimum number of minutes of vigorous-intensity aerobic physical activity recommended per week for adults and people with chronic NCDs (hypertension, type 2 diabetes and cancer) to achieve health benefits?<br>50<br>75<br>100<br>125<br>150<br>175<br>- Don't know	1<br>☐	2<br>☐	3<br>☐	4<br>☐	5<br>☐	6<br>☐	7<br>☐
Comment/rationale:							
47. According to the World Health Organisation, what is the minimum number of workouts per week for muscle strengthening?<br>1<br>2<br>3	1<br>☐	2<br>☐	3<br>☐	4<br>☐	5<br>☐	6<br>☐	7<br>☐

4<br>5<br>6<br>- Don't know							
Comment/rationale:							
48. Regular physical activity can benefit cancer patients as follows:<br>- Improving fatality<br>- Improving the risk of recurrence<br>- Improving morbidity<br>- All the above<br>- Don't know	1 ☐	2 ☐	3 ☐	4 ☐	5 ☐	6 ☐	7 ☐
Comment/rationale:							
49. Regular physical activity can benefit patients with type 2 diabetes as follows:<br>- Reducing mortality from cardiovascular disease<br>- Improving mortality<br>- Reducing the progression of the disease<br>- All the above<br>- Don't know	1 ☐	2 ☐	3 ☐	4 ☐	5 ☐	6 ☐	7 ☐
Comment/rationale:							
50. How is physical inactivity defined?<br>- Physical activity lasting less than 10 minutes daily<br>- Physical activity lasting less than 20 minutes daily<br>- Physical activity lasting less than 30 minutes daily<br>- Physical activity lasting less than 40 minutes per day<br>- Physical activity of less than 60 minutes per day<br>- Don't know	1 ☐	2 ☐	3 ☐	4 ☐	5 ☐	6 ☐	7 ☐
Comment/rationale:							
51. How can the physical activity and exercise level of a patient with a chronic non-communicable disease be assessed?<br>- By means of heart rate monitors<br>- By physical activity questionnaires	1 ☐	2 ☐	3 ☐	4 ☐	5 ☐	6 ☐	7 ☐

- By accelerometers<br>- By pedometers<br>- By mobile phones<br>- Don't know<br>(One or more responses)							
Comment/rationale:							
52. Which of the following research tools (questionnaires) are you familiar with for assessing physical activity levels?<br>-International Physical Activity Questionnaire (IPAQ)<br>- Global Physical Activity Questionnaire (GPAQ)<br>- 7-day Physical Activity Recall Questionnaire (7-day PAR Q)<br>-Other<br>-None of the above<br>- Don't know<br>(One or more responses)	1 ☐	2 ☐	3 ☐	4 ☐	5 ☐	6 ☐	7 ☐
Comment/rationale:							
Please add any questions or suggestions you think should be included in the final questionnaire to investigate physicians' knowledge of physical activity and exercise adequately.							
In addition to the above-mentioned questions, please record any questions or suggestions you consider important to include in the final questionnaire to investigate physicians' knowledge of physical activity and exercise adequately.							

III. Attitudes towards physical activity and exercise

**Question**	**Degree of importance**						
	**Totally unimportant**	**Very unimportant**	**Quite unimportant**	**Neutral**	**Quite important**	**Very important**	**Totally important**
53. Physical activity and exercise are important for health	1 ☐	2 ☐	3 ☐	4 ☐	5 ☐	6 ☐	7 ☐
Comment/rationale:							
54. Physical activity and exercise are necessary for health	1 ☐	2 ☐	3 ☐	4 ☐	5 ☐	6 ☐	7 ☐
Comment/rationale:							

55. It is important for physicians to ask their patients if they exercise or perform physical activity	1 ☐	2 ☐	3 ☐	4 ☐	5 ☐	6 ☐	7 ☐
Comment/rationale:							
56. It is important for physicians to advise their patients about the benefits of physical activity and exercise	1 ☐	2 ☐	3 ☐	4 ☐	5 ☐	6 ☐	7 ☐
Comment/rationale:							
57. It is important for physicians to prescribe exercise to their patients	1 ☐	2 ☐	3 ☐	4 ☐	5 ☐	6 ☐	7 ☐
Comment/rationale:							
58. It is important for physicians to assess whether there is an exercise-related change in the health of their patients who exercise	1 ☐	2 ☐	3 ☐	4 ☐	5 ☐	6 ☐	7 ☐
Comment/rationale:							
59. It is important for physicians to assess their patients' level of physical activity or fitness	1 ☐	2 ☐	3 ☐	4 ☐	5 ☐	6 ☐	7 ☐
Comment/rationale:							
60. Health promotion through physical activity and exercise is one of the physicians' duties	1 ☐	2 ☐	3 ☐	4 ☐	5 ☐	6 ☐	7 ☐
Comment/rationale:							
61. It is essential that a course on physical activity and exercise be taught in the undergraduate curriculum of medical students	1 ☐	2 ☐	3 ☐	4 ☐	5 ☐	6 ☐	7 ☐
Comment/rationale:							
62. It is essential that a course on physical activity and exercise be taught in the undergraduate curriculum of all Health Sciences students	1 ☐	2 ☐	3 ☐	4 ☐	5 ☐	6 ☐	7 ☐
Comment/rationale:							
63. Patients change their behaviour after exercise counselling	1 ☐	2 ☐	3 ☐	4 ☐	5 ☐	6 ☐	7 ☐
Comment/rationale:							

64. Physicians should be physically active to act as a role model for their patients	1 ☐	2 ☐	3 ☐	4 ☐	5 ☐	6 ☐	7 ☐
Comment/rationale:							
65. Counselling, design, and implementation of exercise programmes for people with chronic NCDs is a duty of a physician	1 ☐	2 ☐	3 ☐	4 ☐	5 ☐	6 ☐	7 ☐
Comment/rationale:							
66. Counselling, design, and implementation of exercise programmes for people with chronic NCDs is a duty of a physical education graduate	1 ☐	2 ☐	3 ☐	4 ☐	5 ☐	6 ☐	7 ☐
Comment/rationale:							
67. Counselling, design, and implementation of exercise programmes for people with chronic NCDs is a duty of a qualified nurse	1 ☐	2 ☐	3 ☐	4 ☐	5 ☐	6 ☐	7 ☐
Comment/rationale:							
68. Counselling, design, and implementation of exercise programmes for people with chronic NCDs is a duty of a dietician	1 ☐	2 ☐	3 ☐	4 ☐	5 ☐	6 ☐	7 ☐
Comment/rationale:							
69. Counselling, design, and implementation of exercise programmes for people with chronic NCDs is a duty of a physiotherapist	1 ☐	2 ☐	3 ☐	4 ☐	5 ☐	6 ☐	7 ☐
Comment/rationale:							
70. Counselling, design, and implementation of exercise programmes for people with chronic NCDs is a duty of a psychologist	1 ☐	2 ☐	3 ☐	4 ☐	5 ☐	6 ☐	7 ☐
Comment/rationale:							
In addition to the above-mentioned questions, please record any questions or suggestions you consider important to include in the final questionnaire to investigate physicians' attitudes towards physical activity and exercise adequately.							

IV. Intentions on physical activity and exercise

Question	Degree of importance						
	Totally unimportant	Very unimportant	Quite unimportant	Neutral	Quite important	Very important	Totally important
71. I intend to ask my patients if they perform physical activity or exercise	1 ☐	2 ☐	3 ☐	4 ☐	5 ☐	6 ☐	7 ☐
Comment/rationale:							
72. I intend to assess the physical activity levels of my patients with non-communicable diseases	1 ☐	2 ☐	3 ☐	4 ☐	5 ☐	6 ☐	7 ☐
Comment/rationale:							
73. I intend to advise my patients on physical activity and exercise	1 ☐	2 ☐	3 ☐	4 ☐	5 ☐	6 ☐	7 ☐
Comment/rationale:							
74. I intend to assess the effect of physical activity and exercise on my patients' health	1 ☐	2 ☐	3 ☐	4 ☐	5 ☐	6 ☐	7 ☐
Comment/rationale:							
75. I would like to prescribe exercise to my patients with non-communicable diseases	1 ☐	2 ☐	3 ☐	4 ☐	5 ☐	6 ☐	7 ☐
Comment/rationale:							
76. I intend to follow the exercise programs of my patients with chronic non-communicable diseases	1 ☐	2 ☐	3 ☐	4 ☐	5 ☐	6 ☐	7 ☐
Comment/rationale:							
77. I intend to motivate my patients with chronic non-communicable diseases to exercise	1 ☐	2 ☐	3 ☐	4 ☐	5 ☐	6 ☐	7 ☐
Comment/rationale:							
78. I don't exercise, and I don't intend to	1 ☐	2 ☐	3 ☐	4 ☐	5 ☐	6 ☐	7 ☐
Comment/rationale:							
79. I don't exercise, but I intend to do so	1 ☐	2 ☐	3 ☐	4 ☐	5 ☐	6 ☐	7 ☐
Comment/rationale:							

80. I intend to take up exercise, but I don't know how	1 ☐	2 ☐	3 ☐	4 ☐	5 ☐	6 ☐	7 ☐
Comment/rationale:							
81. I exercise occasionally, but I intend to stop completely	1 ☐	2 ☐	3 ☐	4 ☐	5 ☐	6 ☐	7 ☐
Comment/rationale:							
82. I exercise occasionally, but I plan to exercise more regularly	1 ☐	2 ☐	3 ☐	4 ☐	5 ☐	6 ☐	7 ☐
Comment/rationale:							
83. I exercise regularly, and I intend to continue to do so	1 ☐	2 ☐	3 ☐	4 ☐	5 ☐	6 ☐	7 ☐
Comment/rationale:							
84. I encounter barriers to participating in physical activity and exercise programs	1 ☐	2 ☐	3 ☐	4 ☐	5 ☐	6 ☐	7 ☐
Comment/rationale:							
84a. The barriers I encounter to participating in physical activity and exercise programmes are:<br>-Lack of time<br>-Lack of support from spouse/partner<br>-Lack of information about the benefits and ways of exercising<br>-The inability to find a trainer to guide me<br>-My bad physical condition<br>-The existence of health problems (illness/injury)<br>-Obesity<br>-I am embarrassed to be in the same exercise area as fitter people<br>-Fear of injury<br>-My reduced interest in exercise<br>-I prefer to take care of my health through medication rather than exercise<br>-Other	1 ☐	2 ☐	3 ☐	4 ☐	5 ☐	6 ☐	7 ☐

Comment/rationale:							
85. I intend to be educated in physical activity and exercise	1 ☐	2 ☐	3 ☐	4 ☐	5 ☐	6 ☐	7 ☐
Comment/rationale:							
In addition to the above-mentioned questions, please record any questions or suggestions you consider important to include in the final questionnaire to investigate physicians' intentions regarding physical activity and exercise adequately.							

V. Abilities on physical activity and exercise

**Question**	**Degree of importance**						
	**Totally unimportant**	**Very unimportant**	**Quite unimportant**	**Neutral**	**Quite important**	**Very important**	**Totally important**
86. I feel able to counsel my patients on physical activity and exercise	1 ☐	2 ☐	3 ☐	4 ☐	5 ☐	6 ☐	7 ☐
Comment/rationale:							
87. I feel able to prescribe exercise to my patients with chronic non-communicable diseases	1 ☐	2 ☐	3 ☐	4 ☐	5 ☐	6 ☐	7 ☐
Comment/rationale:							
88. I feel able to assess physical activity levels in my patients with chronic non-communicable diseases	1 ☐	2 ☐	3 ☐	4 ☐	5 ☐	6 ☐	7 ☐
Comment/rationale:							
89. I feel able to evaluate the impact of physical activity and exercise on the health of my patients with chronic non-communicable diseases after my consultation	1 ☐	2 ☐	3 ☐	4 ☐	5 ☐	6 ☐	7 ☐
Comment/rationale:							
90. I feel able to follow the exercise programs of my patients with chronic non-communicable diseases	1 ☐	2 ☐	3 ☐	4 ☐	5 ☐	6 ☐	7 ☐
Comment/rationale:							

91. I feel able to motivate my patients with chronic non-communicable diseases to exercise	1 ☐	2 ☐	3 ☐	4 ☐	5 ☐	6 ☐	7 ☐
Comment/rationale:							
In addition to the above-mentioned questions, please record any questions or suggestions you consider important to include in the final questionnaire to investigate physicians' abilities regarding physical activity and exercise adequately.							

VI. Behaviour on physical activity and exercise

Question	Degree of importance						
	**Totally unimportant**	**Very unimportant**	**Quite unimportant**	**Neutral**	**Quite important**	**Very important**	**Totally important**
92. I ask my patients with chronic non-communicable diseases if they exercise or perform physical activity	1 ☐	2 ☐	3 ☐	4 ☐	5 ☐	6 ☐	7 ☐
Comment/rationale:							
93. I assess physical activity levels in my patients with chronic non-communicable diseases	1 ☐	2 ☐	3 ☐	4 ☐	5 ☐	6 ☐	7 ☐
Comment/rationale:							
94. I advise my patients about physical activity and exercise and their health benefits	1 ☐	2 ☐	3 ☐	4 ☐	5 ☐	6 ☐	7 ☐
Comment/rationale:							
94a. Regarding my counselling, I report:<br>i. Time of consultation per visit:<br><10 minutes<br>10-30 minutes<br>30-60 minutes<br>>60 minutes<br>ii. Consultation method<br>-Verbal<br>-Written<br>iii. Content of advice<br>- Health benefits of physical activity and exercise	1 ☐	2 ☐	3 ☐	4 ☐	5 ☐	6 ☐	7 ☐

-Type of activity/exercise<br>- Intensity of exercise<br>-Duration of exercise<br>-Frequency of exercise<br>iv. My comfort level with exercise counselling<br>-Very low<br>-Low<br>-Medium<br>-High<br>-Very high							
Comment/rationale:							
94b. I counsel on physical activity and exercise the patients with the following chronic non-communicable diseases (one or more responses)<br>- Obesity<br>- Hypertension<br>- Diabetes<br>- Lipid problems or metabolic syndrome<br>- Kidney diseases<br>- Cardiovascular diseases<br>- Respiratory diseases<br>- Cancer<br>- Arthritis<br>- Osteoporosis<br>- Neurological problems<br>- Psychiatric problems<br>- Other	1	2	3	4	5	6	7
Comment/rationale:							
94c. If I counsel, evaluate the effect of physical activity and exercise on the health of my patients after my consultation	1	2	3	4	5	6	7
Comment/rationale:							
94d.If I advise, I positively influence my patients in terms of physical activity and exercise with my counselling	1	2	3	4	5	6	7
Comment/rationale:							
95. There are factors that facilitate exercise counselling	1	2	3	4	5	6	7

Comment/rationale:							
95a. Factors that facilitate exercise counselling are:<br>-Adequacy of time<br>-Training on physical activity and exercise counselling skills<br>-Prior knowledge of physical activity and exercise counselling<br>-The interest of my patients<br>-Financial compensation<br>-Other	1<br>☐	2<br>☐	3<br>☐	4<br>☐	5<br>☐	6<br>☐	7<br>☐
Comment/rationale:							
96. There are barriers to exercise counselling	1<br>☐	2<br>☐	3<br>☐	4<br>☐	5<br>☐	6<br>☐	7<br>☐
Comment/rationale:							
96a. Barriers I encounter in exercise counselling:<br>- Lack of my own time<br>- Lack of patient time<br>- Lack of counselling skills<br>- Lack of training<br>- Lack of financial motivation<br>- Reduced interest of patients in changing their lifestyle<br>- Patients' preference for a drug rather than exercise<br>- Preference to advise on other healthy lifestyle issues rather than exercise<br>- Lack of exercise counselling guidelines/protocols for doctors<br>- Other	1<br>☐	2<br>☐	3<br>☐	4<br>☐	5<br>☐	6<br>☐	7<br>☐
Comment/rationale:							
97. There are factors that could facilitate the prescription of exercise in patients with chronic non-communicable diseases	1<br>☐	2<br>☐	3<br>☐	4<br>☐	5<br>☐	6<br>☐	7<br>☐
Comment/rationale:							
97a. Factors that would facilitate the prescription of exercise are:	1<br>☐	2<br>☐	3<br>☐	4<br>☐	5<br>☐	6<br>☐	7<br>☐

-Excess time of physicians<br>-Excess time of patients<br>-Training of physicians<br>-Availability of appropriate applications for physicians<br>-Continuous training of physicians<br>-Existence of a financial incentive<br>-Increased interest of patients<br>-Adoption of appropriate legislation<br>-Other							
Comment/rationale:							
98. There are barriers in prescribing exercise to patients with chronic non-communicable diseases	1<br>☐	2<br>☐	3<br>☐	4<br>☐	5<br>☐	6<br>☐	7<br>☐
Comment/rationale:							
98a. Barriers I encounter in prescribing exercise:<br>- Lack of physicians' time<br>- Lack of patients' time<br>- Lack of training<br>- Lack of applications for physicians<br>-Lack of continuing education<br>- Lack of financial incentive<br>- Decreased interest in my patients<br>- Patients' preference for medication rather than exercise.<br>- The lack of appropriate legislation<br>- Other	1<br>☐	2<br>☐	3<br>☐	4<br>☐	5<br>☐	6<br>☐	7<br>☐
Comment/rationale:							
99. I follow the exercise programs of my patients with chronic non-communicable diseases	1<br>☐	2<br>☐	3<br>☐	4<br>☐	5<br>☐	6<br>☐	7<br>☐
Comment/rationale:							
100. I refer my patients with chronic non-communicable diseases for counselling, planning, and implementation	1<br>☐	2<br>☐	3<br>☐	4<br>☐	5<br>☐	6<br>☐	7<br>☐

of health exercise programs to a physical education graduate							
Comment/rationale:							
101. I refer my patients with chronic non-communicable diseases for counselling, planning, and implementation of health exercise programs to a qualified nurse	1 ☐	2 ☐	3 ☐	4 ☐	5 ☐	6 ☐	7 ☐
Comment/rationale:							
102. I refer my patients with chronic non-communicable diseases for counselling, planning, and implementation of health exercise programs to a dietitian	1 ☐	2 ☐	3 ☐	4 ☐	5 ☐	6 ☐	7 ☐
Comment/rationale:							
103. I refer my patients with chronic non-communicable diseases for counselling, planning, and implementation of health exercise programs to a physiotherapist	1 ☐	2 ☐	3 ☐	4 ☐	5 ☐	6 ☐	7 ☐
Comment/rationale:							
104. I refer my patients with chronic non-communicable diseases for counselling, planning, and implementation of health exercise programs to a psychologist	1 ☐	2 ☐	3 ☐	4 ☐	5 ☐	6 ☐	7 ☐
Comment/rationale:							
105. I motivate my patients with chronic non-communicable diseases to exercise	1 ☐	2 ☐	3 ☐	4 ☐	5 ☐	6 ☐	7 ☐
Comment/rationale:							
In addition to the above-mentioned questions, please record any questions or suggestions you consider important to include in the final questionnaire to investigate physicians' behaviour regarding physical activity and exercise adequately.							

Questionnaire S2. E-Delphi round 1 (modified) questionnaire at stage 1(English translation of the Greek questionnaire).

I. Demographic, professional, and educational characteristics

1. Gender	
Female	☐
Male	☐

2. Age	
20-29	☐
30-39	☐
40-49	☐
50-59	☐
Over 60	☐

3. Family status	
Unmarried	☐
Married	☐
Divorced	☐
Widowed	☐
Cohabitation	☐

4. Do you have children?	
Yes	☐
No	☐

5. In what area have you been living permanently for the last few years?	
City	☐
Town	☐
Village	☐

6. What is your medical speciality? ______________________
7. What is the year you obtained your speciality? ___________
8. How many years have you been working as a physician? ___________

9. At which university did you do your undergraduate studies?	
Greek	
International	
Other	

9a. If you studied at an international or other university, which one?
Please specify __________

10. Do you have any other studies or specialisations (other than your major speciality)?	
Yes	
No	

10a. If yes, what are they? (Please fill in anything relevant.)	
Bachelor's degree	
Master's degree	
Doctorate (PhD)	
Please specify ______________________	

11. What is your employment status?	
Private medical office	
Private Clinic	
Public sector (health centre - hospital - fund)	

University	☐
Other	☐

12. What region do you work in?	
City	☐
Town	☐
Village	☐

13. Compared to other people your age, would you say that your health is:	
Excellent	☐
Very good	☐
Average	☐
Poor	☐
Very poor	☐

14. Compared to other people your age, would you say that your physical condition/ability is:	
Excellent	☐
Very good	☐
Average	☐
Poor	☐
Very poor	☐

15. Do you smoke?	
Yes	☐
No, never	☐
No, I quit recently	☐
No, I quit at least 1 year ago.	☐

15a. If yes, how many cigarettes per day do you smoke?	
<5	
5-10	
10-15	
15-20	
<20	

16. Have you ever been taught physical activity and exercise subjects at the medical school you attended?	
Yes	
No	
Don’t remember	
16a. If yes, which physical activity and exercise subject(s) have you been taught? Please specify ___________________________	

17. Are you informed/educated about physical activity and exercise issues?	
Yes	
No	

17a.If yes, please indicate the sources from which you are informed/educated about physical activity and exercise (one or more responses)	
- Scientific journals and books	
- Scientific bodies/exercise bodies	
- Authoritative medical internet resources (internet)	
- Media	
- Seminars/conferences	
-Lifelong learning programs	
- Other	

17b. If you have already participated in a seminar or lifelong learning program in physical activity and exercise, how many hours of training have you participated in?	
<1	
1-3	
4-10	

11-40	☐
>40	☐
Don’t remember	☐

18. Do you wish to participate in a seminar or training program on physical activity and exercise in the future?	
Yes	☐
No	☐

18a. If yes, what kind of training would you like to participate in?	
- Synchronous distance	☐
- Asynchronous distance	☐
-In-person	☐
- Hybrid (distance and in-person)	☐
- Other	☐

18b. If yes, how many hours would you like to be trained in a physical activity and exercise training seminar or program?	
<1	☐
1-3	☐
4-10	☐
11-40	☐
>40	☐

19. Do you have knowledge of physical activity and exercise?	
Yes	☐
No	☐

19a.If yes, what is your knowledge level?	Very low	Low	Medium	Good	Very good
	☐	☐	☐	☐	☐

20. Do you have knowledge of counselling patients with chronic non-communicable diseases on physical activity and exercise?	
Yes	☐
No	☐

20a. If yes, what is your knowledge level?	Very low	Low	Medium	Good	Very good
	☐	☐	☐	☐	☐

21. Do you have knowledge of prescribing physical activity and exercise for patients with chronic non communicable diseases?	
Yes	☐
No	☐

21a.If yes, what is your knowledge level?	Very low	Low	Medium	Good	Very good
	☐	☐	☐	☐	☐

22. Do you have knowledge in assessing physical activity and exercise levels in patients with chronic non-communicable diseases?	
Yes	☐
No	☐

22a. If yes, what is your knowledge level?	Very low	Low	Medium	Good	Very good
	☐	☐	☐	☐	☐

23. Do you know the World Health Organisation (WHO) guidelines on physical activity for health?	
Yes	☐
No	☐

II. Knowledge of physical activity and exercise

Question	Degree of importance						
	**Totally unimportant**	**Very unimportant**	**Quite unimportant**	**Neutral**	**Quite important**	**Very important**	**Totally important**
24. Physical activity is:<br>-The involvement in sports<br>-Walking<br>-Exercise<br>-Any physical movement<br>- Don't know	1 ☐	2 ☐	3 ☐	4 ☐	5 ☐	6 ☐	7 ☐
Comment/rational							
25.Exercise is:<br>- Engaging in sports<br>- Walking<br>- It is synonymous with physical activity<br>- Any physical movement<br>- A structured and planned program of physical activity aimed at improving physical fitness<br>- Don't know	1 ☐	2 ☐	3 ☐	4 ☐	5 ☐	6 ☐	7 ☐
Comment/rationale:							
M26. When we say, " major principles of exercise program design", we mean:<br>- The intensity of the exercise<br>- The frequency of the exercise<br>- The instrument of the exercise<br>- The duration of the exercise<br>- The intensity of the exercise,<br>- The type of exercise (e.g., aerobic, resistance, etc.)<br>- Don't know	1 ☐	2 ☐	3 ☐	4 ☐	5 ☐	6 ☐	7 ☐
Comment/rationale:							
27.The intensity of the exercise is:<br>- How many minutes do we exercise<br>- How many times a week do we exercise<br>- The difficulty of the exercise<br>- The exercise equipment<br>- Don't know	1 ☐	2 ☐	3 ☐	4 ☐	5 ☐	6 ☐	7 ☐

Comment/rationale:							
28. The duration of the exercise is:<br>- How many minutes do we exercise?<br>- How many times a week do we exercise<br>- The difficulty of the exercise<br>- The exercise equipment<br>- Don't know	1 ☐	2 ☐	3 ☐	4 ☐	5 ☐	6 ☐	7 ☐
Comment/rationale:							
29. The frequency of exercise is:<br>- How many minutes do we exercise<br>- How many times a week do we exercise?<br>- The difficulty of the exercise<br>- The instrument of the exercise<br>- How many times a month do we exercise<br>- Don't know	1 ☐	2 ☐	3 ☐	4 ☐	5 ☐	6 ☐	7 ☐
Comment/rationale:							
30. Which of the following is an indicator of exercise intensity?<br>- Heart rate<br>- The training duration<br>- The subjective feeling of fatigue<br>- The kilometres per hour (speed)<br>- How many times a month do we exercise<br>- How many steps we take<br>- Don't know<br>(One or more responses)	1 ☐	2 ☐	3 ☐	4 ☐	5 ☐	6 ☐	7 ☐
Comment/rationale:							
31. Which of the following is a resistance training exercise?<br>- Running on the treadmill<br>- Aerobic programme<br>- Weight training<br>- Abdominal exercises<br>- Push-ups<br>- Throws with medicine balls<br>- Stretches<br>- Don't know<br>(One or more responses)	1 ☐	2 ☐	3 ☐	4 ☐	5 ☐	6 ☐	7 ☐
Comment/rationale:							

32. Which of the following is aerobic exercise?<br>- Walking<br>- Gardening<br>- Cycling<br>- Weight training in the gym<br>- Single rope pulls<br>- 100 m sprint<br>- Swimming<br>- Don't know<br>(One or more responses)	1<br>☐	2<br>☐	3<br>☐	4<br>☐	5<br>☐	6<br>☐	7<br>☐
Comment/rationale:							
33. Which of the following is a mobility exercise?<br>- Running on the treadmill<br>- Stretching<br>- Balance exercises<br>- Pilates<br>- Yoga<br>- Exercises with weights<br>- Don't know<br>(One or more responses)	1<br>☐	2<br>☐	3<br>☐	4<br>☐	5<br>☐	6<br>☐	7<br>☐
Comment/rationale:							
34. Which of the following is a high-intensity interval exercise?<br>- Pilates<br>- Yoga<br>- 5 sets of jumps with a break in between<br>- 10 sprints with a break in between<br>- 5 sets of one weight training exercise with a break in between<br>- 2 runs per day (morning-evening) with an 8-hour break in between<br>- 3 training sessions per week with a day's rest in between<br>- Don't know<br>(One or more responses)	1<br>☐	2<br>☐	3<br>☐	4<br>☐	5<br>☐	6<br>☐	7<br>☐
Comment/rationale:							
35. “PAR-Q and YOU” is:<br>- Type of treadmill<br>- Type of bicycle<br>- Type of aerobic training<br>- Type of resistance training	1<br>☐	2<br>☐	3<br>☐	4<br>☐	5<br>☐	6<br>☐	7<br>☐

- Training suitability questionnaire<br>- Questionnaire on predisposing factors for cardiovascular disease<br>- Don't know							
Comment/rationale:							
36. “MET” is:<br>- Type of dumbbell<br>- Type of stretches<br>- Unit of measurement of exercise intensity<br>- Type of resistance<br>- Aerobic programme<br>- Oxygen consumption<br>- Don't know<br>(One or more responses)	1 ☐	2 ☐	3 ☐	4 ☐	5 ☐	6 ☐	7 ☐
Comment/rationale:							
37. The risk of a non-fatal heart attack during vigorous exercise in asymptomatic individuals is:<br>- 1 episode per approximately 10 million hours of training<br>- 1 event per approximately 5 million hours of training<br>- 1 episode per approximately 1 million hours of training<br>- 1 episode per approximately 500.000 hours of training<br>- 1 episode per approximately 100. 000 hours of training<br>- 1 episode per approximately 50.000 hours of training<br>- 1 episode per approximately 10. 000 hours of training<br>- Don't know	1 ☐	2 ☐	3 ☐	4 ☐	5 ☐	6 ☐	7 ☐
Comment/rationale:							
38. The risk of a fatal heart attack during vigorous exercise for non-symptomatic individuals is:<br>- 1 episode per approximately 9,5 million hours of training<br>- 1 episode per approximately 4,3 million hours of training<br>- 1 episode per approximately 1, 6 million hours of training<br>- 1 episode per approximately 900.000 hours of training	1 ☐	2 ☐	3 ☐	4 ☐	5 ☐	6 ☐	7 ☐

- 1 episode per approximately 385. 000 hours of training<br>- 1 episode per approximately 75.000 hours of training<br>- 1 episode per approximately 12. 300 hours of training<br>- Don't know							
Comment/rationale:							
39. The risk of a heart attack during intense supervised training for cardiac patients is:<br>- 1 episode per approximately 7,2 million hours of training<br>- 1 episode per approximately 3, 6 million hours of training<br>- 1 episode per approximately 1,1 million hours of training<br>- 1 episode per approximately 650.000 hours of training<br>- 1 episode per approximately 195.000 hours of training<br>- 1 episode per approximately 58.000 hours of training<br>- 1 episode per approximately 9.600 hours of training<br>- Don't know	1<br>☐	2<br>☐	3<br>☐	4<br>☐	5<br>☐	6<br>☐	7<br>☐
Comment/rationale:							
40. The "Maxium Oxygen Uptake"(VO2max) refers to:<br>- Oxygen consumption circulated under exhaustion conditions during aerobic exercise<br>- Oxygen consumption circulated under exhaustion conditions during muscle strengthening exercise<br>- Oxygen consumption circulated at rest<br>- Oxygen consumption when we start to get tired<br>- Don't know	1<br>☐	2<br>☐	3<br>☐	4<br>☐	5<br>☐	6<br>☐	7<br>☐
Comment/rationale:							
41. "One-Repetition Maximum" (1RM) is:<br>- The maximum number of push-ups I can perform with correct technique	1<br>☐	2<br>☐	3<br>☐	4<br>☐	5<br>☐	6<br>☐	7<br>☐

- The maximum number of abdominals I can perform with correct technique<br>- The maximum number of weights I can lift with correct technique in a weight training exercise<br>- One repetition when performing weight training exercises that has a maximum duration<br>- Don't know							
Comment/rationale:							
42. Stretching exercises are:<br>- Those that increase the range of motion of a joint<br>- Those that increase the length of a muscle<br>- The resistance exercises<br>- The balance exercises<br>- Don't know<br>(One or more answers)	1 ☐	2 ☐	3 ☐	4 ☐	5 ☐	6 ☐	7 ☐
Comment/rationale:							
43. According to the World Health Organisation's Global Action Plan on Physical Activity 2018-2030, the goal is to reduce global physical inactivity by:<br>5%<br>10%<br>15%<br>20%<br>25%<br>- Don't know	1 ☐	2 ☐	3 ☐	4 ☐	5 ☐	6 ☐	7 ☐
Comment/rationale:							
44. According to the World Health Organisation, what is the minimum number of days per week of moderate-intensity physical activity that adults should do to achieve health benefits?<br>1<br>2<br>3<br>4<br>5	1 ☐	2 ☐	3 ☐	4 ☐	5 ☐	6 ☐	7 ☐

6<br>- Don't know							
Comment/rationale:							
45. According to the World Health Organisation, what is the minimum number of minutes of moderate-intensity aerobic physical activity recommended per week for adults and people with chronic NCDs (hypertension, type 2 diabetes and cancer) to achieve health benefits?<br>50<br>100<br>150<br>200<br>250<br>300<br>- Don't know	1 ☐	2 ☐	3 ☐	4 ☐	5 ☐	6 ☐	7 ☐
Comment/rationale:							
46. According to the World Health Organisation, what is the minimum number of minutes of vigorous-intensity aerobic physical activity recommended per week for adults and people with chronic NCDs (hypertension, type 2 diabetes and cancer) to achieve health benefits?<br>50<br>75<br>100<br>125<br>150<br>175<br>- Don't know	1 ☐	2 ☐	3 ☐	4 ☐	5 ☐	6 ☐	7 ☐
Comment/rationale:							
47. According to the World Health Organisation, what is the minimum number of workouts per week for muscle strengthening?<br>1<br>2<br>3	1 ☐	2 ☐	3 ☐	4 ☐	5 ☐	6 ☐	7 ☐

4<br>5<br>6<br>- Don't know							
Comment/rationale:							
48. Regular physical activity can benefit cancer patients as follows:<br>- Improving fatality<br>- Improving the risk of recurrence<br>- Improving morbidity<br>- All the above<br>- Don't know	1<br>☐	2<br>☐	3<br>☐	4<br>☐	5<br>☐	6<br>☐	7<br>☐
Comment/rationale:							
49. Regular physical activity can benefit patients with type 2 diabetes as follows:<br>- Reducing mortality from cardiovascular disease<br>- Improving mortality<br>- Reducing the progression of the disease<br>- All the above<br>- Don't know	1<br>☐	2<br>☐	3<br>☐	4<br>☐	5<br>☐	6<br>☐	7<br>☐
Comment/rationale:							
50. How is physical inactivity defined?<br>- Physical activity lasting less than 10 minutes daily<br>- Physical activity lasting less than 20 minutes daily<br>- Physical activity lasting less than 30 minutes daily<br>- Physical activity lasting less than 40 minutes per day<br>- Physical activity of less than 60 minutes per day<br>- Don't know	1<br>☐	2<br>☐	3<br>☐	4<br>☐	5<br>☐	6<br>☐	7<br>☐
Comment/rationale:							
51. How can the physical activity and exercise level of a patient with a chronic non-communicable disease be assessed?<br>- Using heart rate monitors<br>- By physical activity questionnaires	1<br>☐	2<br>☐	3<br>☐	4<br>☐	5<br>☐	6<br>☐	7<br>☐

- By accelerometers<br>- By pedometers<br>- By mobile phones<br>- Don't know<br>(One or more responses)							
Comment/rationale:							
52. Which of the following research tools (questionnaires) are you familiar with for assessing physical activity levels?<br>-International Physical Activity Questionnaire (IPAQ)<br>- Global Physical Activity Questionnaire (GPAQ)<br>- 7-day Physical Activity Recall Questionnaire (7-day PAR Q)<br>-Other<br>-None of the above<br>- Don't know<br>(One or more responses)	1<br>☐	2<br>☐	3<br>☐	4<br>☐	5<br>☐	6<br>☐	7<br>☐
Comment/rationale:							
Please add your questions or suggestions that you think need to be included in the final questionnaire, to adequately investigate physicians' knowledge of physical activity and exercise.							
In addition to the above-mentioned questions, please record your questions or suggestions that you consider important to add to the final questionnaire, to adequately investigate physicians' knowledge of physical activity and exercise.							

III. Attitudes towards physical activity and exercise

Question	Degree of importance						
	**Totally unimportant**	**Very unimportant**	**Quite unimportant**	**Neutral**	**Quite important**	**Very important**	**Totally important**
53. Physical activity and exercise are important for health	1 ☐	2 ☐	3 ☐	4 ☐	5 ☐	6 ☐	7 ☐
Comment/rationale:							
54. Physical activity and exercise are necessary for health	1 ☐	2 ☐	3 ☐	4 ☐	5 ☐	6 ☐	7 ☐
Comment/rationale:							
55. It is important for physicians to ask their patients if they exercise or perform physical activity	1 ☐	2 ☐	3 ☐	4 ☐	5 ☐	6 ☐	7 ☐
Comment/rationale:							
56. It is important for physicians to advise their patients about the benefits of physical activity and exercise	1 ☐	2 ☐	3 ☐	4 ☐	5 ☐	6 ☐	7 ☐
Comment/rationale:							
M57. It is important for physicians to prescribe exercise to their patients	1 ☐	2 ☐	3 ☐	4 ☐	5 ☐	6 ☐	7 ☐
Comment/rationale:							
58. It is important for physicians to assess whether there is an exercise-related change in the health of their patients who exercise	1 ☐	2 ☐	3 ☐	4 ☐	5 ☐	6 ☐	7 ☐
Comment/rationale:							
M59. It is important for physicians to know how to assess their patients' level of physical activity or fitness	1 ☐	2 ☐	3 ☐	4 ☐	5 ☐	6 ☐	7 ☐
Comment/rationale:							
60. Health promotion through physical activity and exercise is one of physicians’ duties	1 ☐	2 ☐	3 ☐	4 ☐	5 ☐	6 ☐	7 ☐
Comment/rationale:							
61. It is essential that a course on physical activity and exercise be	1 ☐	2 ☐	3 ☐	4 ☐	5 ☐	6 ☐	7 ☐

taught in the undergraduate curriculum of medical students							
Comment/rationale:							
62. It is essential that a course on physical activity and exercise be taught in the undergraduate curriculum of all Health Sciences students	1 ☐	2 ☐	3 ☐	4 ☐	5 ☐	6 ☐	7 ☐
Comment/rationale:							
63. Patients change their behaviour after exercise counselling	1 ☐	2 ☐	3 ☐	4 ☐	5 ☐	6 ☐	7 ☐
Comment/rationale:							
M64. Physically active physicians act as a positive role model for their patients	1 ☐	2 ☐	3 ☐	4 ☐	5 ☐	6 ☐	7 ☐
Comment/rationale:							
M65.Exercise counselling for persons with chronic non-communicable diseases is a duty of:<br>a) a physician managing patients with chronic non-communicable diseases<br>b) a graduate in physical education<br>c) a nurse managing patients with chronic non-communicable diseases<br>d) a dietician,<br>e) a physiotherapist<br>f) a clinical psychologist	1 ☐	2 ☐	3 ☐	4 ☐	5 ☐	6 ☐	7 ☐
Comment/rationale:							
M66. The design of exercise programs for people with chronic non-communicable diseases is a duty of:<br>a) a physician managing patients with chronic non-communicable diseases<br>b) a graduate in physical education	1 ☐	2 ☐	3 ☐	4 ☐	5 ☐	6 ☐	7 ☐

c) a nurse managing patients with chronic non-communicable diseases<br>d) a dietician,<br>e) a physiotherapist<br>f) a clinical psychologist							
Comment/rationale:							
M67. The implementation of exercise programs for people with chronic, non-communicable diseases is a duty of:<br>a) a physician managing patients with chronic non-communicable diseases<br>b) a graduate in physical education<br>c) a nurse managing patients with chronic non-communicable diseases<br>d) a dietician,<br>e) a physiotherapist<br>f) a clinical psychologist	1 ☐	2 ☐	3 ☐	4 ☐	5 ☐	6 ☐	7 ☐
Comment/rationale:							
In addition to the above-mentioned questions, please record your questions or suggestions that you consider important to add to the final questionnaire, to adequately investigate physicians' attitudes towards physical activity and exercise.							

IV. Intentions on physical activity and exercise

Question	Degree of importance						
	**Totally unimportant**	**Very unimportant**	**Quite unimportant**	**Neutral**	**Quite important**	**Very important**	**Totally important**
M71. I intend to ask or continue to ask my patients if they perform physical activity or exercise	1 ☐	2 ☐	3 ☐	4 ☐	5 ☐	6 ☐	7 ☐
Comment/rationale:							
M72. I intend to assess or continue to assess the physical activity levels of my patients with chronic non-communicable diseases	1 ☐	2 ☐	3 ☐	4 ☐	5 ☐	6 ☐	7 ☐
Comment/rationale:							
M73. I intend to counsel or continue to counsel on physical activity and exercise my patients with chronic non-communicable diseases	1 ☐	2 ☐	3 ☐	4 ☐	5 ☐	6 ☐	7 ☐
Comment/rationale:							
M74. I intend to evaluate or continue to evaluate the impact of physical activity and exercise on the health of my patients	1 ☐	2 ☐	3 ☐	4 ☐	5 ☐	6 ☐	7 ☐
Comment/rationale:							
75. I would like to prescribe exercise to my patients with non-communicable diseases	1 ☐	2 ☐	3 ☐	4 ☐	5 ☐	6 ☐	7 ☐
Comment/rationale:							
M76. I intend to follow or continue to follow the progress of the exercise programs of my patients with chronic non-communicable diseases	1 ☐	2 ☐	3 ☐	4 ☐	5 ☐	6 ☐	7 ☐
Comment/rationale:							
M77. I intend to motivate or continue to motivate my patients with chronic non-communicable diseases to exercise	1 ☐	2 ☐	3 ☐	4 ☐	5 ☐	6 ☐	7 ☐
Comment/rationale:							
M85.I intend to be actively educated on counselling patients with chronic non-	1 ☐	2 ☐	3 ☐	4 ☐	5 ☐	6 ☐	7 ☐

communicable diseases on physical activity and exercise							
Comment/rationale:							
In addition to the above-mentioned questions, please record your questions or suggestions that you consider important to add to the final questionnaire, to adequately investigate physicians' intentions on physical activity and exercise.							

V. Abilities on physical activity and exercise

Question	Degree of importance						
	**Totally unimportant**	**Very unimportant**	**Quite unimportant**	**Neutral**	**Quite important**	**Very important**	**Totally important**
86. I feel able to counsel my patients on physical activity and exercise	1 ☐	2 ☐	3 ☐	4 ☐	5 ☐	6 ☐	7 ☐
Comment/rationale:							
87. I feel able to prescribe exercise to my patients with chronic non-communicable diseases	1 ☐	2 ☐	3 ☐	4 ☐	5 ☐	6 ☐	7 ☐
Comment/rationale:							
88. I feel able to assess physical activity levels in my patients with chronic non-communicable diseases	1 ☐	2 ☐	3 ☐	4 ☐	5 ☐	6 ☐	7 ☐
Comment/rationale:							
89. I feel able to evaluate the impact of physical activity and exercise on the health of my patients with chronic non-communicable diseases after my consultation	1 ☐	2 ☐	3 ☐	4 ☐	5 ☐	6 ☐	7 ☐
Comment/rationale:							
M90. I feel able to follow the progress of the exercise programs of my patients with chronic non-communicable diseases	1 ☐	2 ☐	3 ☐	4 ☐	5 ☐	6 ☐	7 ☐
Comment/rationale:							

91. I feel able to motivate my patients with chronic non-communicable diseases to exercise	1 ☐	2 ☐	3 ☐	4 ☐	5 ☐	6 ☐	7 ☐
Comment/rationale:							
In addition to the above-mentioned questions, please record your questions or suggestions that you consider important to add to the final questionnaire, to adequately investigate physicians' abilities on physical activity and exercise.							

VI. Behaviour on physical activity and exercise

**Question**	**Degree of importance**						
	**Totally unimportant**	**Very unimportant**	**Quite unimportant**	**Neutral**	**Quite important**	**Very important**	**Totally important**
92. I ask my patients with chronic non-communicable diseases if they exercise or perform physical activity	1 ☐	2 ☐	3 ☐	4 ☐	5 ☐	6 ☐	7 ☐
Comment/rationale:							
93. I assess physical activity levels in my patients with chronic non-communicable diseases	1 ☐	2 ☐	3 ☐	4 ☐	5 ☐	6 ☐	7 ☐
Comment/rationale:							
94. I advise my patients about physical activity and exercise and their health benefits	1 ☐	2 ☐	3 ☐	4 ☐	5 ☐	6 ☐	7 ☐
Comment/rationale:							
M94a. My average consultation time per visit is:<br><5 minutes<br>5-10 minutes<br>30-60 minutes<br>>60 minutes	1 ☐	2 ☐	3 ☐	4 ☐	5 ☐	6 ☐	7 ☐
Comment/rationale:							
M94b. My usual consultation method is:<br>-Verbal<br>-Written	1 ☐	2 ☐	3 ☐	4 ☐	5 ☐	6 ☐	7 ☐

Comment/rationale:							
M94c. The content of my counselling is (one or more responses):<br>- Health benefits of physical activity and exercise<br>-Type of activity/exercise<br>- Intensity of exercise<br>-Duration of exercise<br>-Frequency of exercise	1 ☐	2 ☐	3 ☐	4 ☐	5 ☐	6 ☐	7 ☐
Comment/rationale:							
M94d. I counsel my patients about the following chronic, non-communicable diseases (one or more responses):<br>- Obesity<br>- Hypertension<br>- Diabetes<br>- Lipid problems or metabolic syndrome<br>- Kidney diseases<br>- Cardiovascular diseases<br>- Respiratory diseases<br>- Cancer<br>- Arthritis<br>- Autoimmune diseases<br>- Osteoporosis<br>- Neurological problems<br>- Psychiatric problems<br>- Other	1 ☐	2 ☐	3 ☐	4 ☐	5 ☐	6 ☐	7 ☐
Comment/rationale:							
M94e. I evaluate the effect of physical activity and exercise on the health of my patients after my consultation	1 ☐	2 ☐	3 ☐	4 ☐	5 ☐	6 ☐	7 ☐
Comment/rationale:							
M94f. I positively influence my patients in terms of physical activity and exercise with my counselling	1 ☐	2 ☐	3 ☐	4 ☐	5 ☐	6 ☐	7 ☐
Comment/rationale:							
95. There are factors that facilitate exercise counselling	1 ☐	2 ☐	3 ☐	4 ☐	5 ☐	6 ☐	7 ☐

Comment/rationale:							
95a. Factors that facilitate exercise counselling are:<br>-Adequacy of time<br>-Training on physical activity and exercise counselling skills<br>-Prior knowledge of physical activity and exercise counselling<br>-The interest of my patients<br>-Financial compensation<br>-Other	1<br>☐	2<br>☐	3<br>☐	4<br>☐	5<br>☐	6<br>☐	7<br>☐
Comment/rationale:							
M96. There are barriers (or potential barriers) to exercise counselling	1<br>☐	2<br>☐	3<br>☐	4<br>☐	5<br>☐	6<br>☐	7<br>☐
Comment/rationale:							
96a. Barriers I encounter in exercise counselling:<br>- Lack of my own time<br>- Lack of patient time<br>- Lack of counselling skills<br>- Lack of training<br>- Lack of financial motivation<br>- Reduced interest of patients in changing their lifestyle<br>- Patients' preference for a drug rather than exercise<br>- Preference to advise on other healthy lifestyle issues rather than exercise<br>- Lack of exercise counselling guidelines/protocols for doctors<br>- Other	1<br>☐	2<br>☐	3<br>☐	4<br>☐	5<br>☐	6<br>☐	7<br>☐
Comment/rationale:							
97. There are factors that could facilitate the prescription of exercise in patients with chronic non-communicable diseases	1<br>☐	2<br>☐	3<br>☐	4<br>☐	5<br>☐	6<br>☐	7<br>☐
Comment/rationale:							
97a. Factors that would facilitate the prescription of exercise are:<br>-Excess time of physicians	1<br>☐	2<br>☐	3<br>☐	4<br>☐	5<br>☐	6<br>☐	7<br>☐

-Excess time of patients<br>-Training of physicians<br>-Availability of appropriate applications for physicians<br>-Continuous training of physicians<br>-Existence of a financial incentive<br>-Increased interest of patients<br>-Adoption of appropriate legislation<br>-Other							
Comment/rationale:							
M98. There are barriers (or potential barriers) in prescribing exercise to patients with chronic non-communicable diseases	1 ☐	2 ☐	3 ☐	4 ☐	5 ☐	6 ☐	7 ☐
Comment/rationale:							
98a. Barriers I encounter in prescribing exercise:<br>- Lack of physicians' time<br>- Lack of patients' time<br>- Lack of training<br>- Lack of applications for physicians<br>-Lack of continuing education<br>- Lack of financial incentive<br>- Decreased interest of my patients<br>- Patients' preference for medication rather than exercise.<br>- The lack of appropriate legislation<br>- Other	1 ☐	2 ☐	3 ☐	4 ☐	5 ☐	6 ☐	7 ☐
Comment/rationale:							
M99. I follow the progress of exercise programs for my patients with chronic, non-communicable disease	1 ☐	2 ☐	3 ☐	4 ☐	5 ☐	6 ☐	7 ☐
Comment/rationale:							
M100. I refer my patients with chronic non-communicable diseases for counselling, planning, and implementation	1 ☐	2 ☐	3 ☐	4 ☐	5 ☐	6 ☐	7 ☐

of health exercise programs to other specialists.							
Comment/rationale:							
M100a. If yes, I refer them to a physical education graduate	1 ☐	2 ☐	3 ☐	4 ☐	5 ☐	6 ☐	7 ☐
Comment/rationale:							
M100b. If yes, I refer them to a registered nurse	1 ☐	2 ☐	3 ☐	4 ☐	5 ☐	6 ☐	7 ☐
Comment/rationale:							
M100c. If yes, I refer them to a dietitian	1 ☐	2 ☐	3 ☐	4 ☐	5 ☐	6 ☐	7 ☐
Comment/rationale:							
M100d. If yes, I refer them to a physiotherapist	1 ☐	2 ☐	3 ☐	4 ☐	5 ☐	6 ☐	7 ☐
Comment/rationale:							
M100e. If yes, I refer them to a psychologist	1 ☐	2 ☐	3 ☐	4 ☐	5 ☐	6 ☐	7 ☐
Comment/rationale:							
105. I motivate my patients with chronic non-communicable diseases to exercise	1 ☐	2 ☐	3 ☐	4 ☐	5 ☐	6 ☐	7 ☐
Comment/rationale:							
In addition to the above-mentioned questions, please record your questions or suggestions that you consider important to add to the final questionnaire, to adequately investigate physicians' behaviour on physical activity and exercise.							

Notes:

1. 8 questions (78, 79, 80, 81, 82, 83, 84, 84a), which were included in the e-Delphi round 1 questionnaire were removed from the 2nd questionnaire, due to the negative consensus of the experts.
2. The e-Delphi round 2 questionnaire did not include new questions for expert review, but existing questions that were reworded/clarified, compressed and/or split by the research team following the experts' comments.
3. In the e-Delphi round 2 questionnaire, 23 questions were reworded and/or split, yielding 30 in the e-Delphi round 2 compared to round 1, and were preceded by the letter M (Modified). Eight of these questions were not new in terms of their content, but existing questions that were split by another question.
4. As some questions from the Delphi round 1 were removed and some others were modified without changing their content, the numbering of the questions in the questionnaire of the e-Delphi round 2 is not continuous. This was to enable a subsequent comparison of the questions between the two e-Delphi rounds.

Table S1. Demographic professional, and educational characteristics of experts of the e-Delphi method (N=16) at stage 1.

Question	Category	n	%
1. Gender	female	5	31.3
	male	11	68.8
2. Age	30-39	2	12.5
	40-49	5	31.3
	50-59	7	43.8
	>60	2	12.5
3. Family status	unmarried	4	25.0
	married	12	75.0
4. Do you have children?	no	4	25.0
	yes	12	75.0
5. In what area have you been living permanently for the last few years?	village	1	6.3
	town	2	12.5
	city	13	81.3
6. What is your medical speciality?	rheumatology	1	6.3
	cardiology	2	12.5
	internal medicine	2	12.5
	endocrinology	2	12.5
	psychiatry	1	6.3
	oncology	1	6.3
	general medicine	2	12.5
	orthopedics	1	6.3
	neurology	1	6.3
	vascular surgery	1	6.3
	nephrology	1	6.3
	pulmonology	1	6.3
9. At which university did you do your undergraduate studies?	International	1	6.3
	Greek	15	93.8
9a. If you studied at an international or other university, which one; (n1=1)	Iasou	1	6.3
10. Do you have any other studies or specializations (other than your major speciality)?	no	4	25.0
	yes	12	75.0
10a. If yes, what are they? (please fill in anything relevant) (n1=12)	MSc	1	8.3
	PhD	8	66.7
	Post-Doc	1	8.3
	Other	2	16.7
11. What is your employment status?	private	9	56.3
	public	3	18.8
	university	4	25.0
12. What region do you work in?	town	2	12.5
	city	14	87.5
13. Compared to other people your age, would you say that your health is:	very good	13	81.3
	excellent	3	18.8

14. Compared to other people your age, would you say that your physical condition/ability is:	moderate<br>very good	5<br>11	31.3<br>68.8
15. Do you smoke?	no<br>yes<br>quit>1 year	9<br>4<br>3	56.3<br>25.0<br>18.8
15a. If yes, how many cigarettes per day do you smoke? (n1=4)	<5<br>5-10<br>15-20	2<br>1<br>1	50.0<br>25.0<br>25.0
16. Have you ever been taught physical activity and exercise subject(s) at the medical school you attended?	no<br>yes	14<br>2	87.5<br>12.5
16a. If yes, which physical activity and exercise subject(s) have you been taught? Please specify (n1=9)	aerobic exercise<br>muscular strengthening in team sports<br>exercise on the cardiovascular system<br>effect of exercise on cardiovascular and musculoskeletal system<br>exercise and health<br>swimming<br>rehabilitation<br>physical activity in chronic diseases<br>effect of aerobic exercise on metabolism	1<br>1<br>1<br>1<br>1<br>1<br>1<br>1<br>1	7,1<br>7,1<br>7,1<br>7,1<br>7,1<br>7,1<br>7,1<br>7,1<br>7,1
17. Are you informed/educated about physical activity and exercise issues?	no<br>yes	5<br>11	31.3<br>68.8
17a.If yes, please indicate the sources from which you are informed/educated about physical activity and exercise (n1=12)	media<br>internet<br>journals	2<br>7<br>3	12.5<br>43.8<br>18.8
17b. If you have already participated in a seminar or lifelong learning program in physical activity and exercise, how many hours of training have participated in? (n1=6)	don't remember<br>1-3	4<br>2	25.0<br>12.5
18. Do you wish to participate in a future seminar or training program on physical activity and exercise?	no<br>yes	4<br>12	25.0<br>75.0
18a. If yes, what kind of training would you like to participate in? (n1=12)	synchronous distance<br>hybrid<br>live	4<br>7<br>1	25.0<br>43.8<br>6.3

18b. If yes, how many hours would you like to be trained in a physical activity and exercise training seminar or program? (n1=12)	1-3<br>4-10	6<br>6	37.5<br>37.5
19. Do you know about physical activity and exercise?	no<br>yes	2<br>14	12.5<br>87.5
19a.If yes, what is your knowledge level? (n1=14)	2<br>3<br>4	3<br>4<br>7	18.8<br>25.0<br>43.8
20. Do you know about counselling patients with chronic non-communicable diseases on physical activity and exercise?	no<br>yes	3<br>13	18.8<br>81.3
20a. If yes, what is your knowledge level? (n1=14)	1<br>2<br>3<br>4<br>5	2<br>3<br>5<br>3<br>1	12.5<br>18.8<br>31.3<br>18.8<br>6.3
21. Do you know about prescribing physical activity and exercise for patients with chronic non-communicable diseases?	no<br>yes	12<br>4	75.0<br>25.0
21a.If yes, what is your knowledge level? (n1=5)	1<br>3<br>5	1<br>3<br>1	6.3<br>18.8<br>6.3
22. Do you know about assessing physical activity and exercise levels in patients with chronic non-communicable diseases?	no<br>yes	10<br>6	62.5<br>37.5
22a. If yes, what is your knowledge level? (n1=6)	2<br>3<br>4<br>5	2<br>1<br>2<br>1	12.5<br>6.3<br>12.5<br>6.3
23. Do you know the World Health Organization (WHO) guidelines on physical activity for health?	no<br>yes	11<br>5	68.8<br>31.3
**Question**	**Median**	**IQR**	**Min-Max**
7. What is the year you obtained your speciality?	2006	7.5/30/9	1990-2020
8. How many years have you been working as a physician?	22.5	7.39/26/10	11-37

Interquartile range (IQR); minimum-maximum values(min-max); absolute frequencies (n); relative frequencies (%); number of experts who answered the optional questions (n1).

Table S2. Experts' agreement levels at the end of the e-Delphi round 1 (N=16) at stage 1 (Questionnaire S1).

Question/ Item	Low Importance			Neutral	High Importance				Mdn (IQR)
	n_1(%)	n_2(%)	n_3(%)	n_4(%)	n_5(%)	n_6(%)	n_7(%)	n_{5+6+7} (%)	
**Knowledge of physical activity and exercise**									
24	0	0	1(6.3)	1(6.3)	3(18.8)	6(37.5)	5(31.3)	14 (87,5%)	6(2)
25	0	0	1(6.3)	1(6.3)	3(18.8)	6(37.5)	5(31.3)	14 (87,5%)	6(2)
26*	0	1(6.3)	1(6.3)	1(6.3)	3(18.8)	4(25)	6(37.5)	13 (81,25%)	6(2)
27	0	0	1(6.3)	1(6.3)	1(6.3)	6(37.5)	7(43.8)	14 (87,5%)	6(1)
28	0	0	2(12.5)	1(6.3)	2(12.5)	4(25)	7(43.8)	13 (81,25%)	6(2)
29	0	0	2(12.5)	1(6.3)	2(12.5)	6(37.5)	5(31.3)	13(81,25%)	6(2)
30	0	0	1(6.3)	0	1(6.3)	6(37.5)	8(50)	15 (93,8%)	6.5(1)
31	0	0	0	0	3(18.8)	6(37.5)	7(43.8)	16 (100%)	6(1)
32	0	0	0	1(6.3)	3(18.8)	4(25)	8(50)	15 (93,8%)	6.5(2)
33	0	0	0	1(6.3)	4(25)	3(18.8)	8(50)	15 (93,8%)	6.5(2)
34	1(6.3)	0	0	1(6.3)	4(25)	3(18.8)	7(43.8)	14 (87,5%)	6(2)
35	1(6.3)	0	1(6.3)	2(12.5)	3(18.8)	5(31.3)	4(25)	12 (75%)	6(3)
36	1(6.3)	0	1(6.3)	2(12.5)	5(31.3)	5(31.3)	2(12.5)	12 (75%)	5(2)
37	2(12.5)	1(6.3)	0	1(6.3)	0	8(50)	4(25)	12 (75%)	6(2)
38	2(12.5)	1(6.3)	0	1(6.3)	0	8(50)	4(25)	12 (75%)	6(2)
39	2(12.5)	0	1(6.3)	0	1(6.3)	8(50)	4(25)	13 (81,25%)	6(2)
40	0	0	0	2(12.5)	1(6.3)	9(56.3)	4(25)	14 (87,5%)	6(1)
41	1(6.3)	0	0	3(18.8)	3(18.8)	6(37.5)	3(18.8)	12 (75%)	6(2)
42	0	0	1(6.3)	2(12.5)	3(18.8)	5(31.3)	5(31.3)	13 (81,25%)	6(2)
43	0	2(12.5)	0	2(12.5)	2(12.5)	7(43.8)	3(18.8)	12 (75%)	6(2)
44	0	0	0	0	1(6.3)	9(56.3)	6(37.5)	16 (100%)	6(1)
45	0	0	0	0	3(18.8)	7(43.8)	6(37.5)	16 (100%)	6(1)
46	0	0	0	1(6.3)	2(12.5)	7(43.8)	6(37.5)	15 (93,8%)	6(1)
47	0	0	2(12.5)	0	0	10(62.5)	4(25)	14 (87,5%)	6(1)
48	0	0	1(6.3)	2(12.5)	1(6.3)	6(37.5)	6(37.5)	13 (81,25%)	6(2)

49	0	0	0	0	2(12.5)	6(37.5)	8(50)	16 (100%)	6.5(1)
50	0	0	1(6.3)	1(6.3)	2(12.5)	6(37.5)	6(37.5)	14 (87,5%)	6(2)
51	0	1(6.3)	0	0	3(18.8)	5(31.3)	7(43.8)	15 (93,8%)	6(2)
52***	1(6.3)	0	1(6.3)	4(25)	1(6.3)	4(25)	5(31.3)	10 (62,5%)	6(3)
**Attitudes on physical activity and exercise**									
53	1(6.3)	0	1(6.3)	2(12.5)	1(6.3)	4(25)	7(43.8)	12 (75%)	6(3)
54	0	1(6.3)	0	1(6.3)	2(12.5)	5(31.3)	7(43.8)	14 (87,5%)	6(2)
55	0	0	0	0	2(12.5)	5(31.3)	9(56.3)	16 (100%)	7(1)
56	0	0	0	1(6.3)	1(6.3)	5(31.3)	9(56.3)	15 (93,8%)	7(1)
57*	0	0	0	0	1(6.3)	6(37.5)	9(56.3)	16 (100%)	7(1)
58	0	0	0	1(6.3)	1(6.3)	5(31.3)	9(56.3)	15 (93,8%)	7(1)
59*	0	0	0	1(6.3)	2(12.5)	5(31.3)	8(50)	15 (93,8%)	6.5(1)
60	0	0	0	3(18.8)	1(6.3)	6(37.5)	6(37.5)	13 (81,25%)	6(2)
61	0	0	0	0	0	7(43.8)	9(56.3)	16 (100%)	7(1)
62	0	0	1(6.3)	0	1(6.3)	5(31.3)	9(56.3)	15 (93,8%)	7(1)
63	0	0	0	2(12.5)	2(12.5)	6(37.5)	6(37.5)	14 (87,5%)	6(2)
64*	1(6.3)	1(6.3)	0	1(6.3)	6(37.5)	4(25)	3(18.8)	13 (81,25%)	5(1)
65*	0	0	2(12.5)	1(6.3)	1(6.3)	7(43.8)	5(31.3)	13 (81,25%)	6(2)
66*	0	0	0	0	4(25)	7(43.8)	5(31.3)	16 (100%)	6(2)
67***	0	1(6.3)	0	6(37.5)	4(25)	3(18.8)	2(12.5)	9 (56,25%)	5(2)
68***	0	0	1(6.3)	5(31.3)	2(12.5)	3(18.8)	5(31.3)	10 (62,5%)	6(2)
69	0	0	1(6.3)	2(12.5)	4(25)	4(25)	5(31.3)	13 (81,25%)	6(2)
70***	1(6.3)	1(6.3)	1(6.3)	4(25)	4(25)	2(12.5)	3(18.8)	9 (56,25%)	5(2)
**Intentions on physical activity and exercise**									
71*	0	0	0	1(6.3)	3(18.8)	4(25)	8(25)	15 (93,8%)	6.5(2)
72*	0	0	0	2(12.5)	3(18.8)	6(37.5)	5(31.3)	14 (87,5%)	6(2)
73*	0	0	0	1(6.3)	1(6.3)	7(43.8)	7(43.8)	15 (93,8%)	6(1)
74*	0	0	0	1(6.3)	3(18.8)	7(43.8)	5(31.3)	15 (93,8%)	6(2)
75	0	0	0	2(12.5)	1(6.3)	8(50)	5(31.3)	14 (87,5%)	6(1)
76*	0	0	2(12.5)	2(12.5)	3(18.8)	6(37.5)	3(18.8)	12 (75%)	6(2)
77*	0	0	0	1(6.3)	1(6.3)	5(31.3)	9(56.3)	15 (93,8%)	7(1)

78**	1(6.3)	4(25)	1(6.3)	1(6.3)	1(6.3)	3(18.8)	5(31.3)	9 (56,25%)	5.5(5)
79**	1(6.3)	3(18.8)	2(12.5)	1(6.3)	1(6.3)	4(25)	4(25)	9 (56,25%)	5.5(5)
80**	1(6.3)	1(6.3)	3(18.8)	4(25)	1(6.3)	2(12.5)	4(25)	7 (43,75%)	4(4)
81**	1(6.3)	3(18.8)	3(18.8)	3(18.8)	0	4(25)	2(12.5)	6 (37,5%)	4(4)
82**	1(6.3)	3(18.8)	3(18.8)	2(12.5)	0	4(25)	3(18.8)	7 (43,75%)	4(4)
83**	1(6.3)	3(18.8)	2(12.5)	2(12.5)	0	5(31.3)	3(18.8)	8 (50%)	5(4)
84**	1(6.3)	3(18.8)	1(6.3)	2(12.5)	2(12.5)	4(25)	3(18.8)	9 (56,25%)	5(4)
84a**	1(6.3)	1(6.3)	0	2(12.5)	2(12.5)	5(31.3)	5(31.3)	12 (75%)	6(3)
85*	0	0	1(6.3)	2(12.5)	2(12.5)	7(43.8)	4(25)	13 (81,25%)	6(2)
**Abilities on physical activity and exercise**									
86	0	0	0	2(12.5)	3(18.8)	7(43.8)	4(25)	14 (87,5%)	6(2)
87	0	0	0	3(18.8)	3(18.8)	7(43.8)	3(18.8)	13 (81,25%)	6(1)
88	0	0	0	4(25)	2(12.5)	7(43.8)	3(18.8)	12 (75%)	6(2)
89	0	0	0	1(6.3)	3(18.8)	9(56.3)	3(18.8)	15 (93,8%)	6(1)
90*	0	1(6.3)	0	2(12.5)	3(18.8)	6(37.5)	4(25)	13 (81,25%)	6(2)
91	0	0	0	1(6.3)	2(12.5)	6(37.5)	7(43.8)	15 (93,8%)	6(1)
**Behaviour on physical activity and exercise**									
92	0	0	0	0	1(6.3)	8(50)	7(43.8)	16 (100%)	6(1)
93	0	1(6.3)	0	1(6.3)	1(6.3)	8(50)	5(31.3)	14 (87,5%)	6(1)
94	0	0	0	0	0	8(50)	8(50)	16 (100%)	6.5(1)
94a*	0	0	0	4(25)	1(6.3)	6(37.5)	5(31.3)	12 (75%)	6(3)
94b*	0	0	0	0	1(6.3)	6(37.5)	9(56.3)	16(100%)	7(1)
94c*	0	0	0	1(6.3)	2(12.5)	8(50)	5(31.3)	15 (93,8%)	6(1)
94d*	0	0	1(6.3)	1(6.3)	2(12.5)	6(37.5)	6(37.5)	14 (87,5%)	6(2)
95	0	1(6.3)	0	3(18.8)	1(6.3)	8(50)	3(18.8)	12 (75%)	6(2)
95a	1(6.3)	0	0	2(12.5)	1(6.3)	8(50)	4(25)	13 (81,25%)	6(2)
96*	0	1(6.3)	0	3(18.8)	0	8(50)	4(25)	12 (75%)	6(2)
96a	0	1(6.3)	0	2(12.5)	0	7(43.8)	6(37.5)	13 (81,25%)	6(1)
97	0	0	2(12.5)	0	2(12.5)	9(56.3)	3(18.8)	14 (87,5%)	6(1)
97a	0	0	0	0	3(18.8)	7(43.8)	6(37.5)	16 (100%)	6(1)
98*	1(6.3)	0	2(12.5)	1(6.3)	4(25)	4(25)	4(25)	12 (75%)	5.5(3)

98a	0	0	0	3(18.8)	1(6.3)	6(37.5)	6(37.5)	13 (81,25%)	6(2)
99*	0	0	2(12.5)	1(6.3)	4(25)	6(37.5)	3(18.8)	13 (81,25%)	6(1)
100*	0	0	0	0	3(18.8)	8(50)	5(31.3)	16 (100%)	6(1)
101***	3(18.8)	1(6.3)	0	4(25)	2(12.5)	4(25)	2(12.5)	8 (50%)	4.5(4)
102	1(6.3)	1(6.3)	0	3(18.8)	1(6.3)	5(31.3)	5(31.3)	11 (68,75%)	6(3)
103	1(6.3)	0	0	1(6.3)	1(6.3)	9(56.3)	4(25)	14 (87,5%)	6(1)
104	2(12.5)	0	2(12.5)	3(18.8)	2(12.5)	4(25)	3(18.8)	9 (56,25%)	5(3)
105	0	0	0	0	0	6(37.5)	10(62.5)	16 (100%)	7(1)

Minimum-maximum values (min-max); absolute frequencies (n); relative frequencies (%); median value (Mdn); absolute and relative frequencies of the answers 1,2,3,4,5,6,7 respectively [n1 (%), n2(%), n3(%), n4(%), n5(%), n6(%), n7(%)].

* Questions reworded/clarified following expert comments without changing their content.

** Questions removed due to negative consensus.

***Questions that were re-evaluated based on the consensus rule (consensus ≥25% for neutral answer 4) and the research team decision.

The questions in the above Table S2 are presented numerically and appear in full in Questionnaire S1.

Table S3. Experts' agreement levels at the end of the e-Delphi round 2 (n=16) and the content validity ratio (CVR) (N=16) at stage 1(Questionnaire S2).

Question/Item	n_{1+2+3} (%)	n_{4}(%)	n_{5+6+7} (%)	Mdn (IQR)	CVR
**Knowledge of physical activity and exercise**					
24	1(6,25)	0	15 (93,8)	7(1)	0.87
25	1(6,25)	0	15 (93,8)	7(1)	0.87
M26	0	0	16 (100)	7(1)	1
27	1(6,25)	0	15 (93,8)	7(1)	0.87
28	1(6,25)	1(6,25)	14 (87,5)	6(1)	0.75
29	1(6,25)	1(6,25)	14 (87,5)	6(2)	0.75
30	0	0	16 (100)	7(1)	1
31	0	0	16 (100)	7(1)	1
32	0	0	16 (100)	6.5(2)	1
33	0	1(6,25)	15 (93,8)	6(2)	0.87
34	2(12,5)	0	14 (87,5)	6(2)	0.75
35	1(6,25)	1(6,25)	13 (81,25)	5.5(2)	0.63
36	1(6,25)	5 (31,25)	10 (62,5)	6(3)	0.25
37	0	1(6,25)	15 (93,8)	6(2)	0.87
38	0	1(6,25)	15 (93,8)	6(2)	0.87
39	0	2(12,5)	14 (87,5)	6(2)	0.75
40	0	3	13 (81,25)	6(2)	0.63
41	0	3	13 (81,25)	6(2)	0.63
42	0	1(6,25)	15 (93,8)	6.5(2)	0.87
43	1(6,25)	0	15 (93,8)	6(2)	0.87
44	0	0	16 (100)	7(1)	1
45	0	0	16 (100)	6.5(1)	1
46	0	0	16 (100)	7(1)	1
47	0	1(6,25)	15 (93,8)	6(2)	0.87
48	0	0	16 (100)	6.5(2)	1
49	0	0	16 (100)	6.5(1)	1
50	0	1(6,25)	15 (93,8)	6.5(2)	0.87
51	0	0	16 (100)	6.5(2)	1
52	2(12,5)	2(12,5)	12 (75)	6(3)	0.5
**Attitudes on physical activity and exercise**					
53	2(12,5)	2(12,5)	12 (75)	6.5(3)	0.5
54	2(12,5)	1(6,25)	13 (81,25)	6.5(2)	0.63
55	0	2(12,5)	14 (87,5)	7(2)	0.75
56	1(6,25)	1(6,25)	14 (87,5)	7(2)	0.75
M57	0	1(6,25)	15 (93,8)	6(2)	0.87
58	0	0	16 (100)	6.5(2)	1
M59	1(6,25)	0	15 (93,8)	6(2)	0.87
60	2(12,5)	1(6,25)	13 (81,25)	6(2)	0.63
61	2(12,5)	0	14 (87,5)	6.5(2)	0.75
62	1(6,25)	1(6,25)	14 (87,5)	6.5(2)	0.75
63	0	1(6,25)	15 (93,8)	6(2)	0.87
M64	3	0	13 (81,25)	5.5(2)	0.63
M65	0	1(6,25)	15 (93,8)	6(2)	0.87
M66	0	1(6,25)	15 (93,8)	6(2)	0.87

M67	0	1(6,25)	15 (93,8)	6(2)	0.87
**Intentions on physical activity and exercise**					
M71	0	1(6,25)	15 (93,8)	6(2)	0.87
M72	0	1(6,25)	13 (81,25)	6(2)	0.63
M73	0	0	16 (100)	6(2)	1
M74	0	0	16 (100)	6.5(2)	1
75	0	1(6,25)	15 (93,8)	6.5(2)	0.87
M76	1(6,25)	0	15 (93,8)	5.5(2)	0.87
M77	0	0	16 (100)	6(1)	1
M85	0	1(6,25)	15 (93,8)	6(2)	0.87
**Abilities on physical activity and exercise**					
86	0	0	16 (100)	6.5(2)	1
87	1(6,25)	1(6,25)	15 (93,8)	6(2)	0.87
88	0	1(6,25)	15 (93,8)	6(2)	0.87
89	0	0	16 (100)	6.5(2)	1
M90	1(6,25)	0	15 (93,8)	6(2)	0.87
91	0	0	16 (100)	7(2)	1
**Behaviour on physical activity and exercise**					
92	0	0	16 (100)	6(1)	1
93	0	2(12,5)	14 (87,5)	6(2)	0.75
94	0	0	16 (100)	7(2)	1
M94a	1(6,25)	1(6,25)	14 (87,5)	5.5(2)	0.75
M94b	1(6,25)	4(25)	11 (68,75)	5.5(3)	0.36
M94c	0	2(12,5)	14 (87,5)	6(2)	0.75
M94d	0	1(6,25)	15 (93,8)	6(2)	0.87
M94e	0	1(6,25)	15 (93,8)	6(2)	0.87
M94f	0	1(6,25)	15 (93,8)	6(2)	0.87
95	1(6,25)	3	12 (75)	5.5(3)	0.5
95a	0	2(12,5)	14 (87,5)	6(2)	0.75
M96	2(12,5)	3	11 (68,75)	5(3)	0.36
96a	0	2(12,5)	14 (87,5)	6(2)	0,75
97	2(12,5)	4(25)	10 (62,5)	5(3)	0.25
97a	0	2(12,5)	14 (87,5)	6(2)	0,75
M98	1(6,25)	4(25)	11 (68,75)	5.5(3)	0.36
98a	0	2(12,5)	14 (87,5)	6(2)	0,75
M99	0	3	13 (81,25)	6(2)	0.63
M100	0	2(12,5)	14 (87,5)	6(2)	0,75
M100a	0	1(6,25)	15 (93,8)	6(2)	0,87
M100b	1(6,25)	5(31,25)	10 (62,5)	5(3)	0.25
M100c	0	3	13 (81,25)	6(2)	0.63
M100d	1(6,25)	1(6,25)	14 (87,5)	6(2)	0,75
M100e	2(12,5)	3	11 (68,75)	6(3)	0.36
105	0	0	16 (100)	7(1)	1

Absolute and relative frequencies of the sum of the answers 1,2,3 (negative consensus) respectively [n1+2+3 (%)]; absolute and relative frequencies of the answer 4 (neutral) [n4(%)]; absolute and relative frequencies of the sum of the answers 5,6,7 (positive consensus) respectively [n5+6+7(%)]; modified questions (M).

Notes:
1. Eight questions (78, 79, 80, 81, 82, 83, 84, and 84a), which were present in the round 1, were removed from the questionnaire of the round 2.
2. 23 questions were reworded and/or separated, giving 30 questions to the e-Delphi round 2 questionnaire and having the letter M (MODIFIED) in front of them.
3. The questions in the above Table S3 are presented numerically and appear in full in Questionnaire S2.

Table S4. Wilcoxon p-value stability test between e-Delphi rounds 1 and 2 (N=16) at stage 1 (Questionnaires S1 and S2).

Compared questions of e-Delphi rounds 1 -2	e-Delphi rounds	Median (Interquartile Range, IQR)	Wilcoxon p-value
**Knowledge of physical activity and exercise**			
24-24	1st	6(2)	0.154
	2nd	7 (1)	
25-25	1st	6(2)	0.112
	2nd	7 (1)	
26-M26	1st	6(2)	0.036
	2nd	7 (1)	
27-27	1st	6(1)	0.583
	2nd	7 (1)	
28-28	1st	6(2)	0.469
	2nd	6 (1)	
29-29	1st	6(2)	0.473
	2nd	6 (2)	
30-30	1st	6,5(1)	0.271
	2nd	7 (1)	
31-31	1st	6(1)	0.589
	2nd	7 (1)	
32-32	1st	6.5(2)	0.871
	2nd	6.5(2)	
33-33	1st	6.5(2)	1
	2nd	6(2)	
34-34	1st	6(2)	0.784
	2nd	6(2)	
35-35	1st	6(3)	0.612
	2nd	5.5(2)	
36-36	1st	5(2)	0.646
	2nd	6 (3)	
37-37	1st	6(2)	0.369

	2nd	6(2)	
38-38	1st	6(2)	0.260
	2nd	6(2)	
39-39	1st	6(2)	0.566
	2nd	6(2)	
40-40	1st	6(1)	0.891
	2nd	6(2)	
41-41	1st	6(2)	0.394
	2nd	6(2)	
42-42	1st	6(2)	0.337
	2nd	6.5(2)	
43-43	1st	6(2)	0.180
	2nd	6(2)	
44-44	1st	6(1)	0.763
	2nd	7(1)	
45-45	1st	6(1)	0.564
	2nd	6.5(1)	
46-46	1st	6(1)	0.083
	2nd	7(1)	
47-47	1st	6(1)	0.711
	2nd	6(2)	
48-48	1st	6(2)	0.272
	2nd	6.5(2)	
49-49	1st	6.5(1)	0.763
	2nd	6.5(1)	
50-50	1st	6(2)	0.452
	2nd	6.5(2)	
51-51	1st	6(2)	0.582
	2nd	6.5(2)	
52-52	1st	6(3)	0.686
	2nd	6(3)	
**Attitudes on physical activity and exercise**			
53-53	1st	6(3)	0.857
	2nd	6.5(3)	
54-54	1st	6(2)	0.787
	2nd	6.5(2)	
55-55	1st	7(1)	0.476
	2nd	7(2)	
56-56	1st	7(1)	0.571
	2nd	7(2)	
57-M57	1st	7(1)	0.142
	2nd	6(2)	
58-58	1st	7(1)	0.672
	2nd	6.5(2)	
59-M59	1st	6,5(1)	0.587
	2nd	6(2)	

60-60	1st	6(2)	0.753
	2nd	6(2)	
61-61	1st	7(1)	0.142
	2nd	6.5(2)	
62-62	1st	7(1)	0.468
	2nd	6.5(2)	
63-63	1st	6(2)	0.836
	2nd	6(2)	
64-M64	1st	5(1)	0.959
	2nd	5.5(2)	
**Intentions on physical activity and exercise**			
71-M71	1st	6.5(2)	0.256
	2nd	6(2)	
72-M72	1st	6(2)	0.436
	2nd	6(2)	
73-M73	1st	6(1)	0.507
	2nd	6(2)	
74-M74	1st	6(2)	0.490
	2nd	6.5(2)	
75-75	1st	6(1)	0.927
	2nd	6.5(2)	
76-M76	1st	6(2)	0.59
	2nd	5.5(2)	
77-M77	1st	7(1)	0.493
	2nd	6(2)	
85-M85	1st	6(20	0.437
	2nd	6(2)	
**Abilities on physical activity and exercise**			
86-86	1st	6(2)	0.244
	2nd	6.5(2)	
87-87	1st	6(1)	0.525
	2nd	6(2)	
88-88	1st	6(2)	0.185
	2nd	6(2)	
89-89	1st	6(1)	0.218
	2nd	6.5(2)	
90-M90	1st	6(20	0.521
	2nd	6(2)	
91-91	1st	6(1)	0.951
	2nd	7(2)	
**Behaviour on physical activity and exercise**			
92-92	1st	6(1)	0.405
	2nd	6(2)	
93-93	1st	6(1)	0.874
	2nd	6(2)	

94-94	1st	6.5(1)	0.527
	2nd	7(2)	
94b-M94d	1st	7(1)	0.191
	2nd	6(2)	
94c-M94e	1st	6(1)	0.658
	2nd	6(2)	
94d-M94f	1st	6(2)	0.829
	2nd	6(2)	
95-95	1st	6(2)	0.804
	2nd	5.5(3)	
95a-95a	1st	6(2)	0.877
	2nd	6(2)	
96-M96	1st	6(2)	0.322
	2nd	5(3)	
96a-96a	1st	6(1)	0.964
	2nd	6(2)	
97-97	1st	6(1)	0.304
	2nd	5(3)	
97a-97a	1st	6(1)	0.293
	2nd	6(2)	
98-M98	1st	5.5(3)	0.873
	2nd	5.5(3)	
98a-98a	1st	6(2)	0.832
	2nd	6(2)	
99-M99	1st	6(1)	0.404
	2nd	6(2)	
100-M100a	1st	6(3)	0.377
	2nd	6(2)	
101-M100b	1st	4.5(4)	0.230
	2nd	5(3)	
102-M100c	1st	6(3)	0.501
	2nd	6(2)	
103-M100d	1st	6(1)	0.582
	2nd	6(2)	
104-M100e	1st	5(3)	0.190
	2nd	6(3)	
105-105	1st	7(1)	0.317
	2nd	7(1)	

IQR, interquartile range

Notes:

1. The modified questions (Questionnaire S2) were compared with their counterparts in the e-Delphi round 1 questionnaire (Questionnaire S1), which had the same numbering but were not preceded by the letter M. The modified questions compared with their round 1 counterparts in the above Table S4 showed no change in content or meaning.
2. The e-Delphi round 2 questions were not compared with their round 1 counterparts due to mismatches (they were collapsed or split): M65, M66, M67, M68, M69, M70, M94a, M94b, M94c.
3. The following questions due to a change in their numbering, not in their content, (they were collapsed or split), were compared as follows:
- Question 94b of round 1 was compared with M94d of round 2.
- Question 94c of e-Delphi round 1 was compared with M94e of the round 2.
- Question 94d of e-Delphi round 1 was compared with M94f of the round 2.
- Questions 100, 101, 102, 102, 103 and 104 of e-Delphi round 1 were compared with M100a, M100b, M100c, M100d and M100e of the round 2 respectively.

4.. The questions in the above Table S4 are presented numerically and appear in full in Questionnaires S1 and S2.

Questionnaire S3. E-Delphi round 2 (modified) questionnaire at stage 2 (English translation of the Greek questionnaire).

I. Demographic, professional, and educational characteristics

1. Gender	
Female	☐
Male	☐

2. Age	
20-29	☐
30-39	☐
40-49	☐
50-59	☐
Over 60	☐

3. Family status	
Unmarried	☐
Married	☐
Divorced	☐
Widowed	☐
Cohabitation	☐

4. Do you have children?	
Yes	☐
No	☐

5. In what area have you been living permanently for the last few years?	
City	☐
Town	☐
Village	☐

6. What is your medical speciality?	

6a. What is the year you obtained your speciality?	

7. How many years have you been working as a physician?	

8. At which university did you do your undergraduate studies?	
Greek	☐
International	☐
Other	☐

8a. If you studied at an international or other university, which one;	

9. Do you have any other studies or specializations (other than your major specialty)?	
Yes	☐
No	☐

9a. If yes, what are they?	
Bachelor's degree	☐
Master's degree	☐
Doctorate (PhD)	☐
Other	☐

9b. If you answered “Other”, what are they? Please specify	

10. What is your employment status?	
Private medical office	☐
Private Clinic	☐
Public sector (health centre - hospital - fund)	☐
University	☐
Other (administrative position, position of responsibility, etc.)	☐

10a. If you answered “Other”, which one is it? Please specify	

11. What region do you work in?	
City	☐
Town	☐
Village	☐

11a. Which Medical Association do you belong to?	
Trikala	☐
Karditsa	☐
Other	☐

12. How many patients do you see in one day on average?	
<5	☐
5-10	☐
11-20	☐
>20	☐

Don’t remember	☐

13. Compared to other people your age, would you say that your health condition is:	
Excellent	☐
Very good	☐
Moderate	☐
Poor	☐
Very poor	☐

14. Compared to other people your age, would you say that your physical condition/ability is:	
Excellent	☐
Very good	☐
Moderate	☐
Poor	☐
Very poor	☐

15. Do you smoke?	
Yes	☐
No, never	☐
No, I quit recently	☐
No, I quit at least 1 year ago	☐

15a. If yes, how many cigarettes per day do you smoke?	
<10	☐
10-20	☐
11-20	☐
21-30	☐
<31	☐
I don't smoke every day but occasionally during the week or month	☐
Don’t remember	☐

15b. If you only smoked in the past, how long in total did you smoke for (in years)?	

16. Have you ever been taught physical activity and exercise subject(s) at the medical school you attended?	
Yes	☐
No	☐
Don't remember	☐

16a. If yes, which physical activity and exercise subject(s) have you been taught? Please state	

16b. If not, which physical activity and exercise module(s) would you prefer to have been taught? Please briefly state	

17. Are you informed/educated about physical activity and exercise issues in the context of lifelong learning?	
Yes	☐
No	☐

17a.If yes, please indicate the sources from which you are informed/educated about physical activity and exercise (one or more responses)	
Scientific journals and books	☐
Scientific bodies/exercise bodies	☐
Authoritative medical internet resources (internet)	☐
Media	☐
Seminars/conferences	☐
Lifelong learning programs	☐
Other	☐

17b. If you have already participated in a seminar or lifelong learning program in physical activity and exercise, how many hours of training have participated in?	
<1	☐
1-3	☐
4-10	☐
11-40	☐

>40	☐
Don’t remember	☐

17c. If you have attended a seminar or lifelong learning programmes on physical activity and exercise, in which subject(s) have you been trained? Please state	

18. Do you wish to participate in a seminar or training program on physical activity and exercise in the future?	
Yes	☐
No	☐

18a. If yes, what kind of training would you like to participate in?	
Synchronous distance	☐
In-person	☐
Hybrid (distance and in-person)	☐
Other	☐
18a1. If you answered another, which one is it? Please specify…….	

18b. If yes, how many hours would you like to be trained in a physical activity and exercise training seminar or program?	
<1	☐
1-3	☐
4-10	☐
11-40	☐
>40	☐

18c. If yes, in which subject(s) would you like to be trained? Please state	

19. Do you have knowledge about the benefits of physical activity and exercise on chronic non-communicable diseases?	
Yes	☐
No	☐

19a.If yes, what is your knowledge level?	Very low	Low	Medium	Good	Very good
	☐	☐	☐	☐	☐

20.Do you have knowledge of counselling patients with chronic non-communicable diseases on physical activity and exercise?	
Yes	☐
No	☐

20a. If yes, what is your knowledge level?	Very low	Low	Medium	Good	Very good
	☐	☐	☐	☐	☐

21. Do you have knowledge of prescribing physical activity and exercise for patients with chronic non-communicable diseases?	
Yes	☐
No	☐

21a.If yes, what is your knowledge level?	Very low	Low	Medium	Good	Very good
	☐	☐	☐	☐	☐

22. Do you have knowledge in assessing physical activity and exercise levels in patients with chronic non-communicable diseases?	
Yes	☐
No	☐

22a. If yes, what is your knowledge level?	Very low	Low	Medium	Good	Very good
	☐	☐	☐	☐	☐

23 . Do you know the World Health Organization (WHO) guidelines on physical activity for health?	
Yes	☐
No	☐

II. Knowledge of physical activity and exercise

24.Physical activity is:<br><br>**Choose one of the following responses:**<br>-The involvement in sports<br>-Walking<br>-Exercise<br>-Any physical movement<br>- Don't know
25. Exercise is:<br><br>**Choose one of the following responses:**<br>- Engaging in sports<br>- Walking<br>- It is synonymous with physical activity<br>- Any physical movement<br>- A structured and planned program of physical activity aimed at improving physical fitness<br>- Don't know
26. When we say, "major principles of exercise program design", we mean:<br><br>**Choose one of the following responses:**<br>-The intensity, duration, frequency, and instrument of the exercise<br>-The physical fitness of the participant<br>-The specificity, enjoyment, and progressiveness of the exercise<br>-All the above<br>-Don't know
27.The intensity of the exercise is:<br><br>**Choose one of the following responses:**<br>- How many minutes we exercise<br>- How many times a week we exercise<br>- The difficulty of the exercise<br>- The exercise equipment<br>- Don't know
28. The duration of the exercise is:<br><br>**Choose one of the following responses:**<br>- How many minutes do we exercise<br>- How many times a week we exercise<br>- The difficulty of the exercise<br>- The exercise equipment<br>- Don't know

29. The frequency of exercise is:<br><br>**Choose one of the following responses:**<br>- How many minutes do we exercise<br>- How many times a week we exercise<br>- The difficulty of the exercise<br>- The exercise equipment<br>- How many times a month we exercise<br>- Don't know
30. Which of the following is an indicator of exercise intensity?<br><br>**Choose one of the following responses:**<br>-The heart rate<br>-The training duration<br>-The workouts per week<br>-The steps of the trainee<br>-All the above<br>- Don't know
31.Which of the following is a resistance training exercise?<br><br>**Choose one of the following responses:**<br>- Running on the treadmill<br>- Aerobics program<br>- Abdominal exercises<br>- Stretches<br>- None of the above<br>- Don't know
32. Which of the following is aerobic exercise?<br><br>**Choose one of the following responses:**<br>-Weight training in the gym<br>-Single bar lifts<br>-100 meters sprint<br>-Swimming<br>-None of the above<br>- Don't know
33.Which of the following is a mobility exercise?<br><br>**Choose one of the following responses:**<br>- Tensions<br>- Balance exercises<br>- Pilates<br>- Yoga<br>-All the above<br>- Don't know

34. Which of the following is a high-intensity interval exercise?

Choose one of the following responses:
- Pilates
- Yoga
- 10 sprints with a break in between
- 5 sets of one weight training exercise with a break in between
- 2 runs per day (morning-evening) with an 8-hour break in between
- Don't know

35. "PAR-Q and YOU" are:

Choose one of the following responses:
- Type of treadmill
- Type of bicycle
- Type of aerobic training
- Type of resistance training
- Training suitability questionnaire
- Questionnaire on predisposing factors for cardiovascular disease
- Don't know

36. The "MET" is:

Choose one of the following responses:
- Type of dumbbell
- Type of stretches
- Unit of measurement of exercise intensity
- Type of resistance
- Aerobic program
- None of the above
- Don't know

37. The risk of a non-fatal heart attack during vigorous exercise in asymptomatic individuals is:

Choose one of the following responses:
- 1 episode per approximately 10 million hours of training
- 1 event per approximately 5 million hours of training
- 1 episode per approximately 1 million hours of training
- 1 episode per approximately 500.000 hours of training
- 1 episode per approximately 100. 000 hours of training
- 1 episode per approximately 50.000 hours of training
- 1 episode per approximately 10. 000 hours of training
- Don't know

38. The risk of a fatal heart attack during vigorous exercise for non-symptomatic individuals is:

Choose one of the following responses:
- 1 episode per approximately 9,5 million hours of training
- 1 episode per approximately 4,3 million hours of training
- 1 episode per approximately 1,6 million hours of training
- 1 episode per approximately 900.000 hours of training

- 1 episode per approximately 385. 000 hours of training<br>- 1 episode per approximately 75.000 hours of training<br>- 1 episode per approximately 12. 300 hours of training<br>- Don't know
39. The risk of a heart attack during intense supervised training for cardiac patients is:<br><br>**Choose one of the following responses:**<br>- 1 episode per approximately 7,2 million hours of training<br>- 1 episode per approximately 3,6 million hours of training<br>- 1 episode per approximately 1,1 million hours of training<br>- 1 episode per approximately 650.000 hours of training<br>- 1 episode per approximately 195.000 hours of training<br>- 1 episode per approximately 58.000 hours of training<br>- 1 episode per approximately 9.600 hours of training<br>- Don't know
40. The "Maximum Oxygen Uptake" (VO2max) refers to:<br><br>**Choose one of the following responses:**<br>- Oxygen consumption circulated under exhaustion conditions during aerobic exercise<br>- Oxygen consumption circulated under exhaustion conditions during muscle strengthening exercise<br>- Oxygen consumption circulated at rest<br>- Oxygen consumption when we start to get tired<br>- Don't know
41. "One-Repetition Maximum" (1RM) is:<br><br>**Choose one of the following responses:**<br>- The maximum number of push-ups I can perform with the correct technique<br>- The maximum number of abdominals I can perform with the correct technique<br>- The maximum number of weights I can lift with correct technique in a weight training exercise<br>- One repetition when performing weight training exercises that has a maximum duration<br>- Don't know
42. Stretching exercises are:<br><br>**Choose one of the following responses:**<br>- Those that increase the length of a muscle<br>- The resistance exercises<br>- The balance exercises<br>- All the above<br>- Don't know
43. According to the World Health Organization's Global Action Plan on Physical Activity 2018-2030, the goal is to reduce global physical inactivity by:<br><br>**Choose one of the following responses:**<br>5%<br>10%<br>15%<br>20%

25%<br>- Don't know
44. According to the World Health Organization, what is the minimum number of days per week of moderate-intensity physical activity that adults should do to achieve health benefits?<br><br>**Choose one of the following responses:**<br>1<br>2<br>3<br>4<br>5<br>6<br>- Don't know
45.According to the World Health Organization, what is the minimum number of minutes of moderate-intensity aerobic physical activity recommended per week for adults and people with chronic NCDs (hypertension, type 2 diabetes, and cancer) to achieve health benefits?<br><br>**Choose one of the following responses:**<br>50<br>100<br>150<br>200<br>250<br>300<br>- Don't know
46.According to the World Health Organization, what is the minimum number of minutes of vigorous-intensity aerobic physical activity recommended per week for adults and people with chronic NCDs (hypertension, type 2 diabetes, and cancer) to achieve health benefits?<br><br>**Choose one of the following responses:**<br>50<br>75<br>100<br>125<br>150<br>175<br>- Don't know
47.According to the World Health Organization, what is the minimum number of workouts per week for muscle strengthening?<br><br>**Choose one of the following responses:**<br>1<br>2<br>3<br>4<br>5

6<br>- Don't know
48. Regular physical activity can benefit cancer patients as follows:<br><br>**Choose one of the following responses:**<br>- Improving fatality<br>- Improving the risk of recurrence<br>- Improving morbidity<br>- All the above<br>- Don't know
49. Regular physical activity can benefit patients with type 2 diabetes as follows:<br><br>**Choose one of the following responses:**<br>- Reducing mortality from cardiovascular disease<br>- Improving mortality<br>- Reducing the progression of the disease<br>- All the above<br>- Don't know
50. Physical inactivity is defined as moderate to vigorous physical activity lasting less than:<br><br>**Choose one of the following responses:**<br>-10 minutes daily<br>-20 minutes daily<br>-30 minutes daily<br>-40 minutes daily<br>-60 minutes daily<br>-Don't know
51. How can the physical activity and exercise level of a patient with a chronic non-communicable disease be assessed?<br><br>**Choose one of the following responses:**<br>-By physical activity questionnaires<br>-Accelerometers<br>-With pedometers<br>-With mobile phones<br>-All the above<br>-Don't know
52. Which questionnaire is used to assess physical activity levels over the last 7 days?<br><br>**Choose one of the following responses:**<br>- The Physical Activity Questionnaire for Osteoporosis (BONES PAS)<br>- The Global Physical Activity Questionnaire (GPAQ)<br>- The Global Physical Activity Questionnaire (GPAQ)<br>- All the above<br>- Don't know
Please note any comments you may have regarding the knowledge questions you answered.

III. Attitudes toward physical activity and exercise

Questions	Strongly Disagree	Disagree	Neutral	Agree	Strongly Agree
53. Physical activity and exercise are important for the prevention and treatment of chronic non-communicable diseases	1 ☐	2 ☐	3 ☐	4 ☐	5 ☐
54. Physical activity and exercise are necessary for the prevention and treatment of chronic non-communicable diseases	1 ☐	2 ☐	3 ☐	4 ☐	5 ☐
55. It is important for physicians to ask their patients whether they perform physical activity or exercise	1 ☐	2 ☐	3 ☐	4 ☐	5 ☐
56. It is important that physicians advise their patients about the benefits of physical activity and exercise	1 ☐	2 ☐	3 ☐	4 ☐	5 ☐
57. It is important that physicians, when appropriate, prescribe exercise to their patients with chronic non-communicable diseases	1 ☐	2 ☐	3 ☐	4 ☐	5 ☐
58. It is important for physicians to assess whether there is an exercise-related change in the health of their patients who exercise	1 ☐	2 ☐	3 ☐	4 ☐	5 ☐
59. It is important for physicians to know how to assess their patients' level of physical activity or fitness	1 ☐	2 ☐	3 ☐	4 ☐	5 ☐
60. Health promotion through physical activity and exercise is one of the physicians' duties	1 ☐	2 ☐	3 ☐	4 ☐	5 ☐
61. It is essential that a course on physical activity and exercise be taught in the undergraduate curriculum of medical students	1 ☐	2 ☐	3 ☐	4 ☐	5 ☐
62. It is essential that a course on physical activity and exercise be taught in the undergraduate curriculum of all Health Sciences students	1 ☐	2 ☐	3 ☐	4 ☐	5 ☐
63. Patients' behaviour towards physical activity and exercise improves after counselling by a physician	1 ☐	2 ☐	3 ☐	4 ☐	5 ☐
64. Physically active physicians act as a positive role model for their patients	1 ☐	2 ☐	3 ☐	4 ☐	5 ☐
65. Counselling on physical activity and exercise for people with chronic non-communicable diseases is a duty for health and exercise specialists	1 ☐	2 ☐	3 ☐	4 ☐	5 ☐
65a. Exercise counselling for patients with chronic non-communicable diseases is one of the duties of a physician	1 ☐	2 ☐	3 ☐	4 ☐	5 ☐

65b. Exercise counselling for patients with chronic non-communicable diseases is one of the duties of a qualified graduate in physical education	1 ☐	2 ☐	3 ☐	4 ☐	5 ☐
65c. Exercise counselling for patients with chronic non-communicable diseases is one of the duties of a qualified nurse	1 ☐	2 ☐	3 ☐	4 ☐	5 ☐
65d. Exercise counselling for patients with chronic non-communicable diseases is one of the duties of a clinical dietician	1 ☐	2 ☐	3 ☐	4 ☐	5 ☐
65e.Exercise counselling for patients with chronic non-communicable diseases is one of the duties of a physiotherapist	1 ☐	2 ☐	3 ☐	4 ☐	5 ☐
65f. Exercise counselling for patients with chronic non-communicable diseases is one of the duties of a psychologist	1 ☐	2 ☐	3 ☐	4 ☐	5 ☐
66. The design of exercise programs for patients with chronic non-communicable diseases is a duty of health and exercise specialists.	1 ☐	2 ☐	3 ☐	4 ☐	5 ☐
66a.The planning of exercise programs for patients with chronic non-communicable diseases is one of the duties of a physician	1 ☐	2 ☐	3 ☐	4 ☐	5 ☐
66b.The design of exercise programs for patients with chronic non-communicable diseases is one of the duties of a specialist physical education graduate	1 ☐	2 ☐	3 ☐	4 ☐	5 ☐
66c.The planning of exercise programs for patients with chronic non-communicable diseases is one of the duties of a qualified nurse	1 ☐	2 ☐	3 ☐	4 ☐	5 ☐
66d. The planning of exercise programs for patients with chronic non-communicable diseases is one of the duties of a clinical dietician	1 ☐	2 ☐	3 ☐	4 ☐	5 ☐
66e.The planning of exercise programs for patients with chronic non-communicable diseases is one of the duties of a physiotherapist	1 ☐	2 ☐	3 ☐	4 ☐	5 ☐
66f. The planning of exercise programs for patients with chronic non-communicable diseases is one of the duties of the psychologist	1 ☐	2 ☐	3 ☐	4 ☐	5 ☐
67. The implementation of exercise programs for patients with chronic non-communicable diseases is a duty of health and exercise specialists.	1 ☐	2 ☐	3 ☐	4 ☐	5 ☐
67a.The implementation of exercise programs for patients with chronic, non-communicable diseases is one of the duties of a physician	1 ☐	2 ☐	3 ☐	4 ☐	5 ☐
67b. The implementation of exercise programs for patients with chronic, non-communicable diseases is one of the duties of a qualified graduate in physical education	1 ☐	2 ☐	3 ☐	4 ☐	5 ☐

67c. The implementation of exercise programs for patients with chronic, non-communicable diseases is one of the duties of a qualified nurse	1 ☐	2 ☐	3 ☐	4 ☐	5 ☐
67d. The implementation of exercise programs for patients with chronic, non-communicable diseases is one of the duties of a clinical dietician	1 ☐	2 ☐	3 ☐	4 ☐	5 ☐
67e.The implementation of exercise programs for patients with chronic, non-communicable diseases is one of the duties of a physiotherapist	1 ☐	2 ☐	3 ☐	4 ☐	5 ☐
67f. The implementation of exercise programs for patients with chronic, non-communicable diseases is one of the duties of a psychologist	1 ☐	2 ☐	3 ☐	4 ☐	5 ☐
Please note any comments you may have regarding the attitude questions you answered.					

IV. Intentions on physical activity and exercise

Questions	Not at all	A little	Moderately	Rather much	Very much
68. I intend to ask or continue to ask my patients with chronic non-communicable diseases if they perform physical activity or exercise	1 ☐	2 ☐	3 ☐	4 ☐	5 ☐
69. I intend to assess or continue to assess the physical activity levels of my patients with chronic non-communicable diseases	1 ☐	2 ☐	3 ☐	4 ☐	5 ☐
70. I intend to counsel or continue to counsel on physical activity and exercise my patients with chronic non-communicable diseases	1 ☐	2 ☐	3 ☐	4 ☐	5 ☐
71. I intend to evaluate or continue to evaluate the impact of physical activity and exercise on the health of my patients with chronic non-communicable diseases	1 ☐	2 ☐	3 ☐	4 ☐	5 ☐
72. I would like to prescribe exercise to my patients with chronic non-communicable diseases	1 ☐	2 ☐	3 ☐	4 ☐	5 ☐
73. I intend to follow or continue to follow the progress of exercise programs for my patients with chronic non-communicable diseases	1 ☐	2 ☐	3 ☐	4 ☐	5 ☐
74. I intend to motivate or continue to motivate my patients with chronic non-communicable diseases to exercise	1 ☐	2 ☐	3 ☐	4 ☐	5 ☐
75. I intend to be actively educated on counselling patients with chronic non-communicable diseases on physical activity and exercise	1 ☐	2 ☐	3 ☐	4 ☐	5 ☐

Please note any comments you may have regarding the intentions questions you answered.

V. Abilities on physical activity and exercise

Questions	Not at all	A little	Moderately	Rather much	Very much
76. I feel able to counsel my patients with chronic non-communicable diseases on physical activity and exercise	1 ☐	2 ☐	3 ☐	4 ☐	5 ☐
77. I feel able to prescribe exercise to my patients with chronic non-communicable diseases	1 ☐	2 ☐	3 ☐	4 ☐	5 ☐
78. I feel able to assess the physical activity level in my patients with chronic non-communicable diseases	1 ☐	2 ☐	3 ☐	4 ☐	5 ☐
79. I feel able to assess the impact of physical activity and exercise on the health of my patients with chronic non-communicable diseases after my consultation	1 ☐	2 ☐	3 ☐	4 ☐	5 ☐
80.I feel able to follow the progress of the exercise programs of my patients with chronic non-communicable diseases	1 ☐	2 ☐	3 ☐	4 ☐	5 ☐
81. I feel able to motivate my patients with chronic non-communicable diseases to exercise or perform physical activity	1 ☐	2 ☐	3 ☐	4 ☐	5 ☐
Please note any comments you may have regarding the abilities questions you answered.					

VI. Behaviour on physical activity and exercise

Questions	Never	Rarely	Sometimes	Often	Always
82. I ask my patients with chronic non-communicable diseases if they exercise or perform physical activity	1 ☐	2 ☐	3 ☐	4 ☐	5 ☐
83. I assess physical activity levels in my patients with chronic non-communicable diseases	1 ☐	2 ☐	3 ☐	4 ☐	5 ☐
84. I counsel my patients about physical activity and exercise and their health benefits	1 ☐	2 ☐	3 ☐	4 ☐	5 ☐
84a. If yes, my patient's average consultation time per visit is:	**Choose one of the following responses:**<br><5 minutes<br>5-10 minutes				

	>10 minutes<br>Don't remember				
84b. If yes, the usual way of consulting my patients is mainly:	**Choose one of the following responses:**<br>- Verbal<br>- Written<br>- Verbal and written<br>- Other<br>- Don't remember				
84c. If yes, the content of my counselling is:	**Choose one or more of the following responses**:<br>1. Benefits of physical activity and exercise on health in general<br>2. Benefits of physical activity and exercise on chronic non-communicable diseases in general health<br>3. Type of physical activity/exercise<br>4. Intensity of physical activity/exercise<br>5. Duration of physical activity/exercise<br>6. Frequency of physical activity/ exercise<br>7. Don't remember				
84d. If yes, I counsel my patients about the following chronic, non-communicable diseases	**Choose one or more of the following responses**:<br>1. Obesity<br>2. Hypertension<br>3. Diabetes<br>4. Lipid problems or metabolic syndrome<br>5. Kidney diseases<br>6. Cardiovascular diseases<br>7. Respiratory diseases<br>8. Cancer<br>9. Arthritis<br>10. Autoimmune diseases<br>11. Osteoporosis<br>12. Neurological diseases<br>13. Psychiatric diseases<br>14. Other				
**Questions**	**Never**	**Rarely**	**Sometimes**	**Often**	**Always**
84e. If yes, I evaluate the effect of physical activity and exercise on the health of my patients after my counselling	1	2	3	4	5
84f. If yes, I positively influence my patients on physical activity and exercise with my counselling	1	2	3	4	5
85. There are factors that facilitate or would facilitate exercise counselling on my patients with chronic non-communicable diseases	1	2	3	4	5
85a. Factors that facilitate exercise counselling are:	**Choose one or more of the following responses:**<br>1. Adequate time				

	2. Training in physical activity and exercise counselling skills<br>3. My prior knowledge of physical activity and exercise<br>4. My patients' increased interest in lifestyle change<br>5. Existence of financial motivation<br>6. My continuing education in these areas<br>7. Other				
**Questions**	**Never**	**Rarely**	**Sometimes**	**Often**	**Always**
86. There are barriers (or potential barriers) to exercise counselling on my patients with chronic non-communicable diseases	1 ☐	2 ☐	3 ☐	4 ☐	5 ☐
86a. Barriers I encounter in exercise counselling:	**Choose one or more of the following responses**:<br>1. Lack of my own time<br>2. Lack of patient time<br>3. Lack of counselling skills<br>4. Lack of training<br>5. Lack of financial motivation<br>6. Reduced interest of patients in changing their lifestyle<br>7. Patients' preference for a drug rather than exercise<br>8. Preference to advise on other healthy lifestyle issues rather than exercise<br>9. Lack of exercise counselling guidelines/protocols for physicians<br>10. Other				
**Questions**	**Never**	**Rarely**	**Sometimes**	**Often**	**Always**
87. I prescribe exercise to my patients with chronic non-communicable diseases	1 ☐	2 ☐	3 ☐	4 ☐	5 ☐
87a. There are factors that facilitate or would facilitate the exercise prescription in patients with chronic non-communicable diseases	1 ☐	2 ☐	3 ☐	4 ☐	5 ☐
87b. Factors that facilitate or would facilitate the prescription of exercise are:	**Choose one or more of the following responses:**<br>1. Excess time of physicians<br>2. Excess time of patients<br>3. Training of physicians<br>4. Availability of appropriate applications for physicians<br>5. Continuous training of physicians<br>6. Existence of a financial incentive<br>7. Increased interest of patients<br>8. Adoption of appropriate legislation				

	9. Other				
**Questions**	Never	Rarely	Sometimes	Often	Always
87c. There are barriers (or potential barriers) in prescribing exercise to patients with chronic non-communicable diseases.	1 ☐	2 ☐	3 ☐	4 ☐	5 ☐
87d. Barriers I encounter in prescribing exercise:	**Choose one or more of the following responses:**<br>1. Lack of physicians' time<br>2. Lack of patients' time<br>3. Lack of training<br>4. Lack of applications for physicians<br>5. Lack of continuing education<br>6. Lack of financial incentive<br>7. Decreased interest of my patients<br>8. Patients' preference for medication rather than exercise.<br>9. The lack of appropriate legislation<br>10. Other				
**Questions**	**Never**	**Rarely**	**Sometimes**	**Often**	**Always**
88. I follow the progress of exercise programs of my patients with chronic, non-communicable disease	1 ☐	2 ☐	3 ☐	4 ☐	5 ☐
89. I refer my patients with chronic non-communicable diseases for counselling, planning, and implementation of health exercise programs to other specialists	1 ☐	2 ☐	3 ☐	4 ☐	5 ☐
89a. If yes, I refer them to a qualified graduate in physical education	1 ☐	2 ☐	3 ☐	4 ☐	5 ☐
89b. If yes, I refer them to a qualified nurse	1 ☐	2 ☐	3 ☐	4 ☐	5 ☐
89c. If yes, I refer them to a clinical dietitian	1 ☐	2 ☐	3 ☐	4 ☐	5 ☐
89d. If yes, I refer them to a physiotherapist	1 ☐	2 ☐	3 ☐	4 ☐	5 ☐
89e. If yes, I refer them to a psychologist	1 ☐	2 ☐	3 ☐	4 ☐	5 ☐
90. I motivate my patients with chronic non-communicable diseases to exercise	1 ☐	2 ☐	3 ☐	4 ☐	5 ☐
In addition to the above-mentioned questions, please record any questions or suggestions you consider important to include in the final questionnaire to adequately investigate physicians' behaviour regarding physical activity and exercise.					

VII. Questions to test face validity

91. Please note the time you need to complete the questionnaire	
<10 minutes	☐
10-20minutes	☐
20-30minutes	☐
>30 minutes	☐

92. What is the difficulty level in understanding the questions (difficulty/clarity of words, concepts, and phrases)?	Zero	Small	Medium	High	Very high
	☐	☐	☐	☐	☐

93. Do you consider the subject matter and content of the questionnaire to be according to the aim of its development?	Not at all	A little	Moderately	Rather much	Very much
	☐	☐	☐	☐	☐

94. Do you believe the questions cover a wide range of physicians' knowledge, attitudes, intentions, abilities and behaviours toward physical activity and exercise?	Not at all	A little	Moderately	Rather much	Very much
	☐	☐	☐	☐	☐

95. Do you think any questions are unnecessary, incomplete, or excessive?	
Yes	☐
No	☐

96. Please fill in if you have any comments regarding the structure and content of this questionnaire.	

Table S5. Demographic professional, and educational characteristics of physicians at stage 2 (N=61).

Question	Category	n	%
1. Gender	male	38	62.3
	female	23	37.7
2. Age	30-39	10	16.4
	40-49	34	55.7
	50-59	14	23.0
	>60	3	4.9
3. Family status	unmarried	6	9.8
	married	51	83.6
	divorced	2	3.3
	widowed	1	1.6
	cohabitation	1	1.6
4. Do you have children?	no	11	18.0
	yes	50	82.0
5. In what area have you been living permanently for the last few years?	village	4	6.6
	town	2	3.3
	city	55	90.2
6. What is your medical specialty?	internal medicine	14	23.0
	cardiology	11	18.0
	general medicine	9	14.8
	rheumatology	4	6.6
	endocrinology	7	11.5
	neurology	4	6.6
	psychiatry	3	4.9
	nephrology	2	3.3
	orthopedics	4	6.6
	pulmonology	2	3.3
	vascular surgery	1	1.6
	oncology	-	-
8. At which university did you do your undergraduate studies?	international	9	14.8
	greek	52	85.2
8a. If you studied at an international or other university, which one; (N=8)	Bulgaria	4	6.4
	Italy	2	3.2
	Bucharest	1	1.6
	Brussels	1	1.6
9. Do you have any other studies or specializations (other than your major specialty)?	no	26	42.6
	yes	35	57.4
9a. If yes, what are they? (N=36)	MSc	16	26.2
	PhD	11	18.0
	Bachelor	1	1.6
	Other degree	8	13.1
9b. If you answered others, what are they? Please specify (N=51)	aimostasis	1	1.6
	acupuncture	1	1.6

	diabetology - biomedical acupuncture	1	1.6
	intensive Care Unit	1	1.6
	metabolic bone diseases	1	1.6
	pediatric cardiology-fetal cardiology	1	1.6
	ultrasound	2	3.2
	clinical microbiology	1	1.6
10. What is your employment status?	private	49	80.3
	public	11	18.0
	other	1	1.6
10a. If you answered other, which one is it? Please specify (N=1)	OKANA	1	1.6
11. What region do you work in?	village	2	3.3
	town	10	16.4
	city	49	80.3
11a. Which Medical Association do you belong to?	Trikala	35	57.4
	Karditsa	26	42.6
12. How many patients do you see in one day on average?	<5	1	1.6
	5-10	17	27.9
	11-20	23	37.7
	>20	17	27.9
	don't remember	2	3.3
13. Compared to other people your age, would you say that your health condition is:	bad	1	1.6
	moderate	8	13.1
	very good	40	65.6
	excellent	12	19.7
14. Compared to other people your age, would you say that your physical condition/ability is:	bad	7	11.5
	moderate	22	36.1
	very good	29	47.5
	excellent	3	4.9
15. Do you smoke?	no, never	37	60.7
	yes	14	23.0
	no, I recently quit smoking (in the last 6 months)	1	1.6
	no, I quit smoking at least 1 year ago	9	14.8
15a. If yes, how many cigarettes per day do you smoke? (N=15)	<10	4	6.6
	11-20	7	11.5
	21-30	2	3.3
	I don't smoke every day, but occasionally during the week or month	2	3.3
16. Have you ever been taught physical activity and exercise subject(s) at the medical school you attended?	no	29	47.5
	yes	7	11.5
	don't remember	25	41.0

16a. If yes, which physical activity and exercise subject(s) have you been taught? Please state. (N=4)	physiology	1	1.6
	resistance / aerobic in malignancies, aerobic exercise in NAFLD / metabolic syndrome	1	1.6
	exercise, walking	1	1.6
	physical activity and exercise in patients with diabetes mellitus	1	1.6
16b. If not, which physical activity and exercise module(s) would you prefer to have been taught? Please briefly state (N=25).	exercise mechanics, exercise biochemistry, exercise and health, illness and exercise, sport-specific exercises	1	1.6
	aerobic gymnastics	1	1.6
	strengthening of the musculoskeletal system	1	1.6
	exercise and health, exercise biochemistry	1	1.6
	exercise in chronic health problems - nutrition and exercise	1	1.6
	exercise and the benefits to life and health	1	1.6
	training of patients in forms of exercise according to the underlying disease	1	1.6
	exercise in relation to medical diseases	1	1.6
	aerobic capacity, resistance exercise in clinical populations	1	1.6
	cardiovascular and muscular strengthening exercises	1	1.6
	mode of exercise for urban people, outside of gyms	1	1.6

	physical activity and pathophysiology of diseases	1	1.6
	activity in chronic diseases, such as diabetes mellitus and cardiovascular diseases	1	1.6
	aerobic exercise	1	1.6
	aerobics, swimming	1	1.6
	metabolism and clinical nutrition	1	1.6
	aerobic	1	1.6
	physical rehabilitation	1	1.6
	recreational physical activity and exercise	1	1.6
	aerobic exercise, athletics	1	1.6
	cardiovascular system and exercise	1	1.6
	exercise protocols related to specific conditions, ways to support/promote exercise for patients	1	1.6
	physiology of exercise	1	1.6
	aerobic exercise-muscular strengthening	1	1.6
	exercise and its effect on the immune system	1	1.6
17. Are you informed/educated about physical activity and exercise issues in the context of lifelong learning?	no<br>yes	21<br>40	34.4<br>65.6
17a. If yes, please indicate the sources from which you are informed/educated about physical activity and exercise (one or more responses) (N=42)	scientific journals and books<br>internet<br>media<br>seminars/ conferences<br>lifelong learning programs<br>other	22<br>33<br>5<br>23<br>5<br>0	52.4<br>78.6<br>11.9<br>54.8<br>11.9<br>0
17b. If you have already participated in a seminar or lifelong	<1<br>1-3	3<br>4	10.7<br>14.3

learning program in physical activity and exercise, how many hours of training have you participated in? (N=28)	4-10<br>11-40<br>>40<br>don’t remember	2<br>1<br>0<br>18	7.1<br>3.6<br>0<br>64.3
17c. If you have attended a seminar or lifelong learning programmes on physical activity and exercise, in which subject(s) have you been trained? Please state. (N=2)	exercise for kidney patients<br>respiratory rehabilitation	1<br>1	1.6<br>1.6
18. Do you wish to participate in a seminar or training program on physical activity and exercise in the future?	no<br>yes	12<br>49	19.7<br>80.3
18a. If yes, what kind of training would you like to participate in? (N=51)	synchronous distance<br>live<br>hybrid<br>other	13<br>18<br>19<br>1	21.3<br>29.5<br>31.1<br>1.6
18a1. If you answered another, which one is it? Please specify. (N=1)	synchronous and asynchronous distance	1	1.6
18b. If yes, how many hours would you like to be trained in a physical activity and exercise training seminar or programme? (N=51)	<1<br>1-3<br>4-10<br>11-40<br>>40	0<br>14<br>26<br>7<br>4	0<br>23.0<br>42.6<br>11.5<br>6.6
18c. If yes, in which subject(s) would you like to be trained? Please state. (N=12)	vascular strokes and exercise	1	1.6
	exercise and children; pregnancy and exercise; swimming	1	1.6
	metabolism, clinical nutrition, and exercise	1	1.6
	aerobics exercise	1	1.6
	physical rehabilitation	1	1.6
	prescription of physical exercise; indications and contraindications	1	1.6
	recreational physical activity; exercise to prevent and improve diabetes mellitus, obesity and cardiovascular disease in adults and children; exercise in pregnant women to prevent and treat gestational diabetes.	1	1.6

	cardiovascular disease and exercise	1	1.6
	exercise endocrinology	1	1.6
	rehabilitation programs after myocardial infarction and bypass	1	1.6
	exercise physiology; modern training	1	1.6
	exercise in spinal disorders; exercise in stroke; exercise in Parkinson's disease	1	1.6
19. Do you know the benefits of physical activity and exercise on chronic non-communicable diseases?	no<br>yes	6<br>55	9.8<br>90.2
19a. If yes, what is your level of knowledge?	very low<br>low<br>medium<br>high<br>very high	-<br>5<br>22<br>26<br>2	-<br>8.2<br>36.1<br>42.6<br>3.3
20. Do you know about counselling patients with chronic non-communicable diseases on physical activity and exercise?	no<br>yes	15<br>46	24.6<br>75.4
20a. If yes, what is your level of knowledge?	very low<br>low<br>medium<br>high<br>very high	2.1<br>14.6<br>50.0<br>27.1<br>6.3	1.6<br>11.5<br>39.3<br>21.3<br>4.9
21. Do you know about prescribing physical activity and exercise for patients with chronic non-communicable diseases?	no<br>yes	40<br>21	65.6<br>34.4
21a. If yes, what is your level of knowledge?	very low<br>low<br>medium<br>high<br>very high	1<br>6<br>14<br>6<br>1	1.6<br>9.8<br>23.0<br>9.8<br>1.6
22. Do you know about assessing physical activity and exercise levels in patients with chronic non-communicable diseases?	no<br>yes	31<br>30	50.8<br>49.2
22a. If yes, what is your level of knowledge?	very low<br>low<br>medium<br>high	1<br>8<br>16<br>9	1.6<br>13.1<br>26.2<br>14.8

	very high	-	-
23. Are you aware of the World Health Organisation (WHO) guidelines on physical activity and health?	no<br>yes	35<br>26	57.4<br>42.6
**Question**	**Median**	**IQR**	**Min-Max**
6a. What is the year you obtained your speciality?	2008.5	10	1997-2019
7. How many years have you been working as a physician?	22.5	14	9-30
15b. If you only smoked in the past, how long did you smoke (in years)?	13.5	5	7-15

Min-max, minimum-maximum values; n, absolute frequencies; %, relative frequencies; Mdn, median value; IQR, interquartile range; N, number of physicians who answered the optional questions.

Table S6. Physicians' knowledge of physical activity and exercise towards non-communicable diseases at stage 2 (N=61).

**Question**	**Correct answer/ n(%)**	**Wrong answer/ n(%)**	**Don't know/ n(%)**
24. Physical activity is:	51/(83.6)	9/(14.8)	1/(1.6)
25. Exercise is:	52/(85.2)	8/(13.1)	1/(1.6)
26. When we say, "major principles of exercise program design", we mean:	43/(70.5)	15/(24.6)	3/(4.9)
27. The intensity of the exercise is:	50/(82.0)	6/(9.8)	5/(8.2)
28. The duration of the exercise is:	56/(91.8)	4/(6.6)	1/(1.6)
29. The frequency of exercise is:	59/(96.7)	1/(1.6)	1/(1.6)
30. Which of the following is an indicator of exercise intensity?	38/(62.3)	18/(29.5)	5/(8.2)
31. Which of the following is a resistance training exercise?	12/(19.7)	42/(68.9)	7/(11.5)
32. Which of the following is aerobic exercise?	46/(75.4)	13/(21.3)	2/(3.3)
33. Which of the following is a mobility exercise?	30/(49.2)	16/(26.2)	15/(24.6)
34. Which of the following is a high-intensity interval exercise?	47/(77.0)	6/(9.8)	8/(13.1)
35. "PAR-Q and YOU" are:	13/(21.3)	1/(1.6)	47/(77.0)
36. The "MET" is:	19/(31.1)	0	42/(68.9)
37. The risk of a non-fatal heart attack during vigorous exercise in asymptomatic individuals is:	0	9/(14.8)	52/(85.2)

38. The risk of a fatal heart attack during vigorous exercise for non-symptomatic individuals is:	1/(1.6)	6/(9.8)	54/(88.5)
39. The risk of a heart attack during intense supervised training for cardiac patients is:	0	7/(11.5)	54/(88.5)
40. The "Maximum Oxygen Uptake" (VO2max) refers to:	34/(55.7)	9/(14.8)	18/(29.5)
41. "One-Repetition Maximum" (1RM) is:	12/(19.7)	17/(27.9)	32/(52.5)
42. Stretching exercises are:	45/(73.8)	9/(14.8)	7/(11.5)
43. According to the World Health Organisation's Global Action Plan on Physical Activity 2018-2030, the goal is to reduce global physical inactivity by:	0	18/(29.5)	43/(70.5)
44. According to the World Health Organisation, what is the minimum number of days per week of moderate-intensity physical activity that adults should do to achieve health benefits?	25/(41.0)	36/(59.0)	0
45. According to the World Health Organisation, what is the minimum number of minutes of moderate-intensity aerobic physical activity recommended per week for adults and people with chronic NCDs (hypertension, type 2 diabetes, and cancer) to achieve health benefits?	21/(34.4)	24/(39.3)	16/(26.2)
46. According to the World Health Organisation, what is the minimum number of minutes of vigorous-intensity aerobic physical activity recommended per week for adults and people with chronic NCDs (hypertension, type 2 diabetes, and cancer) to achieve health benefits?	10/(16.4)	28/(45.9)	23/(37.7)
47. According to the World Health Organisation, what is the minimum number of workouts per week for muscle strengthening?	12/(19.7)	24/(39.3)	25/(41.0)
48. Regular physical activity can benefit cancer patients as follows:	42/(68.9)	8/(13.1)	11/(18.0)
49. Regular physical activity can benefit patients with type 2 diabetes as follows:	53/(86.9)	5/(8.2)	3/(4.9)
50. Physical inactivity is defined as moderate to vigorous physical activity lasting less than:	5/(8.2)	33/(54.1)	23/(37.7)
51. How can the physical activity and exercise level of a patient with a chronic non-communicable disease be assessed?	36/(59.0)	14/(23.0)	11/(18.0)
52. Which questionnaire assesses physical activity levels over the last 7 days?	5/(8.2)	16/(26.2)	40/(65.6)

n, absolute frequencies; %, relative frequencies; NCDs, non-communicable diseases; N, number of participants.

Table S7. Physicians' attitudes on physical activity and exercise towards non-communicable diseases at stage 2 (N=61).

Question	Strongly Agree/ n(%)	Agree/ n(%)	Neutral/ n(%)	Disagree/ n(%)	Strongly Disagree/ n(%)
53. Physical activity and exercise are important for the prevention and treatment of chronic non-communicable diseases	51/(83.6)	9/(14.8)	1/(1.6)	0	0
54. Physical activity and exercise are necessary for the prevention and treatment of chronic non-communicable diseases	47/(77.0)	11/(18.1)	3/(4.9)	0	0
55. It is important for physicians to ask their patients whether they perform physical activity or exercise	48/(78.7)	9/(14.8)	4/(6.6)	0	0
56. It is important that physicians advise their patients about the benefits of physical activity and exercise	52/(85.2)	8/(13.1)	1/(1.6)	0	0
57. It is important that physicians, when appropriate, prescribe exercise to their patients with chronic non-communicable diseases	34/(55.7)	16/(26.2)	10/(16.4)	0	1/(1.6)
58. It is important for physicians to assess whether there is an exercise-related change in the health of their patients who exercise	37/(60.7)	18/(29.5)	5/(8.2)	1/(1.6)	0
59. It is important for physicians to know how to assess their patients' level of physical activity or fitness	32/(52.5)	21/(34.4)	7/(11.5)	1/(1.6)	0
60. Health promotion through physical activity and exercise is one of the physicians’ duties	30/(49.2)	21/(34.4)	9/(14.8)	1/(1.6)	0
61. It is essential that a course on physical activity and exercise be taught in the undergraduate curriculum of medical students	40/(65.6)	14/(23.0)	6/(9.8)	1/(1.6)	0
62. It is essential that a course on physical activity and exercise be taught in the undergraduate	38/(62.3)	15/(24.6)	7/(11.5)	1/(1.6)	0

curriculum of all Health Sciences students					
63. Patients' behaviour towards physical activity and exercise improves after counselling by a physician	22/(36.1)	21/(34.4)	17/(27.9)	1/(1.6)	0
64. Physically active physicians act as positive role models for their patients	27/(44.3)	21/(34.4)	12/(19.7)	1/(1.6)	0
65. Counselling on physical activity and exercise for people with chronic non-communicable diseases is a duty for health and exercise specialists	25/(41.0)	21/(34.4)	15/(24.6)	0	0
65a. Exercise counselling for patients with chronic non-communicable diseases is one of the duties of a physician	24/(39.3)	22/(36.1)	14/(23)	1/(1.6)	0
65b. Exercise counselling for patients with chronic non-communicable diseases is one of the duties of a qualified graduate in physical education	34/(55.7)	18/(29.5)	9/(14.8)	0	0
65c. Exercise counselling for patients with chronic non-communicable diseases is one of the duties of a qualified nurse	13/(21.3)	20/(32.8)	21/(34.4)	6/(9.8)	1/(1.6)
65d. Exercise counselling for patients with chronic non-communicable diseases is one of the duties of a clinical dietician	24/(39.3)	16/(26.2)	15/(24.6)	4/(6.6)	2/(3.3)
65e.Exercise counselling for patients with chronic non-communicable diseases is one of the duties of a physiotherapist	28/(45.9)	16/(26.2)	14/(23.0)	2/(3.3)	1/(1.6)
65f. Exercise counselling for patients with chronic non-communicable diseases is one of the duties of a psychologist	18/(29.5)	17/(27.9)	17/(27.9)	6/(9.8)	3/(4.9)
66. The design of exercise programs for patients with chronic non-communicable diseases is a duty of health and exercise specialists	38/(62.3)	18/(29.5)	3/(4.9)	2/(3.3)	0
66a.The planning of exercise programs for patients with	17/(27.9)	15/(24.6)	14/(23.0)	12/(19.7)	3/(4.9)

chronic non-communicable diseases is one of the duties of a physician					
66b. The design of exercise programs for patients with chronic non-communicable diseases is one of the duties of a specialist physical education graduate	37/(60.7)	20/(32.8)	3/(4.9)	0	1/(1.6)
66c.The planning of exercise programs for patients with chronic non-communicable diseases is one of the duties of a qualified nurse	11/(18.1)	15/(24.6)	17/(27.9)	6/(9.8)	12/(19.7)
66d. The planning of exercise programs for patients with chronic non-communicable diseases is one of the duties of a clinical dietician	12/(19.7)	11/(18.1)	18/(29.5)	10/(16.4)	10/(16.4)
66e.The planning of exercise programs for patients with chronic non-communicable diseases is one of the duties of a physiotherapist	20(32.8)	22(36.1)	12(19.7)	2(3.3)	5(8.2)
66f. The planning of exercise programs for patients with chronic non-communicable diseases is one of the duties of the psychologist	7(11.5)	7(11.5)	21(34.4)	21(34.4)	12(19.7)
67. The implementation of exercise programs for patients with chronic non-communicable diseases is a duty of health and exercise specialists	34(55.7)	18(29.5)	6(9.8)	3(4.9)	0
67a.The implementation of exercise programs for patients with chronic, non-communicable diseases is one of the duties of a physician	13(21.3)	10(16.4)	17(27.9)	15(24.6)	6(9.8)
67b. The implementation of exercise programs for patients with chronic, non-communicable diseases is one of the duties of a qualified graduate in physical education	36(59.0)	17(27.9)	7(11.5)	0	1(1.6)
67c. The implementation of exercise programs for patients	11(18.0)	10(16.4)	21(34.4)	9(14.8)	10(16.4)

with chronic, non-communicable diseases is one of the duties of a qualified nurse					
67d. The implementation of exercise programs for patients with chronic, non-communicable diseases is one of the duties of a clinical dietician	9(14.8)	7(11.5)	18(29.5)	16(26.2)	11(18.0)
67e.The implementation of exercise programs for patients with chronic, non-communicable diseases is one of the duties of a physiotherapist	23(37.7)	17(27.9)	12(19.7)	6(9.8)	3(4.9)
67f. The implementation of exercise programs for patients with chronic, non-communicable diseases is one of the duties of a psychologist	7(11.5)	6(9.8)	17(27.9)	18(29.5)	13(21.3)

n, absolute frequencies; %, relative frequencies; NCDs, non-communicable diseases; N, number of participants.

Table S8. Physicians' intentions on physical activity and exercise towards non-communicable diseases at stage 2 (N=61).

**Question**	**Very much/ n(%)**	**Rather much/ n(%)**	**Moderately/ n(%)**	**Little/ n(%)**	**Not at all/ n(%)**
68. I intend to ask or continue to ask my patients with chronic non-communicable diseases if they perform physical activity or exercise	39/(63.9)	17/(27.9)	5/(8.2)	0	0
69. I intend to assess or continue to assess the physical activity levels of my patients with chronic non-communicable diseases	26/(42.6)	21/(34.4)	12/(19.7)	2/(3.3)	0
70. I intend to counsel or continue to counsel on physical activity and exercise my patients with chronic non-communicable diseases	38/(62.3)	19/(31.1)	4/(6.6)	0	0
71. I intend to evaluate or continue to evaluate the impact of physical activity and exercise on the health of my patients with chronic non-communicable diseases	29/(47.5)	23/(37.7)	7/(11.5)	2/(3.3)	0

72. I would like to prescribe exercise to my patients with chronic non-communicable diseases	26/(42.6)	18/(29.5)	8/(13.1)	6/(9.8)	3/(4.9)
73. I intend to follow or continue to follow the progress of exercise programs for my patients with chronic non-communicable diseases	22/(36.1)	17/(27.9)	18/(29.5)	4/(6.6)	0
74. I intend to motivate or continue to motivate my patients with chronic non-communicable diseases to exercise	46/(75.4)	13/(21.3)	2/(3.3)	0	0
75. I intend to be actively educated on counselling patients with chronic non-communicable diseases on physical activity and exercise	31/(50.8)	17/(27.9)	12/(19.7)	1/(1.6)	0

n, absolute frequencies; %, relative frequencies; NCDs, non-communicable diseases; N, number of participants.

Table S9. Physicians' abilities on physical activity and exercise towards non-communicable diseases at stage 2 (N=61).

Question	Very much/ n(%)	Rather much/ n(%)	Moderately/ n(%)	Little/ n(%)	Not at all/ n(%)
76. I feel able to counsel my patients with chronic non-communicable diseases on physical activity and exercise	9/(14.8)	24/(39.3)	18/(29.5)	9/(14.8)	1/(1.6)
77. I feel able to prescribe exercise to my patients with non-communicable diseases	6/(9.8)	19/(31.1)	19/(31.1)	13/(21.3)	4/(6.6)
78. I feel able to assess the physical activity level in my patients with chronic non-communicable diseases	5/(8.2)	21/(34.4)	19/(31.1)	14/(23.0)	2/(3.3)
79. I feel able to assess the impact of physical activity and exercise on the health of my patients with chronic non-communicable diseases after my consultation	6/(9.8)	24/(39.3)	20/(32.8)	9/(14.8)	2/(3.3)
80. I feel able to follow the progress of the exercise programs of my patients with chronic non-communicable diseases	4/(6.6)	13/(21.3)	27/(44.3)	13/(21.3)	4/(6.6)
81. I feel able to motivate my patients with chronic non-	23/(37.7)	21/(34.4)	13/(21.3)	3/(4.9)	1/(1.6)

communicable diseases to exercise or perform physical activity					

n, absolute frequencies; %, relative frequencies; NCDs, non-communicable diseases; N, number of participants.

Table S10. Physicians' behaviour (basic Likert-type scale) on physical activity and exercise towards non-communicable diseases at stage 2 (N=61).

**Question**	**Always/ n(%)**	**Often/ n(%)**	**Sometimes/ n(%)**	**Rarely/ n(%)**	**Never/ n(%)**
82. I ask my patients with chronic non-communicable diseases if they exercise or perform physical activity	35/(57.4)	16/(26.2)	8/(13.1)	2/(3.3)	0
83. I assess physical activity levels in my patients with chronic non-communicable diseases	10/(16.4)	23/(37.7)	17/(27.9)	9/(14.8)	2/(3.3)
84. I counsel my patients about physical activity and exercise and their health benefits	33/(54.1)	20/(32.8)	7/(11.5)	0	0
84e. I evaluate the effect of physical activity and exercise on the health of my patients after my counselling	12/(19.7)	22/(36.1)	15/(24.6)	11/(18.1)	1/(1.6)
84f. If yes, I positively influence my patients on physical activity and exercise with my counselling	11/(18.1)	23/(37.7)	25/(41)	2/(3.3)	0
87. I prescribe exercise to my patients with chronic non-communicable diseases	3/(4.9)	6/(9.8)	8/(13.1)	9/(14.8)	35/(57.4)
88. I follow the progress of exercise programs of my patients with chronic non-communicable disease	8/(13.1)	13/(21.3)	17/(27.9)	14/(23)	9/(14.8)
89. I refer my patients with chronic non-communicable diseases for counselling, planning, and implementation of health exercise programs to other specialists	11/(18.1)	20/(32.8)	14/(23)	8/(13.1)	8/(13.1)
90. I motivate my patients with chronic non-communicable diseases to exercise	33(54,1)	20/(32,8)	7 (11,5)	7(1,6)	0

n, absolute frequencies; %, relative frequencies; NCDs, non-communicable diseases; N, number of participants.
